# Genome-wide chromatin recording resolves dynamic cell state changes

**DOI:** 10.1016/j.cels.2026.101717

**Published:** 2026-09-02

**Authors:** Yodai Takei, Jordan A. Lay, James M. Linton, Duncan M. Chadly, Yoshiki Ochiai, Ron Hadas, Andrew A. Perez, Mario R. Blanco, Paola Laurino, Mitchell Guttman, Michael B. Elowitz

**Affiliations:** 1 Division of Biology and Biological Engineering, California Institute of Technology, Pasadena, CA, USA.; 2 Biohub, New York, NY, USA.; 3 Protein Engineering and Evolution Unit, Okinawa Institute of Science and Technology Graduate University, Onna, Okinawa, Japan.; 4 Institute for Protein Research, Osaka University, Suita-shi, Osaka, Japan.; 5 Howard Hughes Medical Institute, California Institute of Technology, Pasadena, CA, USA.; 6 Present address: Department of Biology, Massachusetts Institute of Technology, Cambridge, MA, USA.; 7 Present address: HAYA Therapeutics, San Diego, CA, USA.; 8 Lead contact

## Abstract

Understanding how the chromatin state of a cell influences its future behavior is a major challenge throughout biology. However, most chromatin profiling methods are limited to endpoint assays. Here, we present LagTag, a method for recovery of earlier and endpoint chromatin states in the same mammalian cells. In this approach, transient expression of bacterial adenine methyltransferase fusions records the DNA binding profiles of chromatin-associated proteins of interest at earlier timepoints. Subsequent tagmentation and sequencing recovers the earlier chromatin profile from adenine methylation profiles, alongside endpoint profiles of endogenous chromatin-associated proteins. We verified that LagTag profiles aligned with those from established methods in mouse and human cells. We then applied LagTag to record and recover dynamic chromatin state transitions during mouse embryonic stem cell differentiation, capturing transcriptional signatures from pre- and post-differentiation timepoints within the same cell population. LagTag thus provides a foundation for temporally resolved chromatin profiling. A record of this paper’s Transparent Peer Review process is included in the Supplemental Information.

## Electronic table of contents (eTOC) blurb

Takei et al. introduce LagTag, a chromatin-based recording strategy for mammalian cells. Past chromatin states are recorded by time-gated m6A writing and recovered alongside present states from the same cell population by antibody-guided tagmentation, establishing a foundation for temporally resolved chromatin profiling.

## Graphical Abstract

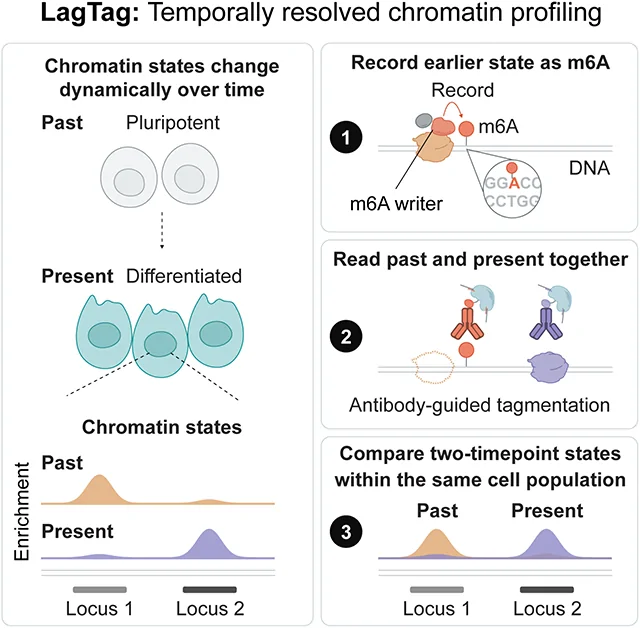

## Introduction

Chromatin states influence subsequent gene regulation and cellular behavior. They impact signal responses, cell fate decisions, and disease progression^1^. Knowing the causal relationships between earlier chromatin states and subsequent cellular behaviors is therefore critical for understanding, predicting, and controlling gene regulation and cell states. Existing approaches for mapping chromatin states generally provide static “snapshots” from single endpoint measurements^2^, providing no direct readout of earlier states. The ability to recover information from multiple timepoints in the same cells would reveal dynamic relationships that are otherwise difficult to infer. Temporally integrated measurements could reveal if and when pre-existing chromatin states influence cell fate decision-making. They could also identify chromatin states that lead to cancer drug tolerance^3^. An additional advantage of chromatin-based recording is its ability to capture various repressive chromatin states^4^ at transcriptionally inactive loci, information that is not attainable through transcription-based recording alone^5,6^.

Recent work has attempted to address this fundamental challenge in different ways. Pseudotime approaches can infer likely developmental trajectories of individual cells based on cell state similarity in static snapshots^7^. However, these approaches do not provide information from multiple timepoints in the same cell^8^. RNA velocity can provide insights into the instantaneous rates of change of RNA species in a cell^7,9^, but this is distinct from capturing a specific earlier timepoint and can be unreliable^10^. In the Rewind method^11^, inherited barcodes allow one to compare cells at a later timepoint to the state of their siblings at an earlier timepoint. However, this approach is challenging to apply in tissues as it requires splitting cell populations, and assumes strong sister cell similarity, which may not always apply. A parallel set of synthetic biology approaches record information by continuously editing genomically integrated barcodes in a manner that allows reconstruction of lineage and signaling events^12^. However, these approaches rely on cis-regulatory elements engineered to respond to signals of interest and do not directly provide information about chromatin states across the genome. Finally, other approaches such as engineered RNA exporters^6^ or Live-seq^5^ have been developed to allow non-destructive measurements of RNA over time in the same cells, but do not directly provide information about chromatin states. Thus, the ability to recover chromatin state dynamics across the genome at multiple timepoints has remained a challenge.

Some chromatin profiling approaches store chromatin state information in live cells, potentially enabling dynamic measurements. In particular, while adenine DNA methylation rarely occurs in mammalian cells naturally, it can be implemented through recruitment of bacterial adenine methyltransferases to DNA in live cells^13^. More specifically, by fusing a protein of interest with bacterial DNA adenine methyltransferase (Dam), the DamID method recorded genome-wide DNA-protein interactions through profiles of adenine DNA methylation at GATC motifs^14^. MadID enhanced this approach, using a non-specific adenine methyltransferase (M.EcoGII) that does not require a specific consensus sequence to provide broader genome coverage^15^. The same work also showed that antibodies against methylated adenine (m6A) could be used in place of methylation-sensitive restriction enzymes (e.g. DpnI) to recognize adenine methylation (m6A) independent of sequence motifs. Finally, in the absence of active adenine methylation, deposited m6A modifications are partitioned to daughter cells^16^. Thus, m6A marks can be “written” in living cells, read out in fixed cells, and diluted, but not erased, during cell division. However, despite the potential of chromatin recording in live cells, these genome-wide approaches have primarily been applied to recording endpoint chromatin states by expressing fusion proteins just before harvesting cells. It has remained unclear whether they can also enable recording of past genome-wide chromatin states.

Recently, two chromatin recording approaches^17,18^ were introduced, both of which take advantage of bacterial methyltransferases to record information in DNA modifications. DCM-time machine (DCM-TM)^17^ uses the bacterial methyltransferase DCM fused to an RNA polymerase II subunit to label active genes and enhancers with cytosine methylation at specific sequence motifs. This approach revealed dynamic chromatin state changes during enterocyte differentiation in the mouse intestine. DCM methylation profiles were recorded in different time windows in different samples. However, this approach revealed only recorded chromatin profiles and could not also reveal endpoint chromatin profiles from the same cells.

In contrast, the Dam&ChIC approach^18^ enabled temporal chromatin profiling in single cells by measuring past chromatin profiles recorded by Dam together with present chromatin profiles by ChIC simultaneously. This approach resolved temporally ordered chromatin events such as nuclear lamina detachment and H3K27me3 accumulation during X chromosome inactivation. However, the DpnI restriction enzyme used in DamID cleaves fully methylated DNA much faster than hemi-methylated DNA containing the GATC motif^19^. As a result, its operation is typically limited to a time interval less than one cell cycle^16,18^. Thus, despite much work, a method that allows chromatin recording of states further into the past alongside endpoint chromatin measurements has been lacking.

**Figure 1|:**
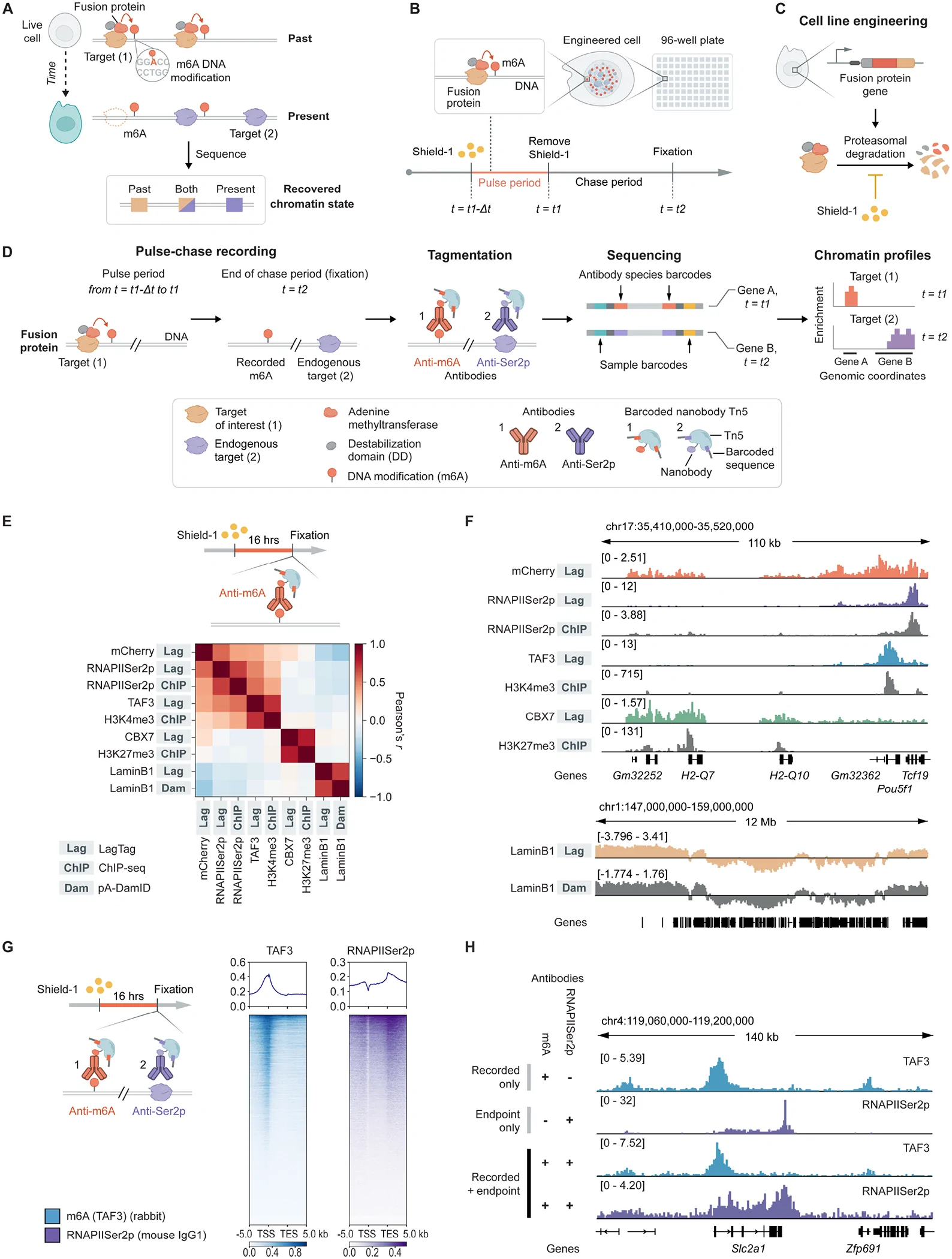
LagTag platform includes a temporal recorder and endpoint readout. **A**, The principle of recovering earlier chromatin states via recorded m6A DNA methylation alongside present states. **B**, **C**, Schematic illustration of the temporal recording of chromatin states. In engineered cell lines, the fusion protein between protein of interest and adenine methyltransferase controls the deposition of m6A DNA methylation based on its protein-DNA interactions during the pulse period (**B**). The small molecule Shield-1 can modulate the stability of the fusion protein (**C**) to achieve temporal chromatin recording. **D**, Schematic representation of the LagTag procedure, including chromatin state recording in live cells, recovery of recorded states via antibody-guided tagmentation in fixed cells, sequencing, and downstream analysis. Target of interest (1) during the recording and endogenous target (2) at the endpoint (e.g., RNAPIISer2p) can correspond to either the same or different molecular species. **E**, Schematic illustration of LagTag single-antibody assays (top). Correlation heatmaps of chromatin profiles for different targets as well as different profiling methods in mESCs (bottom). Correlations were calculated across 10-kb bins that overlapped with the union of peaks previously called from the ChIP-seq (H3K4me3 and H3K27me3) and CUT&Tag (RNAPIISer2p) datasets (n = 61,536 bins). **F**, Genome browser tracks of LagTag, ChIP-seq^20,37^, and pA-DamID^33^ samples in mESCs. Scale of LagTag profile was normalized to coverage per million aligned reads (top) or normalized to log2 ratio between Lamin B1 and mCherry profiles (bottom). The ChIP-seq profiles are input normalized and pA-DamID profiles are normalized by the Dam only control. n = 2 for TAF3 and CBX7, and n = 3 for mCherry, RNAPIISer2p, and Lamin B1 in LagTag experiments in **E** and **F**. **G**, Schematic illustration of LagTag multi-antibody assays (left). Signal enrichment plots for demultiplexed LagTag profiles for two antibodies around transcription start site (TSS) and transcription end site (TES) across all protein-coding genes in mESCs (right). **H**, Genome browser tracks of demultiplexed LagTag profiles for two antibodies along with individual LagTag and CUT&Tag profiles with single antibodies in mESCs. Normalization of the scale was performed similarly to **F** for LagTag and CUT&Tag. n = 2 for experiments in **G** and **H**.

**Figure 2|:**
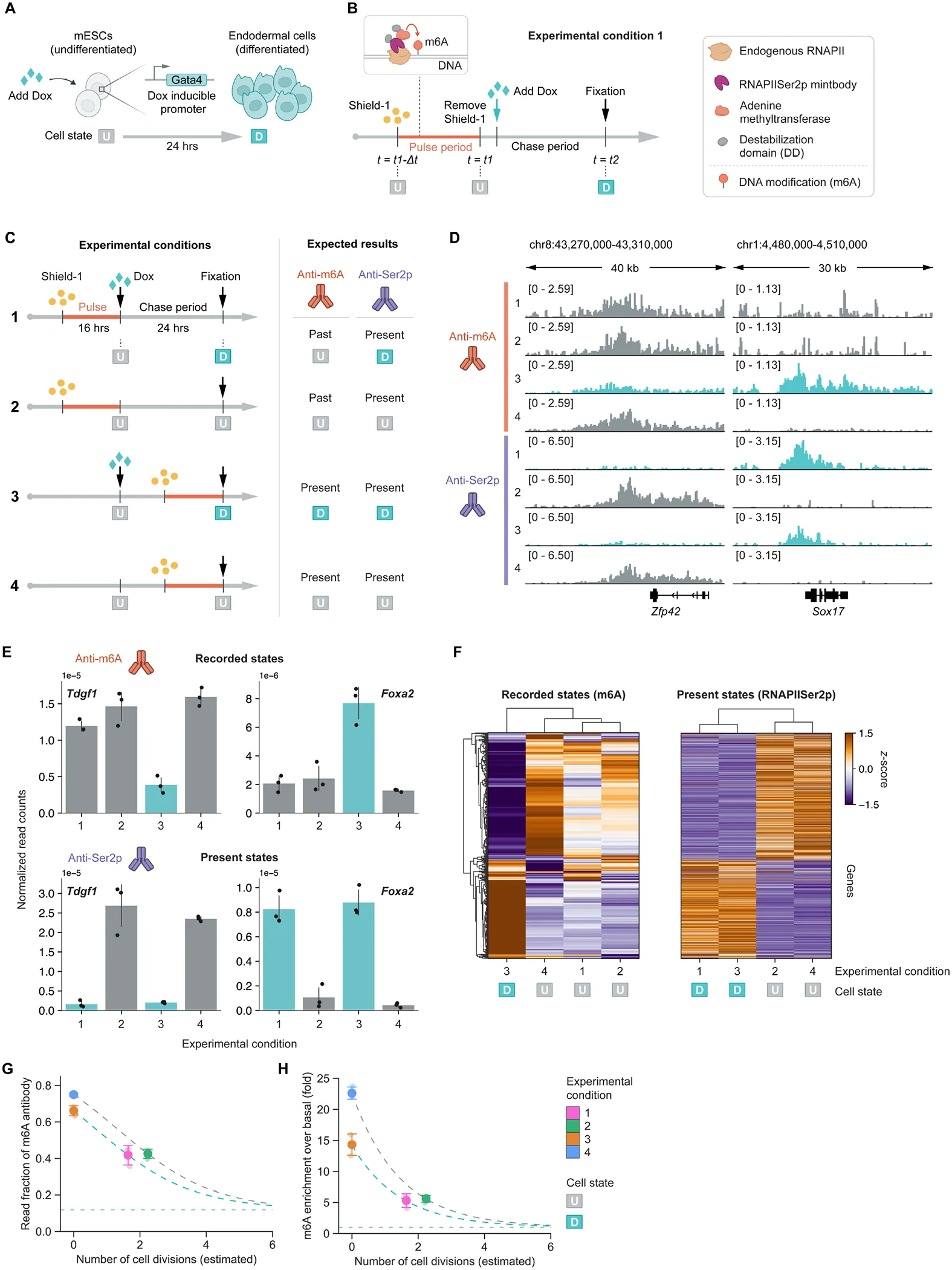
LagTag enables simultaneous chromatin profiling of two timepoints from the same samples. **A**, Schematic illustration of cellular differentiation in mESCs with inducible Gata4 system. **B**, LagTag allows temporal chromatin profiling by recording the previous chromatin states (undifferentiated cell state, U) and reading out after mESC differentiation (differentiated cell state, D). **C**, Additional schematic representation of LagTag experimental conditions with mESC differentiation along with expected results, corresponding to the recorded and endpoint chromatin states in each condition. The more detailed schematic for experimental condition 1 is depicted in **B**. **D**, Genome browser tracks of recorded and endpoint LagTag profiles across experimental conditions. **E**, Comparison of normalized read counts across experimental conditions. The color bars are colored by the expected cell states. The error bars represent standard deviations. **F**, Cluster maps of genes and conditions for recorded (left) and present (right) chromatin states using the differentially enriched genes with RNAPIISer2p (n = 933 genes) between undifferentiated and differentiated cell states, defined by separate CUT&Tag experiments (n = 3). Gene ordering in the right panel follows the clustering in the left panel. **G**, **H**, Read fraction of the m6A antibody (**G**) or m6A fold enrichment over basal (**H**) as a function of estimated cell divisions, modeled using a cell division dilution framework (see STAR Methods). Error bars represent mean ± standard deviations with individual replicate points overlaid. Results are shown separately for undifferentiated and differentiated cell states. Basal levels (dashed asymptotes) were determined from uninduced controls (conditions 5 and 6). Dashed curves show the predicted decay over cell divisions, derived from the measured basal and induced conditions (see Figure S9B). n = 3 for LagTag experiments in **D**-**H**.

Here we present LagTag, a technology that records chromatin states by pulse-labeling with m6A and recovers them alongside additional chromatin features by simultaneous readout at a later endpoint. m6A recording performed immediately before the endpoint captures the present chromatin states, similarly to conventional DamID approaches, whereas a subsequent chase period after recording enables retrospective profiling of past chromatin states across cell divisions. Technically, LagTag combines the temporal recording capabilities of bacterial methyltransferases in live cells with post-fixation tagmentation and sequencing readout. The use of tagmentation substantially reduces cell input requirements compared to immunoprecipitation-based methyltransferase methods such as MadID^15^. LagTag extends the temporal recording window of DamID to capture chromatin states separated by at least two cell cycles. LagTag effectively generates historical records of chromatin states that can be validated against established measurements such as those from ChIP-seq^20^ and CUT&Tag^21^. We first establish the technical foundation of LagTag (Figure 1) and then present proof-of-principle experiments demonstrating that temporal chromatin recording captures earlier chromatin signatures during mouse embryonic stem cell differentiation to endodermal fates^22^ (Figure 2).

## Results

### LagTag combines m6A recording with tagmentation-based endpoint readout

To develop m6A-based chromatin recording, we combined MadID^15^ and CUT&Tag^21^, which provide complementary capabilities. MadID deposits m6A modifications at specific genomic regions bound by the methyltransferase fusion to enable recording, but uses immunoprecipitation, which typically requires millions of cells, for sequencing readout. By contrast, CUT&Tag does not record earlier events, but enables chromatin profiling of multiple chromatin marks and proteins in fewer, or even single, cells at the endpoint by recruiting transposase to an antibody of interest bound at specific chromatin sites^21,23–26^. We reasoned that these approaches could be combined using m6A deposition to record chromatin states and antibody-guided tagmentation to read them out (Figure 1A). We term this combined approach LagTag, as it enables later (time-lagged) recovery of earlier chromatin profiles.

More specifically, for recording, we constructed fusions of the M.EcoGII adenine methyltransferase to chromatin binding proteins, including full-length proteins, their domains, and modification-specific intracellular antibodies (mintbodies)^27,28^ (Figure S1A), and minimized expression levels via a mutated Kozak sequence with reduced translation efficiency^29^. To sharply define the temporal recording window, we also incorporated a destabilization domain (DD) that can be inhibited by the Shield-1 small-molecule inducer^15,16,30^. With this modular system, adding and subsequently washing out Shield-1 leads to transient genome-wide adenine methylation at the specific sites where the protein of interest is localized during the labeling phase (Figure 1B–D). Recorded m6A marks can then be read out by sequencing via antibody-guided tagmentation using an m6A antibody^15^. Endpoint chromatin profiles can be read out using other CUT&Tag compatible antibodies. In particular, using a second, orthogonal antibody against elongating RNA polymerase II marked by Serine 2 phosphorylation (RNAPIISer2p) provides a readout of active expression at the final timepoint (Figure 1D). We note that the engineered fusion protein used to record the past state can be the same as or different from the endogenous target detected in the present state, as they are independent.

### LagTag reproduces known chromatin profiles

To validate LagTag, we first asked whether recorded m6A profiles aligned with publicly available datasets in mouse embryonic stem cells (mESCs) and human HCT116 cells. To record, we fused the non-specific adenine methyltransferase M.EcoGII^15^ with different full-length proteins, protein domains, and mintbodies (Figure 1B, Figure S1A), then generated stable cell lines expressing each construct individually (Figure 1C). First, to target endogenous elongating RNAPII, we fused M.EcoGII and DD with a mintbody targeting RNAPIISer2p (Ser2p-mintbody)^31^. Second, to profile histone modifications, we fused M.EcoGII and DD with well-characterized chromatin reader domains from TAF3 and CBX7, which recognize H3K4me3 and H3K27me3, respectively^27,28^. Third, to mark repressive lamina-associated domains (LADs), we fused M.EcoGII and DD to full-length Lamin B1, a protein that binds to the nuclear lamina and is widely used for DamID and related approaches^15,16,32^. Finally, as a negative control, we also fused M.EcoGII and DD to a non-specific mCherry fluorescent protein. Together, these constructs provided tools for inducible temporal control of m6A recording of chromatin states.

Using these constructs, we induced recording for 16 hours in mESCs and HCT116 cells grown in standard media in individual wells of 96-well plates containing ~10,000–30,000 cells (see STAR Methods). We then fixed cells with 1% formaldehyde for 5 minutes, performed endpoint assays, and compared the results with existing data sets (Figure 1E, F, Figure S1–4, Table S1, 2). In mESCs, the Ser2p-mintbody, TAF3 and CBX7 reader domains, Lamin B1, and mCherry (negative control) all exhibited distinct genomic enrichment patterns, as expected (Figure 1E, F, Figure S2). Specifically, mCherry, Ser2p-mintbody, and TAF3 were associated with active chromatin, CBX7 was associated with facultative heterochromatin, and Lamin B1 was associated with repressive LADs. Further, these profiles agreed with independent chromatin profiling measurements using ChIP-seq^20^, CUT&Tag^21^, and pA-DamID^33^. For example, in mESCs, the recorded TAF3 (H3K4me3 reader domain) profiles were similar to those obtained by ChIP-seq and CUT&Tag (Pearson’s correlation coefficients of 0.76 and 0.87, respectively for H3K4me3, Figure 1F, Figure S2C, E, F). The LagTag profiles also showed specific enrichment patterns at transcription start sites (TSSs) for TAF3, transcription end sites (TESs) for RNAPIISer2p^34^, or called H3K27me3 peaks for CBX7 (Figure S3A). Similarly, background-normalized LagTag and pA-DamID signals were strongly correlated for Lamin B1 (Figure 1F, Figure S2D, F, Pearson correlation coefficient of 0.91). We note that the modified CUT&Tag protocol used here efficiently recovered tagmented fragments from fixed cells (Figure S1B) and enabled mapping of Lamin B1 (Figures S2D–F, 3B), which, until recently, was considered difficult to profile by tagmentation^35^. Furthermore, we observed good agreement between m6A recorded profiles and independent measurements for RNAPIISer2p and Lamin B1 in human HCT116 cells (Figure S4). These data confirm that LagTag, by using m6A marks and an m6A antibody, replicates established measurements that use restriction enzymes or target-specific antibodies (Figure 1E, F, Figures S2–4).

One potential limitation of the use of M.EcoGII is its ability to methylate RNA substrates^36^. To identify alternative enzymes that would potentially exhibit reduced RNA methylation, we scanned a large number of M.EcoGII variants (Figure S5A, B). LagTag also worked with one of these variants, EspM, a non-specific adenine methyltransferase (Figure S5C, D). EspM exhibits a reduced methylation rate for RNA substrates in vitro, while preserving DNA methylation (Figure S5B). While M.EcoGII fusion proteins were used for the rest of this study, this result suggests that LagTag can be extended to other adenine methyltransferase variants with distinct properties. Taken together, these results demonstrate that LagTag can accurately profile various active and repressive chromatin regions in mouse and human cell lines at relatively low (~10,000) numbers of cells.

### LagTag enables simultaneous profiling of m6A-recorded and endpoint chromatin signatures

To obtain chromatin profiles of the same cells at different timepoints, it is necessary to simultaneously read out signatures corresponding to both past and present states (Figure 1A, D). To test this ability, we adapted multifactorial CUT&Tag approaches previously used to map multiple chromatin proteins and modifications in single assays^23–26^. Specifically, we adapted nanobody-based multifactorial CUT&Tag, which employs uniquely barcoded nanobody-transposases (nb-Tn5) to orthogonally bind to and distinguish the different antibody species (i.e. rabbit and mouse IgG1)^25,26^ (Figure S6A).

Using the optimized 96-well format described above, multifactorial CUT&Tag worked across multiple targets, including RNAPIISer2p, H3K4me3, and H3K27me3 in fixed cells (Figure S6A, B). For example, consistent with previous studies, the profiles of RNAPIISer2p and H3K27me3 showed minimal overlap in mESCs^23^ (Figure S6C–E). This indicates there is minimal cross talk between barcodes targeting different molecular species.

Next, we performed LagTag assays using two antibodies (e.g., m6A and RNAPIISer2p) and the two barcoded nb-Tn5 species together on the same cell population (Figure 1G, H, Figure S7A–F). The combined assays produced similar chromatin profiles as the individual LagTag and CUT&Tag assays for TAF3 and RNAPIISer2p pairs (Pearson correlation coefficients of 0.98 and 0.81) as well as Lamin B1 and RNAPIISer2p pairs (Pearson correlation coefficients of 0.96 and 0.81) (Figure S7C–E). We also confirmed the expected differential enrichment of TAF3 and RNAPIISer2p at TSS and TES, respectively, as well as Lamin B1 enrichment and RNAPIISer2p depletion at LADs (Figure 1G, H, Figure S7F). LagTag requires denaturation of chromatin by NaOH in order to enable the m6A antibody to access DNA modifications^15^. This treatment preserved CUT&Tag profiles for RNAPIISer2p at the TES and gene body, enabling the identification of genes with high levels of RNAPIISer2p at their gene bodies (Figure S7G, H). However, it also depleted the previously observed RNAPIISer2p enrichment at TSS^37^ for unknown reasons (Figure S7G). Together, these results demonstrate simultaneous mapping of m6A-recorded and endpoint chromatin profiles, a critical requirement for LagTag.

### LagTag resolves distinct past and present chromatin states during cellular differentiation

Having established the ability of LagTag to recover recorded and endpoint chromatin profiles, we next asked whether we could use LagTag to analyze dynamic chromatin changes. Cellular differentiation provides an ideal use case, since the influence of earlier chromatin states on subsequent cell state changes is of broad interest but difficult to infer from static snapshots. As a model system, we focused on endodermal differentiation, a key step in many regenerative medicine approaches^38^. We used an mESC line with a doxycycline (Dox)-inducible Gata4 gene, whose expression is sufficient to induce endodermal fate^22^. To enable recording, we stably incorporated a Shield-1-inducible M.EcoGII-Ser2p-mintbody in this cell line. The resulting system produced endodermal differentiation in cell populations in response to 24 hours of Dox exposure in both the parental and M.EcoGII-Ser2p-mintbody engineered cell lines (Figure S8). Further, in response to Dox, chromatin profiles of endodermal and pluripotent marker genes switched markedly, with minimal signal remaining from the prior undifferentiated state, suggesting homogeneous changes across the population (Figure S8C).

To ensure that recording could be limited to desired time windows, we fused destabilization domains (DDs) to both N- and C- termini of M.EcoGII-Ser2p-mintbody (following a strategy used in ref.^39^). The dual DDs suppressed basal activity, as shown by the increased fold change of m6A marks upon addition of Shield-1 compared to the construct only with the N-terminus DD (Figure S1F–H).

Using this engineered cell line, we asked whether it is possible to simultaneously read out the pre- and post-differentiation chromatin profiles of the same cell population. We performed pulse-chase m6A recording by inducing M.EcoGII-Ser2p-mintbody in undifferentiated cells for 16 hours to record chromatin state (pulse) (Figure 2A, B, Figure S9, 10). After washing out Shield-1 for a few hours to halt recording, we induced Gata4 for 24 hours to stimulate endodermal differentiation (chase). During this time, cells underwent approximately two cell divisions (2.1 ± 0.3 and 1.9 ± 0.2 divisions, mean ± standard deviation, for parental and engineered cells, respectively) (Figure S10A, B). This is consistent with cell cycle times of 13.25 hours (undifferentiated) and 10.5 hours (early-differentiated) reported by live-cell imaging^40,41^.

Following the pulse-chase experiment, we examined the chromatin profiles of pluripotency and differentiation-associated genes (Figure 2B, C, condition 1, Figure S9). Recorded m6A marks were enriched around the TES of Zfp42, a pluripotency factor expressed in undifferentiated mESCs^42^, reflecting the higher expression of this gene in the past state (Figure 2C, D, condition 1). In the same sample at the endpoint, RNAPIISer2p was enriched around the TES of Sox17, a marker gene for endodermal differentiation^22^ (Figure 2C, D, condition 1). Conversely, we did not observe enrichment of Sox17 in the recorded m6A profile nor enrichment of Zfp42 in the endpoint RNAPIISer2p profile. These recorded and endpoint chromatin profiles reflect the expected changes of RNAPIISer2p occupancy during differentiation based on our earlier analysis (Figure S8).

We also quantified signal enrichment over gene bodies. We confirmed RNAPIISer2p enrichment in the undifferentiated marker gene Tdgf1 in the recorded state and the differentiated marker gene Foxa2 in the endpoint state (Figure 2E, condition 1, Figure S9E), as expected^22,43^. Finally, we analyzed 933 genes that were differentially enriched with RNAPIISer2p after differentiation (Figures S8B, S9F). The recorded m6A signatures from experimental condition 1 clustered together with those from other undifferentiated conditions obtained at either early or endpoint timepoints (Figure 2F, left). Similarly, the endpoint RNAPIISer2p signatures from condition 1 clustered together with the other differentiated condition (Figure 2F, right), as expected. Together, these results demonstrate that LagTag can recover undifferentiated (past) and differentiated (present) chromatin profiles from the same sample, exhibiting expected differences in specific marker genes and genome-wide profiles.

To confirm these results arose from biological processes rather than technical artifacts, we included several control conditions (Figure 2C, Figure S9A–D). First, we omitted Dox to eliminate differentiation and maintain cells in the undifferentiated state (Figure 2C, condition 2). When we performed the LagTag assay in this condition, we observed similar profiles between the m6A and RNAPIISer2p marks on marker genes (Figure 2D, E). Second, we recorded chromatin states at the endpoint by delaying induction of the M.EcoGII fusion until the last 16 hours before fixing the cells, with or without Dox (Figure 2C, conditions 3 and 4). Again, the m6A and RNAPIISer2p profiles corresponding to the same cell state displayed similar trends on marker genes (Figure 2D, E), indicating that the assays are largely consistent with one another regardless of cell state and timing of recording. We also extended this comparison to the fold-change across all coding genes during differentiation, confirming that LagTag shows a positive correlation between m6A and RNAPIISer2p (Pearson correlation coefficient of 0.71) as well as in comparison with conventional RNAPIISer2p CUT&Tag (0.65 and 0.76 for m6A and RNAPIISer2p, respectively) (Figure S9G). In contrast, changes of some of the shorter non-coding genes (e.g., miRNA) were not detected by LagTag (Figure S9H), which represents a potential limitation of the protocol. Third, we included additional negative controls with no expression of the M.EcoGII fusion protein, also with or without differentiation (Figure S9A, conditions 5 and 6). When the methyltransferase was not induced (no recording), most sequencing reads were from the endpoint assay of RNAPIISer2p, indicating that LagTag has a low background of basal enzymatic activity (Figure S9B). Furthermore, we confirmed that LagTag recording minimally perturbs cellular physiology during differentiation, as measured by cell viability, cell cycling, and instantaneous transcriptional profiles mapped by RNAPIISer2p enrichment in comparison with parental cells (Figure S10).

### LagTag resolves past chromatin states within two cell divisions

Finally, we estimated the m6A signal decay as a function of cell divisions (Figure 2G, H) by leveraging the read count ratio between m6A and RNAPIISer2p across conditions in combination with a cell division dilution model (see STAR Methods). Based on this analysis, we estimate approximately two cell divisions elapsed for undifferentiated and differentiated cells (2.2 ± 0.1 and 1.7 ± 0.3 with mean ± standard deviation, respectively) during the 24-hour chase period, consistent with the direct results from the cell division assay (2.0 ± 0.1 and 1.9 ± 0.2 divisions, respectively) (Figure S10B). This model further defined an effective window of approximately two or three cell divisions, within which m6A signal enrichment remains robust (e.g., 14.2 and 4.3 fold m6A signal enrichment over basal in differentiated cells with no chase and 2-division chase, respectively), before progressively approaching background levels after approximately six cell divisions (Figure 2G, H). These results establish that LagTag can retrospectively profile chromatin states across at least two cell divisions in proliferating cells.

Taken together, these results demonstrate how LagTag enables genome-wide analysis of past and endpoint chromatin states within the same cell population across cell cycles.

## Discussion

Recovering historical events in cells—especially chromatin states—is essential for understanding how cells change and make fate decisions over time. Compared to previous approaches, LagTag introduces several innovations. First, it extends the duration over which recorded information can be recovered. With conventional DamID, a single round of DNA replication can limit signal recovery, due to the faster digestion of fully methylated DNA compared to hemimethylated by DpnI^19^. Here, by contrast, the use of the m6A antibody inherently allows detection of hemi-methylated strands, which overcomes this limitation, and allows m6A to be used for genome-wide chromatin recording. Second, LagTag enables recovery of endpoint states alongside earlier recorded states in the same cell population. This is made possible by combining m6A for recovery of past information with another antibody (e.g. RNAPIISer2p) capturing the endpoint information. Third, LagTag makes it easier to profile diverse conditions by decreasing the number of cells required for analysis compared to MadID^15^. Where MadID requires millions of input cells, LagTag requires only ~10^4^ cells and can therefore be performed in individual wells in 96-well plates. Thus, LagTag allows analysis of two timepoints in a high-throughput fashion with relatively low cell input.

LagTag should address previously intractable biological problems. For example, the origins of heterogeneity in cellular responses are often mysterious^44^. Different cells can exhibit dramatically different responses to signals or drugs, or make distinct cell fate choices in the same environment. The ability to recover earlier chromatin states, even from bulk populations, will help identify signatures that predispose cells to different outcomes or future trajectories in healthy and disease contexts. This is conceptually analogous to two-timepoint mRNA profiling, which has recently enabled transcriptional recording at the population level and identified molecular signatures of drug-naïve persister cells in a human cancer line^45^. Another set of questions pertains to dynamic changes in chromatin state in the central nervous system. Previous work has implicated chromatin changes during memory formation and recall in neurons in the hippocampus^46^. In addition, changes in the subnuclear localization of DNA loci are known to functionally affect transcriptional activation and repression^47^. LagTag could be used to record and recover earlier subnuclear localization states to explore the dynamics and functional impact of nuclear compartment switching during development and aging. While LagTag and other two-timepoint approaches alone do not establish direct causality between earlier chromatin states and subsequent cell fate transitions, they can reveal candidate molecular signatures and temporal ordering of events that are typically inaccessible from static, single timepoint measurements^8,18^.

LagTag could be extended in several ways. First, because CUT&Tag and related methods have been shown to operate at the single cell level^21,23–26^, it should be possible to similarly scale down LagTag to access single-cell histories. Single-cell LagTag would enable retrospective identification of chromatin signatures that bias cell state transitions. More specifically, under conditions of heterogeneous differentiation, one could group cells by their endpoint chromatin states, and then identify common, earlier, fate-determining chromatin signatures. Second, LagTag could be combined with imaging-based readout technologies to measure spatial proximities^48,49^ or molecular interactions^50^ between DNA loci and antibodies, potentially enabling spatiotemporal single-cell chromatin profiling, including past cell state information, directly within tissues.

LagTag has several limitations in its current form. First, the current study was performed on bulk populations, and a single-cell version remains to be developed. Second, m6A marks do not propagate and therefore dilute out over multiple rounds of cell division. Synthetic engineering of m6A replication mechanisms or the use of methylation dependent Dam mutants could help to address this issue^51,52^. Third, the current 16-hour m6A labeling window may exceed the cell cycle time of some fast-dividing cells, masking biological changes shorter than the labeling window. Fourth, non-specific adenine methyltransferases such as M.EcoGII can methylate RNA as well as DNA, potentially leading to cellular toxicity. As we showed, this issue could be overcome by further engineering or evolving the enzymatic properties of M.EcoGII^36^ to eliminate RNA methyltransferase activity while preserving DNA methylation. Despite these caveats, the temporal chromatin profiling approach we present here provides a versatile framework for resolving previously hidden cell state dynamics.

After posting the preprint describing this work, two manuscripts were posted, describing related approaches^53,54^. We provide a summary comparison of recent temporal chromatin profiling methods including these studies (Table S3). Collectively, this work should provide a foundation for applying chromatin recording to diverse challenges.

## Resource Availability

### Lead Contact:

Further information and requests for resources and reagents should be directed to and will be fulfilled by the Lead Contact, Michael Elowitz (melowitz@caltech.edu).

### Materials Availability:

Plasmids and cell lines generated in this study are available from the lead contact upon request.

### Data and Code Availability:

- Source data: Sequencing data have been deposited at NCBI GEO and are publicly available as of the date of publication. Accession number is listed in the key resources table. Additional processed data have been deposited at CaltechDATA and are publicly available as of the date of publication. DOI is listed in the key resources table. This paper analyzes existing, publicly available data. These accession numbers for the datasets are listed in the key resources table.
- Code: All original code has been deposited at CaltechDATA and is publicly available as of the date of publication. DOI is listed in the key resources table.
- Any additional information required to reanalyze the data reported in this paper is available from the lead contact upon request.

## STAR Methods

### Experimental model and subject details

#### Cell lines

E14 mouse embryonic stem cells (E14Tg2a.4) were maintained on 0.1% gelatin (Sigma-Aldrich G1393) coated plates under the serum/LIF condition in DMEM (Gibco 11960044) supplemented with 15% ES-grade fetal bovine serum (FBS, Gibco 16141061), 1,000 units/mL leukemia inhibitory factor (LIF) (Sigma-Aldrich ESG1106), 1× non-essential amino acids (Gibco 11140050), 1 mM sodium pyruvate (Gibco 11360070), 55 μM 2-mercaptoethanol (Gibco 21985023), and 1× Penicillin-Streptomycin-Glutamine (Gibco 10378016). mESC-Gata4 cells^22^ were kindly provided by Magdalena Zernicka-Goetz and maintained on 0.1% gelatin coated plates under the serum/LIF/2i condition by supplementing the serum/LIF medium with 1 μM PD0325901 (Sigma-Aldrich 444966) and 3 μM CHIR99021 (Sigma-Aldrich SML1046). The mESC differentiation to endodermal cells was performed with 1 μg/mL Dox induction and LIF withdrawal for 24 hours. The human HCT116 (ATCC CCL-247) cells were maintained in McCoy’s 5A (modified) medium (Gibco 16600082) supplemented with 10% FBS (Avantor 97068–085) and 1× Penicillin-Streptomycin (Gibco 15140122). The cell lines were not routinely tested for mycoplasma contamination. All cell lines were cultured at 37°C with 5% CO2.

### Method details

#### LagTag construction design

Plasmids were constructed in a PiggyBac vector (System Biosciences) with a puromycin resistance gene using standard cloning methods. The adenine methyltransferase fusion genes are expressed under the PGK promoter and mutated Kozak sequence, TGATAT, for lower translation efficiency^29^. The DD-linker-M.EcoGII was cloned from pRetroX-PTuner DD-linker-M.EcoGII (Addgene 122082) and DD-linker-M.EcoGII-LaminB1 (LMNB1) was cloned from pRetroX-PTuner DD-linker-M.EcoGII-v5-LaminB1 (Addgene 122083)^15^. The Ser2p-mintbody^31,71^, TAF3 (three chromo domains)^28^, CBX7 (two chromo domains)^27^, and EspM were cloned from gBlocks (Integrated DNA technologies) to make the similar constructs. The DD was further amplified by PCR and cloned to make dual DD constructs.

#### Generation of stable LagTag cell lines

To make stable cell lines with adenine methyltransferase fusion constructs, the cells were transfected with 50 μg of the PiggyBack vectors on a 24-well plate using FuGENE HD transfection reagent (Promega E2311). The polyclonal cells were selected using 1 μg/mL puromycin and single clones for mESCs were manually isolated for some of the experiments. The adenine methyltransferase fusion proteins were stabilized with 1 μM Shield-1 (Takara Bio 632189) for 16 hours.

#### Exploration of M.EcoGII homologs

Twenty-six M.EcoGII homologs between anc291 and anc289 nodes were selected from the maximal likelihood tree constructed previously^36^. Two unaligned sequences were excluded from the multiple sequence alignment (MSA). 19 of 24 sequences were selected based on conservation of residue Q179, which is a key residue for enhanced RNA methylation activity. One unaligned node was excluded using a sequence similarity network (SSN) generated from the sequences. Sequence similarity was calculated by Ident and Sim^70^ and similarity The amino acid set was used for similarity calculation: [GAVLI, FYW, CM, ST, KRH, DENQ, P]. Finally, 6 sequences were selected by sequence similarity less than 90% to two ancestral M.EcoGII variants: Anc291 and Anc289.

A sequence encoding a C-terminal His6 tag with a linker sequence (N-GGAAALGHHHHHH-C) was added to each candidate gene, and the resulting DNA sequences were synthesized and cloned into the pET-28a (+) plasmid using seamless cloning method. In-frame cloning into the pET-28a (+) vector was confirmed by colony PCR and DNA sequencing.

Plasmids containing candidates were transformed into E. coli strain SHuffle T7 (C3026J, NEB) and plated on LB agar containing 30 μg/mL kanamycin and 2% glucose, incubating at 37°C overnight. Colonies of CspM were inoculated into 10 mL of LB media with 30 μg/ml kanamycin and 2% glucose and incubated at 37°C with shaking at 220 rpm overnight. The preculture was transferred into 500 mL of LB media with 30 μg/mL kanamycin and 1% glucose, and the E. coli cells were grown at 37°C with shaking at 220 rpm until the OD600 reached 0.5–0.8. Colonies of EspM, PvaM, or KpnNIH were directly inoculated into 750 mL LB media with 30 μg/mL kanamycin and 1% glucose without preculture, and the E. coli cells were grown at 37°C with shaking at 220 rpm until the OD600 reached 0.5–0.8. Protein expression of DNA selective MTase candidates was induced by 1 mM isopropyl β-d-1-thiogalactopyranoside (IPTG) (EI07011, Biosynth) for 18 hours at 18°C. Cells were harvested and stored at −20°C before protein purification.

Cells were resuspended with lysis buffer (20 mM HEPES (pH 7.0), 250 mM NaCl, and 10 mM imidazole) and lysed by sonication. The lysis supernatant was obtained by centrifugation at 10,000 × g, 4°C for 30 min. M.EcoGII homologs were purified by affinity chromatography in an open column, eluting with 20 mM HEPES (pH 7.0), 250 mM NaCl and 100 mM imidazole at room temperature. Each fraction was analyzed by SDS–PAGE. Fractions enriched in the target protein were pooled, filtered through a 0.22 μm filter and concentrated in an Amicon Ultra 30 kDa MWCO centrifugal concentration device (UFC9030, Merck) with working buffer (20 mM HEPES (pH 7.0), 250 mM NaCl). The concentrated protein samples (>1 mg/mL protein and <1 mM imidazole) were mixed with glycerol to reach 10% (v/v) concentration, quickly frozen by liquid nitrogen and stored at −80°C.

#### In vitro methyltransferase activity measurement

The DNA substrate encoding M.EcoGII (3569 bp, containing 1819 adenine bases) was amplified by PCR and purified using a NucleoSpin Gel and PCR Clean-up Kit (740609, MACHEREY-NAGEL) with Milli-Q water. The RNA substrate encoding the firefly luciferase gene (1872 nt, 544 adenine bases) was prepared by in vitro transcription using HiScribe T7 High Yield RNA Synthesis Kit (E2040S, NEB) with 1 unit RNase Inhibitor and purified in ribonuclease (RNase)-free water using an RNA Clean & Concentrator-5 kit (R1015, Zymo Research). In vitro methylation experiments were performed for 10 minutes (DNA) or 1 hour (RNA) at 37°C in 8 μL reaction mixtures containing 1× CutSmart buffer with 20 nM DNA or 50 nM RNA with 1 unit RNase Inhibitor (M0314S, NEB), 100 nM methyltransferase and 20 μM SAM. The methylation reaction in the 8 μL reaction was quenched by heating at 80°C for 1 min at each timepoint. The methyltransferase reaction product S-adenosylhomocysteine was quantified using MTase-Glo kit (V7601, Promega) following the manufacturer’s protocol^72^. To normalize the S-adenosyl homocysteine (SAH) production rate in DNA/RNA methylation, a calibration curve for SAH was constructed in two independent experiments using from 0 to 5 μM SAH according to the manufacturer’s protocol of the MTase-Glo kit. The signal was detected using a Tecan Spark microplate reader (Tecan Japan Co., Ltd).

#### Nanobody-Tn5 transposome preparation

The pTXB1-nbOcIgG-Tn5 (Addgene 184285) and pTXB1-nbMmIgG1-Tn5 (Addgene 184287) plasmids^25^ were purchased from Addgene. The nb-Tn5 transposase production was performed as previously described with modifications. The plasmid was transformed into ArcticExpress (DE3) Competent Cells (Agilent Technologies 230192), and 1 liter of terrific broth culture was grown at 37 °C until the OD600 reached 0.6. The nb-Tn5 expression was then induced with 0.25 mM isopropyl-ß-D-thiogalactopyranoside (IPTG) at 22 °C for 18 hours. The cells were then pelleted and stored at −80 °C until use. For protein purification, the cells were lysed by sonication in 100 mL of HEGX buffer, consisting of 20 mM HEPES-KOH pH 7.5, 1 M NaCl, 1 mM EDTA, 10% glycerol, 0.2% Triton X-100, and a protease inhibitor cocktail (Roche 04693132001). The lysate was then pelleted at 30,000g at 4 °C for 20 minutes. The supernatant was transferred to a new tube, and 10% polyethylenimine (Sigma-Aldrich P3143) was added to the bacterial extract, followed by gentle mixing and centrifugation at 30,000g at 4 °C for 30 minutes. The supernatant was then loaded on an Econo-Column column (Bio-Rad Laboratories 7371022) filled with 10 mL of chitin resin (NEB S6651L). The column was washed with 50 mL of HEGX buffer, and then incubated with 15 mL of HEGX buffer supplemented with 100 mM DTT at 4 °C for 48 hours to cleave the nb-Tn5 from the intein tag. The nb-Tn5 was eluted directly into one 10 kDa MWCO Amicon Ultra Centrifugal Filter (Millipore UFC9010) by the addition of 10 mL of HEGX buffer. The protein was spin concentrated in the Amicon Ultra Centrifugal Filter using 15 mL of 2× dialysis buffer, consisting of 100 mM HEPES-KOH pH 7.2, 0.2 M NaCl, 0.2 mM EDTA, 2 mM DTT, 20% glycerol, and concentrated to 1 mL by centrifugation at 5,000g. The protein concentrate was transferred to a new tube and mixed to make a final concentration of 50% glycerol. The nb-Tn5 aliquots were stored at −80 °C.

The nb-Tn5 adapter transposomes were assembled as previously described^26^. Briefly, 100 μM equimolar mixture of two adaptor oligonucleotides (Table S2) in an annealing buffer, consisting of 10 mM Tris-HCl pH 8.0 (Invitrogen 15568025), 50 mM NaCl (Invitrogen AM9759), 1 mM EDTA pH 8.0 (Invitrogen AM9260G) in DNase/RNase-free distilled water (Invitrogen 1097701), was denatured on a thermocycler for 5 minutes at 95°C and cooled down at room temperature for more than 45 minutes for annealing. The 100 μM equimolar mixtures (50 μL of each) were mixed with 500 μL of 6.8 μM nb-Tn5 in the 2× dialysis buffer and incubated for 1 hour at room temperature. The transposomes were mixed with 100% glycerol to make a final concentration of 50% glycerol and stored at −20 °C until use.

#### On-plate LagTag and CUT&Tag

LagTag and CUT&Tag experiments were performed using adherent cells directly fixed on the 96-well plate. The conventional CUT&Tag protocol^73^ was optimized for this implementation based on the recent chromatin profiling technologies^74–76^ and by incorporating the nanobody-based transposome^25,26^. E14 and mESC-Gata4 cells (6,000–12,000 cells) were seeded on human laminin (BioLamina LN511) coated 96-well plates, and HCT116 cells (10,000 cells) were seeded on collagen (Sigma-Aldrich C8919) coated ibiTreat 96-well plates (ibidi 89606). Cells were washed with 100 μL of DPBS (Gibco 14190144) once and fixed with 50 μL of freshly-prepared 1% formaldehyde (Thermo Scientific 28908) buffer in 1× PBS (Invitrogen AM9624) at room temperature for 5 minutes. The fixed cells were washed twice with 100 μL of DPBS and used on the same day. The DNase/RNase-free distilled water was used throughout the experiments. The cells were permeabilized with 50 μL of 0.5% Triton-X (Sigma-Aldrich 93443) in 1× PBS at room temperature for 20 minutes and washed once with 50 μL of 1× PBS at room temperature. For LagTag, to remove endogenous m6A RNA modifications, cells were incubated with 50 μL of 5% RNaseA/T1 Mix (Thermo Fisher EN0551) in 1× PBS at 37°C for 1 hour. Then cells were denatured^15^ with 30 μL of a denaturation buffer, consisting of 1.5 M NaCl (Invitrogen AM9759) and 500 mM NaOH (Teknova H0224), at room temperature for 30 minutes, followed by three washes with 50 μL of a neutralization buffer, consisting of 500 mM Tris-HCl pH 7.5 (Invitrogen 15567027) and 2.5 M NaCl, at room temperature. For LagTag with multi-antibody samples, cells were denatured with a milder condition of 150 mM NaCl and 50 mM NaOH at room temperature for 5 minutes, followed by three washes with a neutralization buffer (50 mM Tris-HCl pH 7.5 and 250 mM NaCl). The samples were further washed with 50 μL of 1× PBS at room temperature three times. Those RNase and denaturation steps were required specifically for LagTag and were omitted in CUT&Tag experiments unless otherwise specified. The samples were then washed once with 1× PBS and blocked with 20 μL of a blocking solution consisting of 1× PBS, 10 mg/mL UltraPure BSA (Invitrogen AM2616), 0.05% Triton-X at room temperature for 5–15 minutes. Then the cells were incubated with 30 μL of 100-fold diluted primary antibodies in the blocking solution at 4°C overnight. Primary antibodies used in this study are listed in Table S1. On the following day, the samples were washed twice with 50 μL of wash 300 buffer, consisting of 20 mM HEPES pH 7.4 (Teknova H1030), 300 mM NaCl, and 0.05% Triton-X, and then incubated with 30 μL of wash 300 uL buffer supplemented with 1.5–3 μL of nb-Tn5 transposome (in-house) and 0.1% BSA at room temperature for 1 hour with a gentle shaking of 300 rpm. For multi-antibody LagTag or CUT&Tag experiments with barcoded adapters, cells were incubated at 4°C overnight in 30 μL of wash 300 buffer supplemented with 2 μL of each barcoded nb-Tn5 transposome and 0.1% BSA. The samples were then washed twice with 50 μL of wash 300 buffer. The samples were then incubated with 50 μL of tagmentation buffer (10 mM MgCl_2_ (Invitrogen AM9530G) and 0.1% BSA in the wash 300 buffer) at 37°C for 1 hour. We note that the plates were sealed with adhesive PCR plate sealing films (Bio-Rad Laboratories MSB1001B) to prevent evaporation and placed in the 37°C incubator without stacking multiple plates to allow homogeneous heating on the plate surface. Then the samples were washed once with 50 μL of a TAPS buffer, consisting of 10 mM TAPS buffer pH 8.5 (Boston BioProducts BB-2375) and 0.2 mM EDTA. Then 20 μL of SDS release buffer, consisting of 10 mM TAPS buffer pH 8.5, 0.2% SDS (Invitrogen 24730020), and 5% thermolabile proteinase K (NEB P8111S), was added to each well, followed by an incubation at 37°C for 30 minutes and then at 62°C for 30 minutes. Similarly to the tagmentation step, plates were covered by the seal and each plate was placed on the surface of the incubator. The use of thermolabile proteinase K in the release buffer was recently introduced for heavily crosslinked formalin-fixed paraffin-embedded samples^76^, but we noticed it is applicable to recover longer fragments from crosslinked samples on the plate by 1% formaldehyde. The SDS release buffer was then mixed with 12 μL of 10% Triton-X for neutralization and used as a PCR input. The sequencing libraries were then prepared by adding 21 μL of the input, 2 μL of i5 and i7 primers (Integrated DNA technologies, 10 μM stock each) (Table S2), and 25 μL of NEBNext High-Fidelity 2X PCR Master Mix (NEB M0541L) to each PCR tube, followed by PCR amplifications: 5 min at 58°C, 5 min at 72°C, and 45 sec at 98°C followed by 13–15 cycles of 15 sec at 98°C and 10 sec at 60°C, with a final extension at 72°C for 1 min and a hold at 4°C. The amplified libraries were purified using 1.3× magnetic beads (Omega Bio-Tek M1378) according to the manufacturer’s protocol.

#### Sequencing

After the pooled libraries were purified by E-Gel EX Agarose Gels, 2% (Invitrogen G402022) and spin column (Zymo D4001, D4014), the samples were quantified using the Qubit dsDNA High Sensitivity Assay (Invitrogen Q32854) and the Agilent High Sensitivity D1000 DNA ScreenTape Assay (Agilent Technologies 5067–5603, 5067–5584). Then the paired-end sequencing of the libraries was performed on the AVITI using the AVITI 2×150 Sequencing Kit Cloudbreak FS (Element Biosciences 860–00011, 860–00013) with Cloudbreak FS PhiX Control, 3rd Party (Element Biosciences 830–00023) according to the manufacturer’s protocol with a 2% PhiX spike-in. For the adapter-barcoded samples, custom primers^23^ (Integrated DNA technologies) were used for Read 1, Read 2, and Index 1 (Table S2) with Custom Primer Set Cloudbreak Freestyle (Element Biosciences 820–00025) and libraries were mixed with a 30% PhiX spike-in.

#### Quantification of m6A deposition by qPCR

Tagmentation efficiency, reflecting the extent of m6A deposition, was assessed by qPCR as previously described for library quantification for ATAC-seq^77^ and CUT&Tag^78^. Briefly, 1 μL of purified LagTag amplicon was used as input for a qPCR assay using the corresponding i5 and i7 index primers at 250 nM with iQ SYBR Green Supermix (Bio-Rad) in a total reaction volume of 10 μL. Amplicons were detected on a Bio-Rad CFX96 thermocycler with the following cycling conditions: 95°C for 3 minutes, followed by 39 cycles of 95°C for 15 seconds and 62°C for 30 seconds. Cycle threshold (Cq) values were determined using the auto-threshold function of the CFX96 software. Each condition was assessed with three biological replicates, with no technical replicates. Parental cells lacking the transgene were included as a negative control; a no-template control using primers alone was also included for the baseline.

#### Cell viability assay during differentiation

The parental mESC-Gata4 line and a derivative engineered single clone were used to assess cell viability during differentiation. On Day 0, cells were maintained in 2i medium with or without Shield-1 (16 hours) prior to passaging. On Day 1, cells were seeded at 25,000 cells per well in 96-well plates coated with human laminin and placed under either differentiation medium or 2i medium without Shield-1. Cell viability was assessed at 0, 12, and 24 hours after the Day 1 transfer. Three biological replicates were performed.

At each time point, cells were dissociated into a single cell suspension by aspirating the culture medium, washing once with 1×PBS, and incubating with Accutase at 37°C for 5 minutes. The enzymatic reaction was quenched by adding an equal volume of mESC culture medium, and cells were pelleted by centrifugation at 300×g for 5 minutes. The supernatant was aspirated, and the pellet was resuspended in staining buffer (PBS with 0.1% BSA) at 1 × 10^6^ cells/mL.

To determine cell viability, a working solution of 0.1 μM Calcein AM (Thermo Fisher C3099; 1 mM stock in anhydrous DMSO) was prepared in staining buffer. Cells were incubated for 20 minutes at 37°C in the dark, washed once with 2 mL staining buffer, and centrifuged to remove extracellular dye. The final pellet was resuspended in a 100 μL staining buffer and filtered through a 40 μm filter mesh before acquisition.

Flow cytometry was performed on a Beckman Coulter CytoFLEX S. For data analysis, events were first gated on a forward scatter (FSC) versus side scatter (SSC) plot to exclude debris, followed by an FSC-A versus FSC-H gate to exclude doublets. Viable cells were identified within the singlet gate based on Calcein AM fluorescence (ex/em: 488/525 nm).

#### LagTag and CUT&Tag data processing

To demultiplex the resulting sequencing libraries, we generated fastq files from the Aviti sequencer output using the bases2fastq tool (Element Biosciences, version 1.8.0). For the barcoded nb-Tn5 samples, we then assigned reads to each barcode using custom bash scripts making use of basic shell commands and the ‘seqtk’ package (seqtk-v1.5 https://github.com/lh3/seqtk). Briefly, we generated subsetted R1 and R2 fastq files containing reads for each individual designed barcode sequence. We then identified paired R1/R2 reads for each possible combination of 5’ and 3’ barcode sequences and stored these as paired fastq files representing each barcode combination. These paired files were used for downstream data processing.

LagTag and CUT&Tag sequencing data produced in this study were then processed using the nf-core/cutandrun pipeline^59^ (version 3.2.2) with Nextflow^60^ (version 23.10.1). Briefly, sequencing reads were aligned to the mm10 or hg38 reference genome using bowtie2^61^ (version 2.4.4), keeping only reads with a minimum alignment q score of 20. For the barcoded samples, we also removed 42 bp from the 5’ end with parameters --clip_r1 42 --clip_r2 42. BigWig files were generated with defined bin sizes using the bamCoverage function of deepTools^62^ (version 3.5.1) with Counts Per Million mapped reads (CPM) normalization. Similarly, the BigWig files for log2 ratio of LagTag profiles between Lamin B1 and mCherry were generated with defined bin sizes using the bamCompare function of deepTools. The CUT&RUN suspect list regions^58^ were masked. The processed files were then visualized with Integrative Genomics Viewer (IGV)^63^ (version 2.16.2).

#### Publicly available datasets

For the mESC analyses, we downloaded ChIP-seq datasets from the ENCODE portal^20,79^ for H3K4me3 (ENCSR212KGS) and H3K27me3 (ENCSR059MBO) and from the NCBI GEO (accession GSE195840)^37^ for RNAPIISer2p. We also used previously called peaks from H3K4me3 ChIP-seq (ENCSR212KGS), H3K27me3 ChIP-seq (https://zenodo.org/records/5507375)^23,80^, and RNAPIISer2p CUT&Tag (accession GSE171554)^23^ by merging peaks from replicates using bedtools (version 2.31.1) intersect^64^. For the Lamin B1 analyses, we downloaded pA-DamID datasets (accession GSE181693)^33^ and lamina-associated domains (LADs) from Peric-Hupkes et al.^55^ for mESCs as well as pA-DamID (4DNESWB729QB)^56^, DamID (4DNES24XA7U8), and TSA-seq (4DNESTE8NJOE)^57^ datasets from the 4D Nucleome data portal^81,82^ for HCT116 cells. The mm9 genomic coordinates were converted to mm10 with the UCSC Genome Browser^65^ program LiftOver. The bigwig files for replicates were averaged with deepTools^62^ (version 3.5.1) bigwigAverage.

#### Correlation analysis

Pearson or Spearman correlation coefficients between LagTag (CPM or mCherry log2 ratio normalized), CUT&Tag (CPM), ChIP-seq (input normalized), pA-DamID (Dam-only normalized), DamID (Dam-only normalized), and TSA-seq (input normalized) datasets were calculated with 10-kb binning for chr1-19, X (mESCs) or 20-kb binning (HCT116 cells) using the pearsonr or spearmanr function of scipy.stats^68^ (version 1.11.3). When specified, a subset of 10-kb bins (n = 61,536 out of 251,299 total bins) overlapping the union of peaks previously identified from the ChIP-seq (H3K4me3 and H3K27me3) and CUT&Tag (RNAPIISer2p) datasets in mESCs was used, similar to previous benchmarking^34^. We note that in DamID assays, untethered methyltransferase preferentially labels accessible chromatin^83^, and log2 ratio normalization over a Dam-only control is typically used to correct this bias^14^. We compared CPM and log2 ratio normalized LagTag profiles with orthogonal datasets. Log2 ratio normalization did not improve correlation with orthogonal profiles except for Lamin B1 pA-DamID, suggesting that open chromatin bias does not substantially confound LagTag enrichment signals at the 10-kb resolution used in this study. However, chromatin accessibility bias may pose a greater concern for low-abundance targets such as transcription factors, which were not tested here.

#### Signal enrichment

To generate signal enrichment plots, the functions computeMatrix and plotHeatmap from deepTools^62^ (version 3.5.4) were used with the flag --missingDataAsZero. computeMatrix was used in reference-point mode for plots using peak centers and scale-regions mode for plots over gene bodies or LADs. For plots over peak centers or genes, the relevant bigwigs averaged across replicates with a bin size of 50 bp were used, while for plotting for LADs, a bin size of 10 kb was used.

Protein-coding genes were taken from mm10 vM21 annotation (ENCODE accession number: ENCSR884DHJ; filename: mm10_gencode.vM21.primary_assembly.annotation_UCSC_names.gtf). For the gene body heatmaps, these gene annotations were filtered for those with gene_type “protein_coding”.

#### Differential enrichment analysis

To perform differential enrichment analysis of RNAPIISer2p signals over gene bodies, deepTools multiBamSummary was first used to generate counts per gene using bam files generated by the cutandrun pipeline^59^. Genes were defined by mm10 vM21 annotation as above but not filtered for protein-coding status. Counts per gene were normalized by dividing by the number of mapped and paired reads per sample. This information was extracted from the cutandrun pipeline results.

Then DESeq2^66^ (version 1.34.0) was used to determine differentially enriched genes with “condition” being the only factor in the design formula. Raw (unnormalized) counts per gene were used as input. The shrinkage method apeglm^67^ (version v1.32.0) was used to shrink log2 fold change estimates. After shrinkage, a gene was deemed differentially enriched between two conditions if the absolute value of log2 fold change exceeded 2 and adjusted P-value was below 0.001.

To generate principal component analysis coordinates, raw counts per gene were first standardized using the DESeq2 variance standardized transformation^66^. Then the prcomp function in R (scale. = FALSE) was run on the transformed counts using the top 500 most variable genes.

The genes displayed in the clustermap were determined by generating a consensus list of differentially enriched genes between conditions 5 and 6 in both the engineered and parental mESC-Gata4 cell lines. The normalized count values over these genes were then processed as follows: the counts were averaged across replicates, then normalized by row (gene) so that each row has a mean of 0 and standard deviation of 1 using the zscore function from scipy.stats^68^. The clustermap function of seaborn^69^ (version 0.11.2) was then used with method “average” to determine row linkage.

#### Cell division dilution model

The number of cell divisions after induction of the Shield-1-inducible M.EcoGII fusion protein was estimated using the ratio of sequencing read counts as a linear proxy for variable target abundance (i.e., m6A) relative to the constitutive target (i.e., RNAPIISer2p). Specifically, in LagTag measuring both m6A and RNAPIISer2p targets, the ratio is defined as:

$$r=N_{m6A}/N_{\text{RNAPIISer2p}}$$

where *N*_*m6A*_ represents read counts from the inducible m6A target, assumed to be diluted by cell division, and *N*_*RNAPIISer2p*_ represents those from the constitutive target. Because RNAPIISer2p target abundance is assumed constant, the ratio *r* is proportional to m6A target abundance. Similarly, the read fraction of m6A antibody was calculated from the same read counts as:

$$f=N_{m6A}/\left(N_{m6A}+N_{\text{RNAPIISer}2p}\right)$$

which is related to the ratio by:

f=r/(1+r)

Assuming symmetric partitioning of m6A-marked DNA to daughter cells upon replication^16^, the m6A signal above basal is halved each cell cycle, giving after *n* cell divisions:

$$r_{t1}=r_{0}+\left(r_{t2}-r_{0}\right)/2^{n}$$

Solving for *n*:

$$n=log_{2}\left(\left(r_{t2}-r_{0}\right)/\left(r_{t1}-r_{0}\right)\right)$$

where *r*_*0*_ is the ratio measured in uninduced cells, reflecting background m6A signal in the absence of Shield-1 induction; *r*_*t2*_ is the ratio in cells induced for duration *Δt* ending at timepoint *t2* without no subsequent chase (fully induced reference); and *r*_*t1*_ is the ratio in cells induced for *Δt* before the *t1* timepoint, then chased for (*t*_*2*_ – *t*_*1*_) without Shield-1 induction. Using this model, we estimated the number of cell divisions for experimental samples at the 24-hour chase timepoint, and computed the predicted dilution curve asymptotically approaching the basal level. To quantify the relative m6A signal abundance above background, enrichment, *E*, was expressed as a normalized fold ratio over the basal level, calculated as:

$$E=r/r_{0}$$

where r_*0*_ is the ratio measured in uninduced cells.

#### Statistics and reproducibility

CUT&Tag and LagTag experiments were performed with two or three replicates using 96-well plates in Figures 1, 2, and Figures S1–10. No statistical methods were used to predetermine the sample size. The experiments were not randomized and the investigators were not blinded to allocation during the experiments and outcome assessment.

## Supplementary Material

1

2

3 **Table S1:** A list of primary antibodies used in this study.

4 **Table S2:** A list of oligonucleotides used in this study.

5 **Table S3:** Summary of temporal chromatin profiling technologies.

## Figures and Tables

**Table:** Key resources table

REAGENT or RESOURCE	SOURCE	IDENTIFIER
Antibodies		
Rabbit monoclonal anti-N6-methyladenosine (m6A) [Ab1]	Invitrogen	Cat#MA5–35350; RRID: AB_2849251
Rabbit polyclonal anti-N6-methyladenosine (m6A) [Ab2]	Millipore	Cat#ABE572-I; RRID: AB_2892214
Rabbit monoclonal anti-N6-methyladenosine (m6A) [Ab3]	Cell Signaling Technology	Cat#56593T; RRID: AB_2799515
Rabbit monoclonal anti-N6-methyladenosine (m6A) [Ab4]	Sigma-Aldrich	Cat#SAB5600251; RRID: AB_2892215
Rabbit monoclonal anti-RNA Polymerase II CTD phospho-Ser2 (RNAPII Ser2p)	Cell Signaling Technology	Cat#13499S; RRID: AB_2798238
Rabbit monoclonal anti-H3K4me3	Cell Signaling Technology	Cat#9751T; RRID: AB_2616028
Rabbit monoclonal anti-H3K27me3	Cell Signaling Technology	Cat#9733S; RRID: AB_2616029
Rabbit polyclonal anti-Lamin B1	Abcam	Cat#ab16048; RRID: RRID: AB_443298
Rabbit monoclonal anti-IgG	Cell Signaling Technology	Cat#3900S; RRID: RRID: AB_1550038
Mouse monoclonal IgG1 anti-RNA Polymerase II CTD phospho-Ser2 (RNAPII Ser2p)	Cosmo Bio USA	Cat#MCA-MABI0602–100-EX; RRID: AB_2819246
Mouse monoclonal IgG1 anti-H3K27me3	Active Motif	Cat#61018; RRID: AB_2614987
Bacterial and virus strains		
SHuffle T7 Competent *E. coli*	New England Biolabs	Cat#C3026J
ArcticExpress (DE3) Competent *E. coli*	Agilent Technologies	Cat#230192
Biological samples		
		
Chemicals, peptides, and recombinant proteins		
Gelatin solution	Sigma-Aldrich	Cat#G1393
DMEM	Gibco	Cat#11960044
ES-grade fetal bovine serum (FBS)	Gibco	Cat#16141061
Leukemia inhibitory factor (LIF), ESGRO	Sigma-Aldrich	Cat#ESG1106
MEM Non-essential amino acids	Gibco	Cat#11140050
Sodium pyruvate	Gibco	Cat#11360070
2-Mercaptoethanol	Gibco	Cat#21985023
Penicillin-Streptomycin-Glutamine	Gibco	Cat#10378016
PD0325901	Sigma-Aldrich	Cat#444966
CHIR99021	Sigma-Aldrich	Cat#SML1046
McCoy’s 5A (modified) medium	Gibco	Cat#16600082
Fetal bovine serum (FBS)	Avantor	Cat#97068–085
Penicillin-Streptomycin	Gibco	Cat#15140122
FuGENE HD Transfection Reagent	Promega	Cat#E2311
Shield-1	Takara Bio	Cat#632189
Human laminin (LN511)	BioLamina	Cat#LN511
Bovine Collagen Type I	Sigma-Aldrich	Cat#C8919
DPBS, no calcium, no magnesium	Gibco	Cat#14190144
Formaldehyde, 16% (w/v), methanol-free	Thermo Scientific	Cat#28908
PBS (10×), pH 7.4, RNase-free	Invitrogen	Cat#AM9624
Triton X-100 solution	Sigma-Aldrich	Cat#93443
RNase A/T1 Mix	Thermo Fisher Scientific	Cat#EN0551
NaCl (5 M), RNase-free	Invitrogen	Cat#AM9759
NaOH (1 N)	Teknova	Cat#H0224
Tris-HCl (1 M), pH 7.5, RNase-free	Invitrogen	Cat#15567027
Tris-HCl (1 M), pH 8.0, RNase-free	Invitrogen	Cat#15568025
UltraPure BSA (50 mg/mL)	Invitrogen	Cat#AM2616
HEPES (1 M), pH 7.4	Teknova	Cat#H1030
MgCl_2_ (1 M)	Invitrogen	Cat#AM9530G
EDTA (0.5 M), pH 8.0, RNase-free	Invitrogen	Cat#AM9260G
TAPS buffer (1 M), pH 8.5	Boston BioProducts	Cat#BB-2375
SDS solution, 10%	Invitrogen	Cat#24730020
Thermolabile Proteinase K	New England Biolabs	Cat#P8111S
DNase/RNase-free distilled water	Invitrogen	Cat#10977015
cOmplete, EDTA-free protease inhibitor cocktail	Roche	Cat#04693132001
Poly(ethylenimine) solution	Sigma-Aldrich	Cat#P3143
Chitin Resin	New England Biolabs	Cat#S6651L
Isopropyl-beta-D-thiogalactopyranoside (IPTG)	Biosynth	Cat#EI07011
RNase Inhibitor, Murine	New England Biolabs	Cat#M0314S
Calcein AM	Thermo Fisher Scientific	Cat#C3099
Recombinant nanobody-Tn5 fusion protein (nb-Tn5)	This paper	N/A
Recombinant M.EcoGII homologs (EspM, PvaM, KpnNIH)	This paper	N/A
Critical commercial assays		
HiScribe T7 High Yield RNA Synthesis Kit	New England Biolabs	Cat#E2040S
RNA Clean & Concentrator-5 Kit	Zymo Research	Cat#R1015
MTase-Glo Methyltransferase Assay	Promega	Cat#V7601
NucleoSpin Gel and PCR Clean-up Kit	MACHEREY-NAGEL	Cat#740609
NEBNext High-Fidelity 2× PCR Master Mix	New England Biolabs	Cat#M0541L
iQ SYBR Green Supermix	Bio-Rad Laboratories	Cat#1708880
Qubit dsDNA High Sensitivity Assay Kit	Invitrogen	Cat#Q32854
Agilent High Sensitivity D1000 ScreenTape	Agilent Technologies	Cat#5067–5584
Agilent High Sensitivity D1000 Sample Buffer	Agilent Technologies	Cat#5067–5603
AVITI 2×150 Sequencing Kit Cloudbreak FS Low Output	Element Biosciences	Cat#860–00011
AVITI 2×150 Sequencing Kit Cloudbreak FS High Output	Element Biosciences	Cat#860–00013
Cloudbreak FS PhiX Control, 3rd Party	Element Biosciences	Cat#830–00023
Custom Primer Set Cloudbreak FS	Element Biosciences	Cat#820–00025
E-Gel EX Agarose Gels, 2%	Invitrogen	Cat#G402022
DNA Clean & Concentrator-5	Zymo Research	Cat#D4014
Zymoclean Gel DNA Recovery Kit	Zymo Research	Cat#D4001
Mag-Bind TotalPure NGS	Omega Bio-Tek	Cat#M1378
Deposited data		
Raw and processed LagTag and CUT&Tag data	This paper	GEO: GSE305653
H3K4me3 ChIP-seq, mESCs	ENCODE	ENCSR212KGS
H3K27me3 ChIP-seq, mESCs	ENCODE	ENCSR059MBO
RNAPIISer2p ChIP-seq, mESCs	Abe et al.^37^	GEO: GSE195840
RNAPIISer2p CUT&Tag, mESCs	Gopalan et al.^23^	GEO: GSE171554
H3K27me3 ChIP-seq, mESCs	Gopalan et al.^23^	Zenodo: https://zenodo.org/records/5507375
Lamin B1 pA-DamID, mESCs	van Schaik et al.^33^	GEO: GSE181693
Lamina-associated domains (LADs), mESCs	Peric-Hupkes et al.^55^	N/A
Lamin B1 pA-DamID, HCT116	van Schaik et al.^56^	4DN: 4DNESWB729QB
Lamin B1 DamID, HCT116	4DN Network	4DN: 4DNES24XA7U8
Lamin B1 TSA-seq, HCT116	Kumar et al.^57^	4DN: 4DNESTE8NJOE
CUT&RUN suspect list regions	Nordin et al.^58^	N/A
Experimental models: Cell lines		
Mouse: E14 embryonic stem cells (E14Tg2a.4)	Mutant Mouse Resource & Research Centers	RRID:MMRRC_015890-UCD
Mouse: mESC-Gata4	Amadei et al.^22^	N/A
Mouse: E14 PGK-DD-linker-M.EcoGII-mCherry	This paper	N/A
Mouse: E14 PGK-DD-linker-M.EcoGII-mCherry-DD	This paper	N/A
Mouse: E14 PGK-DD-linker-M.EcoGII-Ser2p mintbody	This paper	N/A
Mouse: E14 PGK-DD-linker-M.EcoGII-Ser2p mintbody-DD	This paper	N/A
Mouse: E14 PGK-DD-linker-M.EcoGII-Lamin B1	This paper	N/A
Mouse: E14 PGK-DD-linker-M.EcoGII-TAF3 (3× chromodomain)-DD	This paper	N/A
Mouse: E14 PGK-DD-linker-M.EcoGII-CBX7 (2× chromodomain)-DD	This paper	N/A
Mouse: E14 PGK-DD-linker-EspM-TAF3 (3× chromodomain)-DD	This paper	N/A
Mouse: mESC-Gata4 PGK-DD-linker-M.EcoGII-Ser2p mintbody-DD	This paper	N/A
Human: HCT116	ATCC	Cat#CCL-247; RRID: CVCL_0291
Human: HCT116 PGK-DD-linker-M.EcoGII-mCherry	This paper	N/A
Human: HCT116 PGK-DD-linker-M.EcoGII-Ser2p mintbody	This paper	N/A
Human: HCT116 PGK-DD-linker-M.EcoGII-LaminB1	This paper	N/A
Experimental models: Organisms/strains		
		
Oligonucleotides		
Oligonucleotides used in this study	Integrated DNA Technologies	See Table S2
Recombinant DNA		
pRetroX-PTuner DD-linker-M.EcoGII	Sobecki et al.^15^	Addgene Plasmid #122082
pRetroX-PTuner DD-linker-M.EcoGII-v5-Lamin B1	Sobecki et al.^15^	Addgene Plasmid #122083
pTXB1-nbOcIgG-Tn5	Stuart et al.^25^	Addgene Plasmid #184285
pTXB1-nbMmIgG1-Tn5	Stuart et al.^25^	Addgene Plasmid #184287
PB-PGK-DD-linker-M.EcoGII-mCherry	This paper	N/A
PB-PGK-DD-linker-M.EcoGII-mCherry-DD	This paper	N/A
PB-PGK-DD-linker-M.EcoGII-Ser2p mintbody	This paper	N/A
PB-PGK-DD-linker-M.EcoGII-Ser2p mintbody-DD	This paper	N/A
PB-PGK-DD-linker-M.EcoGII-Lamin B1	This paper	N/A
PB-PGK-DD-linker-M.EcoGII-M.EcoGII-TAF3 (3× chromodomain)-DD	This paper	N/A
PB-PGK-DD-linker-M.EcoGII-M.EcoGII-CBX7 (2× chromodomain)-DD	This paper	N/A
PB-PGK-DD-linker-EspM-TAF3 (3× chromodomain)-DD	This paper	N/A
Software and algorithms		
Custom written scripts	This paper	CaltechDATA: https://doi.org/10.22002/abcrz-g3683
Bases2Fastq, v1.8.0	Element Biosciences	https://docs.elembio.io/docs/bases2fastq/setup/
seqtk, v1.5	GitHub	https://github.com/lh3/seqtk
nf-core/cutandrun, v3.2.2	Ewels et al.^59^	https://nf-co.re/cutandrun
Nextflow, v23.10.1	Di Tommaso et al.^60^	https://www.nextflow.io
Bowtie2, v2.4.4	Langmead and Salzberg^61^	http://bowtie-bio.sourceforge.net/bowtie2/
deepTools, v3.5.1 / v3.5.4	Ramírez et al.^62^	https://deeptools.readthedocs.io
Integrative Genomics Viewer (IGV), v2.16.2	Robinson et al.^63^	https://igv.org
BEDTools, v2.31.1	Quinlan and Hall^64^	https://bedtools.readthedocs.io
UCSC Genome Browser LiftOver tool	Kent et al.^65^	https://genome.ucsc.edu/cgi-bin/hgLiftOver
DESeq2, v1.34.0	Love et al.^66^	https://bioconductor.org/packages/DESeq2/
apeglm, v1.32.0	Zhu et al.^67^	https://bioconductor.org/packages/apeglm/
SciPy, v1.11.3	Virtanen et al.^68^	https://scipy.org
seaborn, v0.11.2	Waskom^69^	https://seaborn.pydata.org
Ident and Sim	Stothard^70^	https://www.bioinformatics.org/sms2/ident_sim.html
Bio-Rad CFX Maestro Software, v4.0.2325.0418	Bio-Rad Laboratories	Cat#12013758
Other		
μ-Plate 96 Well Round, ibiTreat	ibidi	Cat#89606
Amicon Ultra Centrifugal Filter Unit, 10 kDa MWCO	Millipore	Cat#UFC9010
Amicon Ultra Centrifugal Filter Unit, 30 kDa MWCO	Millipore	Cat#UFC9030
PCR Plate Sealing Film, adhesive	Bio-Rad Laboratories	Cat#MSB1001B
Econo-Column chromatography column	Bio-Rad Laboratories	Cat#7371022
CFX96 Real-Time PCR Detection System	Bio-Rad Laboratories	Cat#185–5095
AVITI Sequencing System	Element Biosciences	Cat#880–00001
CytoFLEX S flow cytometer	Beckman Coulter	Cat#C09766

## Highlights

- LagTag links past and present chromatin states from the same population
- Time-gated m6A writing records DNA locations near chromatin-associated proteins
- Antibody-guided tagmentation jointly recovers recorded and endpoint marks
- LagTag resolves pre- and post-differentiation chromatin signatures
