# Supplementary material for: Genome-wide chromatin recording resolves dynamic cell state changes
Source: Cell Syst. Author manuscript; Available in PMC 2026 Sep 3. (PMC13540299; doi:10.1016/j.cels.2026.101717)
Supplement: Supplementary file 1 [file NIHMS2204262-supplement-1.pdf]

Cell Systems

Transparent Peer Review Record

Summary

Received December 22, 2025
Preprint: doi.org/10.1101/2025.08.05.668773
Scientific editor: Bernadett Gaal, DPhil

Revision invited March 24, 2026

Revision received April 30, 2026

Accepted August 4, 2026

This Transparent Peer Review Record is not systematically proofread, type-set, or edited. Special characters, formatting, and equations may fail to render properly. Standard procedural text within the editor's letters has been deleted for the sake of brevity, but all official correspondence specific to the manuscript has been preserved.

Editorial decision letter with reviewers' comments, first round of review

Dear Michael,

I'm enclosing the comments that reviewers made on your paper, which I hope you will find useful and constructive. As you'll see, they express interest in the study, but they also have a number of criticisms and suggestions. Based on these comments, it seems premature to proceed with the paper in its current form; however, if it's possible to address the concerns raised with additional experiments and analysis, we'd be interested in considering a revised version of the manuscript.

As a matter of principle, I usually only invite a revision when I'm reasonably certain that the authors' work will align with the reviewers' concerns and produce a publishable manuscript. In the case of this manuscript, while we would not expect inclusion of major biological insights, it would be important to demonstrate with data the key advantages and enabling potential of LagTag.

I'm always happy to talk, either over email or video call, if you'd like feedback about whether your efforts are moving the manuscript in a productive direction. Do note that we generally consider papers through only one major round of revision, so the revised manuscript would be either accepted or rejected based on the next round of comments we receive from the reviewers. If you have any questions or concerns, please let me know. More technical information and advice about resubmission can be found below my signature. Please read it carefully, as it can save substantial time and effort later.

If any changes to the authorship are needed (addition, removal, or a change in order of authors), we require that an authorship change form be provided with the revised manuscript that indicates the reason for the change and provides written consent for the change from all authors, including any that were removed.

I look forward to seeing your revised manuscript.

All the best,

Bernadett

Bernadett Gaál, DPhil
Editor-in-Chief, Cell Systems

Reviewers' comments:

Reviewer #1: Chromatin landscapes influence transcriptional competence, signal responsiveness, and lineage commitment; however, most genome-wide profiling technologies (ChIP-seq, ATAC-seq, CUT&Tag, DamID) provide only static endpoint measurements, while the ability to record chromatin states is essential for establishing causal links between prior epigenetic configurations and subsequent cellular outcomes. At present, temporal relationships are typically inferred indirectly by sampling parallel populations at successive timepoints, reconstructing pseudotemporal trajectories, or comparing lineage-related cells. These approaches cannot recover the actual chromatin configuration that existed in a cell before a state transition. This shortcoming limits mechanistic interpretation. Previous chromatin-state embedding strategies partially addressed this gap by exploiting bacterial DNA methyltransferases to record protein-DNA interactions as genomic methylation marks in living cells. More recent methods moved toward temporal resolution of chromatin states. Nevertheless, these approaches are constrained by short effective recording windows due to DNA replication dynamics, technical complexity, or limited scalability.

In this work, the authors present LagTag, a new method that couples transient adenine methylation recording with CUT&Tag to retrieve prior and endpoint chromatin states from the same mammalian cells, enabling within-cell temporal comparisons. This has not been possible with standard assays. Using an anti-m6A antibody rather than restriction digestion, the method permits detection of hemi-methylated DNA and thereby extends the functional recording window across cell cycles.

This manuscript presents a valuable assay with potential for broader use by the community. That said, several points should be clarified or strengthened.

The authors should provide a quantitative characterization of the temporal resolution of LagTag. How does M.EcoGII expression duration affect the temporal window captured, and how LagTag compare to other temporal methods? Indeed, LagTag appears to function to reflect chromatin occupancy over the pulse window. It would be helpful if the authors stated/discussed whether LagTag captures instantaneous chromatin states at pulse onset or cumulative occupancy during the pulse, or an average shaped by protein residence time and methyltransferase kinetics. (A conceptual model of how binding dynamics translate into m6A signal would be very helpful for the readership.)

Furthermore, experimental comparison to related temporal methods (Dam&ChIC, CUT&TIME) would help the authors clearly state the relative advantage of their protocol vis-a-vis the published assays. At a minimum, provide a quantitative comparison of the effective recording window, sensitivity, and scalability relative to other methods.

Likewise, while the assay was nicely shown to work in the (somewhat artificial) forced endoderm differentiation system, it would be essential to control whether Dam expression might perturb chromatin state, cell viability, or differentiation kinetics of the assayed system and in a regular differentiation scheme. The authors should compare differentiation of the lines with their constructs to the parental ES cell line directly (PCA, DESeq2 on RNA-seq with distinct constructs).

The mCherry-M.EcoGII control shows enrichment in accessible chromatin. The authors should clarify how this baseline accessibility bias affects the interpretation of signal-to-background ratios for low-occupancy targets. How should the normalization be performed to control for this bias?

While LagTag enables recovery of earlier and endpoint chromatin states from the same cells, it remains important to clarify what types of mechanistic conclusions can and cannot be drawn from these data. Specifically, the method records occupancy history but does not directly resolve whether earlier chromatin states causally instruct subsequent states or merely correlate with them. A more explicit discussion of how LagTag distinguishes instructive epigenetic memory from passive persistence would strengthen the conceptual positioning of the approach. Demonstrating an example where LagTag reveals a previously unobservable chromatin-fate relationship would strengthen the manuscript beyond proof-of-concept validation.

Reviewer #2: This manuscript reports LagTag, a method to simultaneously map two chromatin features: one as it is at the time of cell harvest, and one that was recorded earlier. It consists of a combination of MadID (to record and map the earlier feature) and CUT&Tag (to map the present feature). Proof-of-concept is demonstrated in a simple cell culture differentiation model.

The experiments are carefully designed and performed, and the figures are prepared with care. Clearly, a lot of work and dedication went into this. The text is also well-written and balanced.

Nevertheless, this study pales in comparison to the previous Dam&ChIC paper (Kefalopoulou, Nat Cell Biol 2025), which had a similar scope and m6A-based approach, but was much more exciting because Dam&ChIC yields recorded and present data from single cells. Furthermore, the current manuscript does not provide any new biological insights, unlike the Kefalopoulou study. Compared to Dam&ChIC, LagTag is probably less challenging to perform (as LagTag is not single-cell).

The authors argue that the MadID component of LagTag has an advantage over the scDamID component of Dam&ChIC, because scDamID in theory cannot record/detect a chromatin state that occurs prior to S-phase due to the inability of DpnI to cut hemi-m6A DNA. This is probably true. However, the current manuscript does not demonstrate conclusively that LagTag indeed has this longer "memory" (see below). It also does not explore the limits of this potential advantage of LagTag: over how many cell cycles would the "memory" be retained and detectable? This is relevant, because the DCM-TimeMachine method (Boers, NatBiotech 2023; based on m5C) was reported to memorize earlier chromatin states over several cell cycles.

The authors mention that two other LagTag-like studies have been submitted by other labs, and it seems that they rushed out this manuscript because of this situation. I understand this pressure, but at present the manuscript - while technically sound - seems incomplete: (1) it lacks demonstration that interesting new biology can be discovered; (2) it does not compellingly demonstrate the extended memory ability compared to Dam&ChIC.

Major points:

1. The results in Figure 2 are encouraging, but are strictly a proof-of-concept. The same maps could have been obtained by straightforward CUT&Tag on undifferentiated and differentiated cells (and probably more accurately), so there is no added value here. For cells in a dish, I could not think of an application where LagTag would provide data that cannot be obtained by simply harvesting cells before and after the stimulus that triggers their transition. It seems that LagTag may only have added value in more complex developmental biology settings? I realize it would be a lot of work, but demonstration of true added biological value - perhaps in gastruloids - would substantially strengthen this manuscript.

2. Figure 2d+e are encouraging, but are anecdotal. I found Figure 2f very difficult to interpret, largely due to the z-score transformation that completely obscures fold-change effect sizes. I was also confused why the clustering of the genes in the left and right panels was not kept the same. Importantly, I am missing more rigorous benchmarking of the recorded signal. I suggest a simple scatterplot that shows, for all genes, the fold-change between present and recorded according to LagTag versus the fold-change between differentiated and undifferentiated cells according to conventional CUT&Tag. Ideally, this would yield a plot with most genes close to the diagonal. But I am open to other systematic quantitative benchmarking, for example estimates of false-positives and false-negatives in a binarised setting.

3. The data from the ESC->endoderm differentiation model are interpreted as demonstration that m6A-memory is retained over mitosis/S-phase. However, I could not find evidence that the cells continue to cycle when differentiation is trigged by GATA4 induction. Did the harvested cells really go through S-phase during the "chase" period?

4. Over how many cell cycles can a pattern be memorized and recovered? It should be fairly straightforward to test this in the cell differentiation model.

=============== Reviewed by Bas van Steensel. Review task accepted: 2 March 2026; completed: 12 March 2026. It is my standard policy to disclose my identity on *all* my review reports, regardless of my comments and recommendations. All correspondence should go via the editor. PLEASE DO NOT REMOVE THIS NOTE. =========

Reviewer #3: In this study, Takei et al. introduce a very elegant approach to obtain snapshots of molecular events that occur in cells as a function of time jointly with their associated chromatin state. By using an adenine DNA methyltransferase that is found in bacteria, the authors could achieve high efficiency labeling of DNA and at the same time perform CUT and Tag for any epitope of interest. This is a method that is very likely to have broad interest for the chromatin community and those interested in obtaining temporal information about cellular states. The optimizations of particularly challenging experimental steps to ensure that the authors obtain high quality data is especially commendable. The authors also pointed out parallel publications using a similar approach and it clearly is a testament to the strength of such recording approaches and highlights an important direction for the field moving forward.

Major Comments:

1) The main issue I have with the paper is how dense the structure and text is. Clearly this is a paper written for a journal format that doesn't offer flexibility for page length which makes following the logic, the figures and the outcomes of the experiment very challenging. Personally, I felt that it does significantly undersells the very elegant and truly exceptional data that this manuscript presents. Splitting figures and using the text to lay out the arguments more clearly would substantially improve readability.
2) There is one major conceptual point I do not fully understand: what is the value of marking the initial chromatin state with DNA methylation, given that this state can be fully characterized simply as whatever exists at t = 0? Since the methylation pattern does not evolve spatially over time, beyond a slow decay it tells us only how things began, while the chromatin state itself must still convey how things are changing. It is true that CUT&Tag does not record prior events, but in this implementation, methylation appears functionally equivalent to CUT&Tag performed at t = 0.
To be more specific: in Figure 1h, a plot is shown with "recorded" and "endpoint" measurements. Is the primary purpose of this figure to demonstrate joint profiling of DNA methylation and histone modifications? If so, it is not clear to me where the temporal component enters the scheme. I can only assume the main payoff will come when this method is adapted to a single-cell format, but I would appreciate clarification on this point.
3) Have the authors generated data in which methylation exhibits a decay over time? If so, this would be valuable to highlight in the main figures, along with a characterization of the dilution rate, to provide an estimate of how long the signal lasts. Sorry if the data is in the manuscript and I missed it.
4) Relatedly, how short can the labeling pulse be? The labeling times reported here are quite long, likely reflecting limitations in enzyme activity or abundance, both of which presumably require careful

titration to prevent non-specific labeling.
5) Since these experiments are performed on cell populations rather than individual cells, the claim that LagTag enables observation of the same cell at different time points appears to be incorrect.
Terminology
I found the terminology in the manuscript very hard to follow. Is recorded the same as pulse labeling with methylation? Given its tense, is recording not a continuous process? how is it different from using the well-established and widely used terminology of pulse and chase? It seems to me that pulse and chase are a lot more straightforward unless the recording has some special implication, but this was also not clear. Also, LagTag is interchangeably used with many of these words in the manuscript and I couldn't figure out which part of the protocol is recording, end point and LagTag. Then to make matters worse, there are two other terms past and present?

Authors' response to the reviewers' first round comments

Attached.

Editorial decision letter with reviewers' comments, second round of review

Dear Michael,

I'm very pleased to let you know that the reviews of your revised manuscript are back, the peer-review process is complete, and only a few minor, editorially-guided changes are needed to move forward towards publication. As you will note, Reviewer 1 has requested direct experimental comparisons with other methods; however, in keeping with our earlier discussions, I am happy to proceed without these. That said, please do ensure the manuscript explicitly addresses the limitations of the current approach, in line with Reviewer 3's recommendation.

In addition to the final comments from the reviewers, I've made some suggestions about your manuscript within the "Editorial Notes" section below. Please review these notes along with the detailed formatting requirements listed in the Final Files Checklist. We've also put together this FAQ (click the Final Formatting Checks tab) for your convenience. Please ask any questions you may have, make any necessary changes to your manuscript files, and then upload your final files into Editorial Manager. Once we receive your formatted files, we will go through our formatting checks and let you know if further changes are needed.

We hope to receive your formatted files within 5 business days. Please email me directly if this timing is a problem or you're facing extenuating circumstances. Alternatively, if this manuscript needs to be officially accepted by a particular date because of grant deadlines, applications, or because it will help your trainees, please let me know.

To standardize the referencing style across Cell Press journals, we ask that all in-text citations be formatted as superscripted numbers (e.g. "Multiple reports support this observation.[1,2]"). Moving away

from the Harvard referencing style (e.g. Smith *et al*., 2020) will improve author and reader experiences. All manuscripts accepted from now on must use **the superscript numbered Cell Press referencing style**. Make sure to use this numbered referencing style for all new and revised submissions as well. Switching is easy. Just use the updated CSL and EndNote referencing styles for Cell Press articles.

If any changes to the authorship are needed (addition, removal, or a change in order of authors), we require that an authorship change form be provided with the revised manuscript that indicates the reason for the change and provides written consent for the change from all authors, including any that were removed.

Below my signature, you'll find specific information about what to expect next regarding formatting checks and working with our Production Department after acceptance. It's been a pleasure working with you, please feel free to contact our journal team with questions.

All the best,

Bernadett

Bernadett Gaál, DPhil
Editor-in-Chief, Cell Systems

Editorial Notes

Transparent Peer Review: Thank you for electing to make your manuscript's peer review process transparent. As part of our approach to Transparent Peer Review, we ask that you add the following sentence to the end of your abstract: "A record of this paper's Transparent Peer Review process is included in the Supplemental Information." Note that this ***doesn't*** count towards your 150 word total!

Manuscript Text:

- House style disallows editorializing within the text (e.g. strikingly, surprisingly, importantly, etc.), especially the Results section. These terms are a distraction and they aren't needed—your excellent observations are certainly impactful enough to stand on their own. Please remove these words and others like them. "Notably" is suitably neutral to use once or twice if absolutely necessary.

Figures and Legends:

Please look over your figures keeping the following in mind:

- When data visualization tools are used (e.g. UMAP, tSNE), please ensure that the dataset being visualized is named in the figure legend and, when applicable, its accession number is included.

- When color scales are used, please define them, noting units or indicating "arbitrary units," and specify whether the scale is linear or log.
- Please ensure that all figures included in your point-by-point response to the reviewers' comments are present within the final version of the paper, either within the main text or within the Supplemental Information.

Resource Availability: Please note that Cell Press has recently changed the way it approaches "availability" statements for the sake of ease and clarity. Please revise your resource availability section as follows, noting that the examples used might not pertain to your study. Please note that the Resource Availability section should immediately follow the Discussion section in the main manuscript.

RESOURCE AVAILABILITY

Lead Contact: Further information and requests for resources and reagents should be directed to and will be fulfilled by the Lead Contact, Jane Doe (janedoe@qwerty.com).

Materials Availability: This study did not generate new materials. -*OR*- Plasmids generated in this study have been deposited at [Addgene, name and catalog number]. -*OR*- etc.

Data and Code Availability:

- **Source data statement** (described below)
- **Code statement** (described below)
- Any additional information required to reanalyze the data reported in this paper is available from the lead contact upon request.

Data and Code Availability statements **have three parts and each part must be present. Each part should be listed as a bullet point, as indicated above.**

Instructions for section 1: Data. The statements below may be used in any number or combination, but at least one must be present. They can be edited to suit your circumstance. ***Please ensure that all datatypes reported in your paper are represented in section 1.*** For more information, please consult this list of standardized datatypes and repositories recommended by Cell Press.

• [Standardized datatype] data have been deposited at [datatype-specific repository] and are publicly available as of the date of publication. Accession numbers are listed in the key resources table.

• [Adjective] data have been deposited at [general-purpose repository] and are publicly available as of the date of publication. DOIs are listed in the key resources table.

• This paper analyzes existing, publicly available data. These accession numbers for the datasets are listed in the key resources table.

• [Adjective or all] data reported in this paper will be shared by the lead contact upon request.

Instructions for section 2: Code. The statements below may be used in any number or combination, but at least one must be present. They can be edited to suit your circumstance. ***If you are using GitHub, please follow the instructions here to archive a "version of record" of your GitHub repo at Zenodo, then report the resulting DOI. Additionally, please note that the Cell Systems strongly recommends that you also include an explicit reference to any scripts you may have used throughout your analysis or to generate your figures within section 2.***

• All original code has been deposited at [repository] and is publicly available as of the date of publication. DOIs are listed in the key resources table.

Instructions for section 3. Section 3 consists of the following statement: Any additional information required to reanalyze the data reported in this paper is available from the lead contact upon request.

STAR Methods:

STAR Methods follows a standardized structure. Please reorganize your experimental procedures to include these specific headings in the following order: EXPERIMENTAL MODEL AND SUBJECT DETAILS (when appropriate); METHOD DETAILS (required); QUANTIFICATION AND STATISTICAL ANALYSIS (when appropriate); ADDITIONAL RESOURCES (when appropriate). We're happy to be flexible about how each section is organized and encourage useful subheadings, but the required sections need to be there, with their headings. They should also be in the order listed. Please see the STAR Methods guide for more information or contact me for help.

Thank you!

Reviewer comments:

Reviewer #1: Thank you for the revised manuscript. The additional controls are appreciated and address most of my concerns. However, one issue remains unresolved and I cannot recommend acceptance without it being addressed.

This is a methods paper. The core claim is that LagTag offers meaningful advantages over existing approaches. Table S3 lists parameters from published papers across different labs and systems: that is not benchmarking, that is literature summary. To support the central claim, the authors need to actually run LagTag and Dam&ChIC on the same biological system and show the comparison. I am not asking for an exhaustive analysis - one focused experiment would suffice.

The argument that the Methods in Brief format justifies skipping this is not convincing. The format may excuse the absence of new biological discovery, but it does not excuse the absence of evidence that the method offers advantages over what already exists.

I am recommending major revision. The work is technically solid and I want to see it published, but not before this gap is filled.

Reviewer #3: I appreciate the authors of the manuscript taking note of my comments and addressing them. The language in the paper is much improved, and I feel the ambiguities around how the

experiment is conducted are better resolved. The dats is much clearer and easier to process (understand)

There are two key experimental pitfalls that the authors highlight in their response that I want to call attention to. I am not saying these are reasons to make decisions about the manuscript but it would be very valuable to be more explicit about them as limitations. The first concerns applying the method to single cells which is really the payoff for a method such as LagTag. This is a limitation of the current study and remains to be seen in future work as to whether this method will be applicable in the contexts they propose. The second concerns labeling time which is 16 hours. I appreciate the point that it is comparable to other methods but it is still long especially given that that some embryonic cell types divide faster than the labeling time which would dilute signal and potentially increase the labeling time window. My recommendation is still to include a section in the discussion that highlights these pitfalls more expcitly so that users of the approach know what is and isn't working.

Authors' response to the reviewers' second round comments

Attached.

Responses to reviewers' comments:

We thank the reviewers for their constructive comments and suggestions to improve our manuscript. Based on the reviewers' comments, we have revised the manuscript considerably by adding new experiments and analyses.

Specifically, we addressed the following major concerns by the reviewers.

1) Temporal resolution of LagTag: Based on all reviewers' comments, we added new experiments and analyses providing quantitative assessments of the temporal resolution of LagTag, including both the recording time and signal decay time resolution. In particular, we added multiple lines of evidence showing that LagTag tracked back chromatin states after two cell divisions in actively dividing cells in the revised manuscript (**Figure 2G, H, Figure S10B**). To our knowledge, no other adenine methyltransferase-based temporal chromatin method has demonstrated retrospective recording over as many cell divisions. This is also clarified in a new Supplementary Table (**Table S3**).

2) Effects of LagTag on cell physiology: Based on comments by Reviewers 1 and 2, we performed additional control experiments and analyses to evaluate the effects of LagTag on cellular differentiation state and cell cycle progression (**Figure S10**), confirming the minimal effects of the recording on cell physiology in our demonstration.

We have also addressed all other minor and technical comments in the revised manuscript as detailed below. Further, we have updated the repositories to include raw and processed data as well as scripts used for the revision.

We believe these changes substantially improve the clarity and robustness of our manuscript, while keeping the focus on demonstrating the broad enabling potential of LagTag. We highlight the major changes with yellow color in the revised manuscript and provide point-by-point responses with blue texts in this document.

Reviewers' comments:

Reviewer #1: Chromatin landscapes influence transcriptional competence, signal responsiveness, and lineage commitment; however, most genome-wide profiling technologies (ChIP-seq, ATAC-seq, CUT&Tag, DamID) provide only static endpoint measurements, while the ability to record chromatin states is essential for establishing causal links between prior epigenetic configurations and subsequent cellular outcomes. At present, temporal relationships are typically inferred indirectly by sampling parallel populations at successive timepoints, reconstructing pseudotemporal trajectories, or comparing lineage-related cells. These approaches cannot recover the actual chromatin configuration that existed in a cell before a state transition. This shortcoming limits mechanistic interpretation.

Previous chromatin-state embedding strategies partially addressed this gap by exploiting bacterial DNA methyltransferases to record protein-DNA interactions as genomic methylation marks in living cells. More

recent methods moved toward temporal resolution of chromatin states. Nevertheless, these approaches are constrained by short effective recording windows due to DNA replication dynamics, technical complexity, or limited scalability.

In this work, the authors present LagTag, a new method that couples transient adenine methylation recording with CUT&Tag to retrieve prior and endpoint chromatin states from the same mammalian cells, enabling within-cell temporal comparisons. This has not been possible with standard assays. Using an anti-m6A antibody rather than restriction digestion, the method permits detection of hemi-methylated DNA and thereby extends the functional recording window across cell cycles.
This manuscript presents a valuable assay with potential for broader use by the community. That said, several points should be clarified or strengthened.

We thank the reviewer for recognizing the importance of our work and for providing constructive feedback. In response, we have performed additional experiments, analyses, and comparisons that strengthen the robustness and clarity of the manuscript. We provide point-by-point responses below.

The authors should provide a quantitative characterization of the temporal resolution of LagTag. How does M.EcoGII expression duration affect the temporal window captured, and how LagTag compare to other temporal methods? Indeed, LagTag appears to function to reflect chromatin occupancy over the pulse window. It would be helpful if the authors stated/discussed whether LagTag captures instantaneous chromatin states at pulse onset or cumulative occupancy during the pulse, or an average shaped by protein residence time and methyltransferase kinetics. (A conceptual model of how binding dynamics translate into m6A signal would be very helpful for the readership.)

We have added new results comparing the effects of different Shield-1 pulse durations (i.e., expressing M.EcoGII for different time windows) on the genome-wide enrichment of m6A signal (new **Figure S3C**). We observed a drastic increase of m6A enrichment at 16 hours compared to 8 hours of pulse, clarifying that LagTag requires a temporal window (or pulse duration) exceeding 8 hours, consistent with other temporal methods.

C

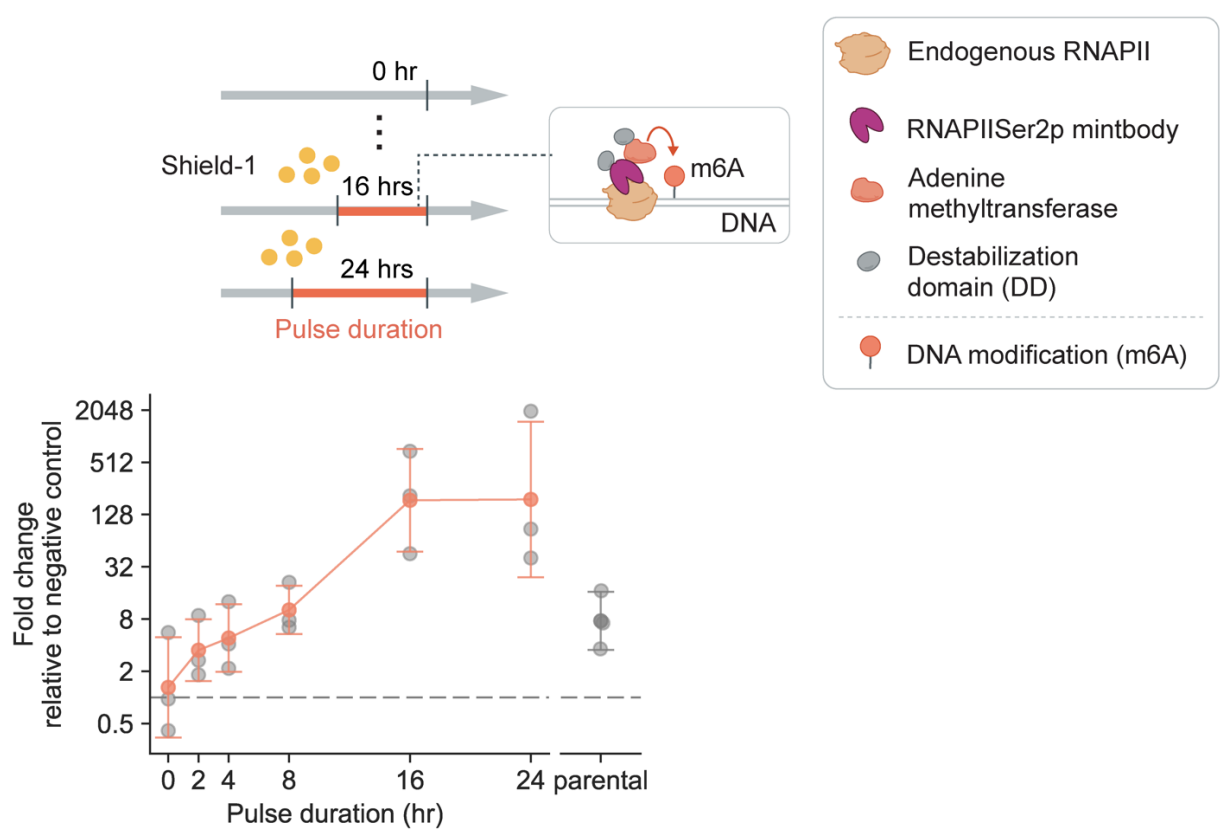

Figure S3C, Comparison of LagTag yield as a function of Shield-1 pulse duration. *Fold change was calculated relative to primer-only controls by qPCR (see STAR Methods). The DD-M.EcoGII-Ser2p-mintbody-DD-expressing mESCs and parental mESCs (control) were used for this assay. Error bars represent mean ± standard deviation with individual replicate data points overlaid (n = 3).*

Additionally, to provide a more comprehensive view across different temporal methods, we added a new **Table S3** that compares pulse durations, showing that LagTag utilizes a similar or shorter pulse duration compared to other methods.

**Method**	**Pulse duration**
DCM-TM	1–8 days
Dam&ChIC	15 hours
HisTrac-seq	1-2 days
CUT&TIME	2 days
LagTag	16 hours

Table S3: Summary of temporal chromatin profiling technologies (selected columns displayed)

We also extended the discussion regarding this temporal resolution of LagTag and its labeling nature in live cells with a reference using biophysical modeling (Redolfi et al. 2019, PMID: 31133702) in the revised manuscript as follows.

*"We note that the m6A signal deposited by LagTag represents a temporal integral of chromatin states during the pulse duration, consistent with biophysical modeling of adenine methyltransferase kinetics (Redolfi et al. 2019). The LagTag pulse duration is similar to or shorter than the other temporal chromatin methods (**Table S3**)."*

Furthermore, experimental comparison to related temporal methods (Dam&ChIC, CUT&TIME) would help the authors clearly state the relative advantage of their protocol vis-a-vis the published assays. At a minimum, provide a quantitative comparison of the effective recording window, sensitivity, and scalability relative to other methods.

Performing side-by-side experimental comparisons with other recently developed state-of-art methods (e.g., Dam&ChIC, CUT&TIME) represents a substantial effort that we would like to consider in the future, but is not feasible in the current work. In lieu of these experiments, we have added a supplementary table

(**Table S3**) providing side-by-side comparisons of related temporal technologies to clarify the differences as follows.

"We provide a summary comparison of recent temporal chromatin profiling methods including these studies (Table S3)."

Likewise, while the assay was nicely shown to work in the (somewhat artificial) forced endoderm differentiation system, it would be essential to control whether Dam expression might perturb chromatin state, cell viability, or differentiation kinetics of the assayed system and in a regular differentiation scheme. The authors should compare differentiation of the lines with their constructs to the parental ES cell line directly (PCA, DESeq2 on RNA-seq with distinct constructs).

To address this point, we performed the viability and CUT&Tag assays during differentiation in the presence or absence of the methyltransferase expression induced by Shield-1.

First, we showed that while prior exposure to Shield-1 modestly reduced the viability of undifferentiated and differentiated mESCs (e.g. 82.6 ± 1.8% with prior Shield-1 addition vs. 87.7 ± 0.5% without Shield-1 for differentiated cells) (new **Figure S10A**), the cycling of cells was minimally perturbed, with both parental and engineered cells dividing approximately twice within 24 hours during differentiation (e.g. 2.1 ± 0.3 and 1.9 ± 0.2 divisions for parental and engineered cells with prior Shield-1 addition, respectively) (new **Figure S10B**).

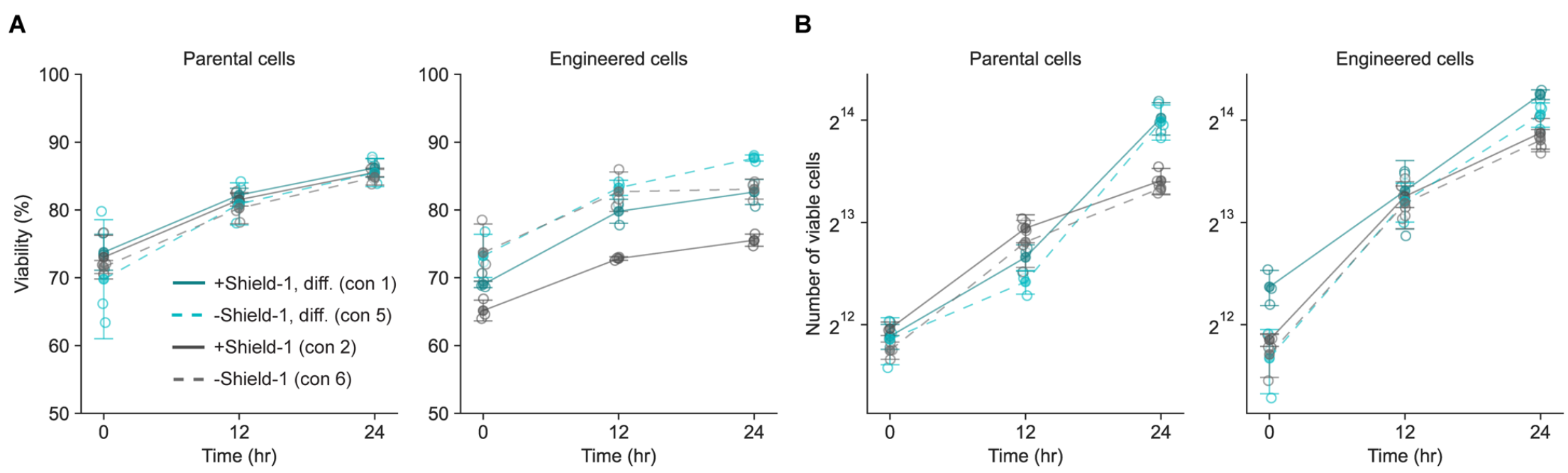

Figure S10A, B, Comparison of viability (A) and number of viable cells (B) with or without prior Shield-1 induction for 16 hours in undifferentiated and differentiated cells (n = 3).

In addition, we performed RNAPIISer2p CUT&Tag experiments to compare the transcriptionally active chromatin states across conditions. The PCA and DESeq2 analyses confirmed that the addition of Shield-1 minimally perturbed the differentiation of mESCs (new **Figure S10C, D**). Specifically, we showed that the major source of RNAPIISer2p enrichment variation along PC1 (90.9%) represents undifferentiated versus differentiated cell states in the PCA (**Figure S10C**). Furthermore, we did not observe a large number of differentially enriched genes in differentiated cells when comparing parental and engineered cells, or when comparing engineered cells with or without Shield-1 exposure (**Figure S10D**). We note that RNAPIISer2p CUT&Tag capturing instantaneous transcriptional states should be a more stringent assay

than the traditional RNA-seq capturing mature transcriptional states accumulated over the past several hours or longer due to the half-life of mRNAs (Sharova et al. 2009, PMID: 19001483).

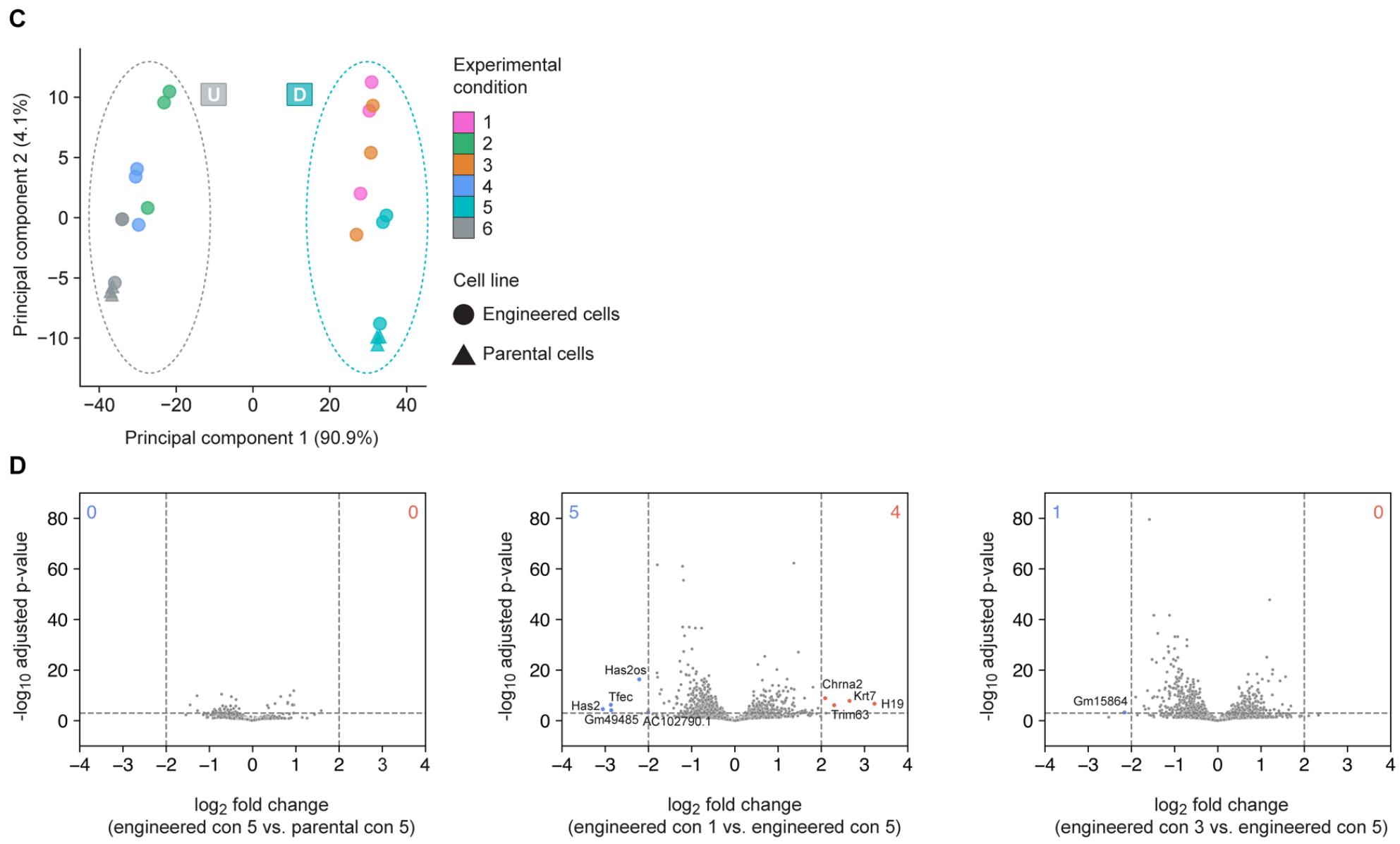

Figure S10C, Principal component analysis of RNAPIISer2p CUT&Tag profiles across different experimental conditions in engineered and parental mESC-Gata4 cells using the 500 most variable genes. D, RNAPIISer2p CUT&Tag analysis comparing signal enrichment over gene bodies between conditions in parental or engineered mESC-Gata4 cells. n =3 for CUT&Tag experiments.

Taken together, these results confirmed the minimal effects of LagTag on cellular differentiation of mESCs.

We have incorporated these new results and discussion in the revised manuscript as described below.

"Furthermore, we confirmed that LagTag recording minimally perturbs cellular physiology during differentiation, as measured by cell viability, cell cycling, and instantaneous transcriptional profiles mapped by RNAPIISer2p enrichment in comparison with parental cells (Figure S10)."

The mCherry-M.EcoGII control shows enrichment in accessible chromatin. The authors should clarify how this baseline accessibility bias affects the interpretation of signal-to-background ratios for low-occupancy targets. How should the normalization be performed to control for this bias?

We agree that untethered methyltransferase may introduce a measurement bias toward accessible chromatin. It is known that untethered methyltransferase (or fluorescently tagged methyltransferase) preferentially labels accessible chromatin in DamID assays (Rooijers et al. 2019, PMID: 31209373). To correct for this open chromatin bias, DamID assays typically calculate the log2 ratio of the target signal over a Dam-only control (Vogel et al. 2007, PMID: 17545983).

To evaluate the effect of open chromatin bias and its normalization in our LagTag data, we performed log2 ratio normalization on RNAPIISer2p and TAF3 over mCherry. In addition, we performed the normalization of Lamin B1 over mCherry in some figures of the original manuscript to directly compare with pA-DamID datasets processed with this normalization. Comparing these normalized profiles against orthogonal chromatin profiling data, we found that log2 ratio normalization did not improve the correlation with the corresponding orthogonal profiles relative to the original profiles except for the comparison with Lamin B1 pA-DamID (**Figure S2G**), suggesting that open chromatin bias does not substantially confound the LagTag-derived enrichment signals and that the profiles reflect genuine target-associated chromatin states rather than an artifact of chromatin accessibility at the 10-kb genomic resolution we focused on.

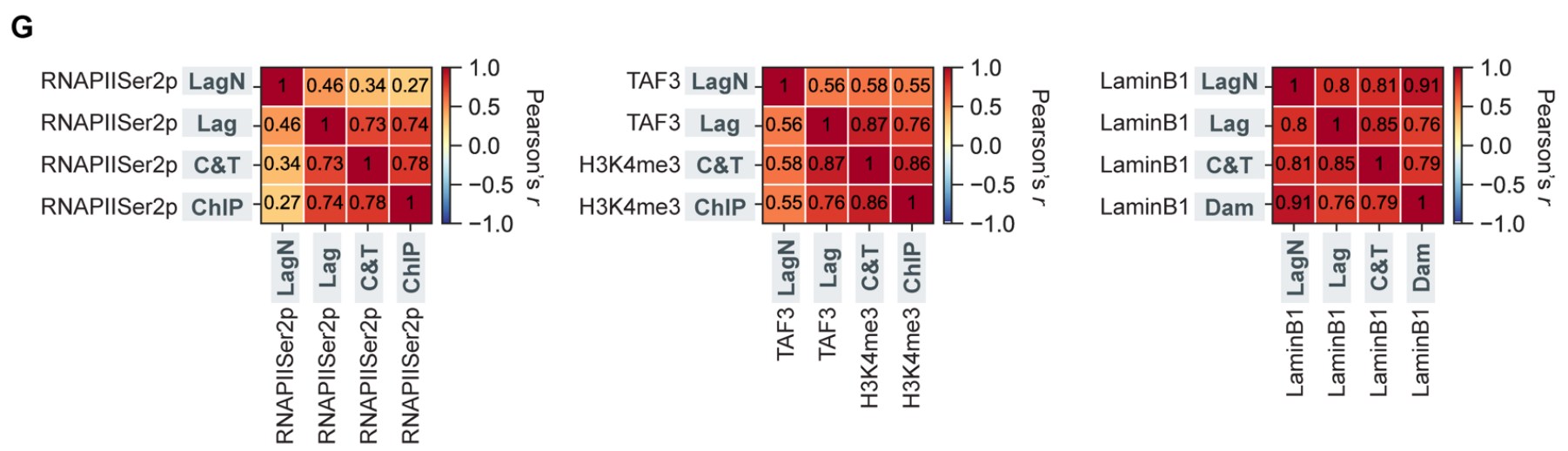

Figure S2G, Correlation heatmap comparing LagTag profiles and log2(target/mCherry)-normalized LagTag profiles against orthogonal datasets.

We note that, as the reviewer points out, this chromatin accessibility bias may pose a greater concern for low-abundance targets such as transcription factors, which were not profiled in this manuscript.

We have incorporated this analysis and the associated discussion into the STAR Methods section as described below.

"We note that in DamID assays, untethered methyltransferase preferentially labels accessible chromatin (Rooijers et al. 2019), and log2 ratio normalization over a Dam-only control is typically used to correct this bias (Vogel et al. 2007). We compared CPM and log2 ratio normalized LagTag profiles with orthogonal datasets. Log2 ratio normalization did not improve correlation with orthogonal profiles except for Lamin B1 pA-DamID, suggesting that open chromatin bias does not substantially confound LagTag enrichment signals at the 10-kb resolution used in this study. However, chromatin accessibility bias may pose a greater concern for low-abundance targets such as transcription factors, which were not tested here."

While LagTag enables recovery of earlier and endpoint chromatin states from the same cells, it remains important to clarify what types of mechanistic conclusions can and cannot be drawn from these data. Specifically, the method records occupancy history but does not directly resolve whether earlier chromatin states causally instruct subsequent states or merely correlate with them. A more explicit discussion of how LagTag distinguishes instructive epigenetic memory from passive persistence would strengthen the conceptual positioning of the approach. Demonstrating an example where LagTag reveals

a previously unobservable chromatin-fate relationship would strengthen the manuscript beyond proof-of-concept validation.

We thank the reviewer for this comment, which prompted us to clarify the advantages and limitations of LagTag. We have substantially revised the discussion section as detailed below. We believe that discovery-driven research at the interface of chromatin biology and cell fate represents an exciting future direction, and that this manuscript lays a critical foundation for such studies.

"The ability to recover earlier chromatin states, even from bulk populations, will help identify signatures that predispose cells to different outcomes or future trajectories in healthy and disease contexts. This is conceptually analogous to two-timepoint mRNA profiling, which has recently enabled transcriptional recording at the population level and identified molecular signatures of drug-naïve persister cells in a human cancer line (Chao et al. 2026)."

"While LagTag and other two-timepoint approaches alone do not establish direct causality between earlier chromatin states and subsequent cell fate transitions, they can reveal candidate molecular signatures and temporal ordering of events that are typically inaccessible from static, single time-point measurements (Weinreb et al. 2018; Kefalopoulou et al. 2025)."

Reviewer #2:

This manuscript reports LagTag, a method to simultaneously map two chromatin features: one as it is at the time of cell harvest, and one that was recorded earlier. It consists of a combination of MadID (to record and map the earlier feature) and CUT&Tag (to map the present feature). Proof-of-concept is demonstrated in a simple cell culture differentiation model.

The experiments are carefully designed and performed, and the figures are prepared with care. Clearly, a lot of work and dedication went into this. The text is also well-written and balanced.

Nevertheless, this study pales in comparison to the previous Dam&ChIC paper (Kefalopoulou, Nat Cell Biol 2025), which had a similar scope and m6A-based approach, but was much more exciting because Dam&ChIC yields recorded and present data from single cells. Furthermore, the current manuscript does not provide any new biological insights, unlike the Kefalopoulou study. Compared to Dam&ChIC, LagTag is probably less challenging to perform (as LagTag is not single-cell).

The authors argue that the MadID component of LagTag has an advantage over the scDamID component of Dam&ChIC, because scDamID in theory cannot record/detect a chromatin state that occurs prior to S-phase due to the inability of DpnI to cut hemi-m6A DNA. This is probably true. However, the current manuscript does not demonstrate conclusively that LagTag indeed has this longer "memory" (see below). It also does not explore the limits of this potential advantage of LagTag: over how many cell cycles would the "memory" be retained and detectable? This is relevant, because the DCM-TimeMachine method

(Boers, NatBiotech 2023; based on m5C) was reported to memorize earlier chromatin states over several cell cycles.

The authors mention that two other LagTag-like studies have been submitted by other labs, and it seems that they rushed out this manuscript because of this situation. I understand this pressure, but at present the manuscript - while technically sound - seems incomplete: (1) it lacks demonstration that interesting new biology can be discovered; (2) it does not compellingly demonstrate the extended memory ability compared to Dam&ChIC.

We thank the reviewer for recognizing our effort and providing feedback in comparison with other recent temporal methods. In the revised manuscript, we clarified that LagTag successfully recovered m6A signals after two cell cycles using two assays that either measure or estimate the number of cell divisions, a capability that was not demonstrated in prior Dam&ChIC work. In addition, to compare with related methods, including those suggested by the reviewer, we added a supplementary table (**Table S3**) to provide a comprehensive view of temporal chromatin recording technologies to readers.

In terms of biological discovery, we would like to note that the central goal of this manuscript is to present the LagTag technology to the scientific community with a rigorous proof-of-principle demonstration. This goal aligns with the aim of the Methods in Brief format in Cell Systems. We expect and hope that LagTag will enable us and others to make new biological discoveries in future projects.

Major points:

1. The results in Figure 2 are encouraging, but are strictly a proof-of-concept. The same maps could have been obtained by straightforward CUT&Tag on undifferentiated and differentiated cells (and probably more accurately), so there is no added value here. For cells in a dish, I could not think of an application where LagTag would provide data that cannot be obtained by simply harvesting cells before and after the stimulus that triggers their transition. It seems that LagTag may only have added value in more complex developmental biology settings? I realize it would be a lot of work, but demonstration of true added biological value - perhaps in gastruloids - would substantially strengthen this manuscript.

We agree with the reviewer that the current demonstration of LagTag in this manuscript is strictly a proof-of-concept. However, even in cell culture settings, LagTag could serve as a powerful tool for discovery-driven research. For example, a recent two-timepoint transcriptomic profiling study from the same samples at bulk resolution (Chao et al., 2026, PMID: 41538410) demonstrated the discovery of molecular signatures of drug-naïve persister cells in a human cancer line, which is precisely the type of question LagTag is well-positioned to address at the chromatin level. While extending LagTag to new biological systems, such as gastruloids, is beyond the scope of the current manuscript, we envision such applications as exciting future directions. To reflect these points, we have revised the discussion as follows.

"The ability to recover earlier chromatin states, even from bulk populations, will help identify signatures that predispose cells to different outcomes or future trajectories in healthy and disease contexts. This is

conceptually analogous to two-timepoint mRNA profiling, which has recently enabled transcriptional recording at the population level and identified molecular signatures of drug-naïve persister cells in a human cancer line (Chao et al. 2026).”

2. Figure 2d+e are encouraging, but are anecdotal. I found Figure 2f very difficult to interpret, largely due to the z-score transformation that completely obscures fold-change effect sizes. I was also confused why the clustering of the genes in the left and right panels was not kept the same.
Importantly, I am missing more rigorous benchmarking of the recorded signal. I suggest a simple scatterplot that shows, for all genes, the fold-change between present and recorded according to LagTag versus the fold-change between differentiated and undifferentiated cells according to conventional CUT&Tag. Ideally, this would yield a plot with most genes close to the diagonal. But I am open to other systematic quantitative benchmarking, for example estimates of false-positives and false-negatives in a binarised setting.

We thank the reviewer for the suggestion to improve the visual clarity and rigor of the results.

Regarding **Figure 2F**, we have sorted the genes in both panels using the same order, based on the clustering in the left panel, in the revised manuscript. We had also provided side-by-side comparisons of enrichment from the two antibodies for each condition in **Figure S9F** of the original manuscript. We agree that the z-score scaling centers each gene's signal and obscures absolute fold-change magnitudes, which is a known trade-off. However, the z-score scaling is standard for this type of clustering-based visualization, as seen in DCM-TM (e.g., Fig. 3h) and other chromatin profiling studies (e.g., Llorens-Bobadilla et al., 2023, PMID: 36604544). To address this concern, we have also added fold-change comparisons as detailed in the next paragraph.

F

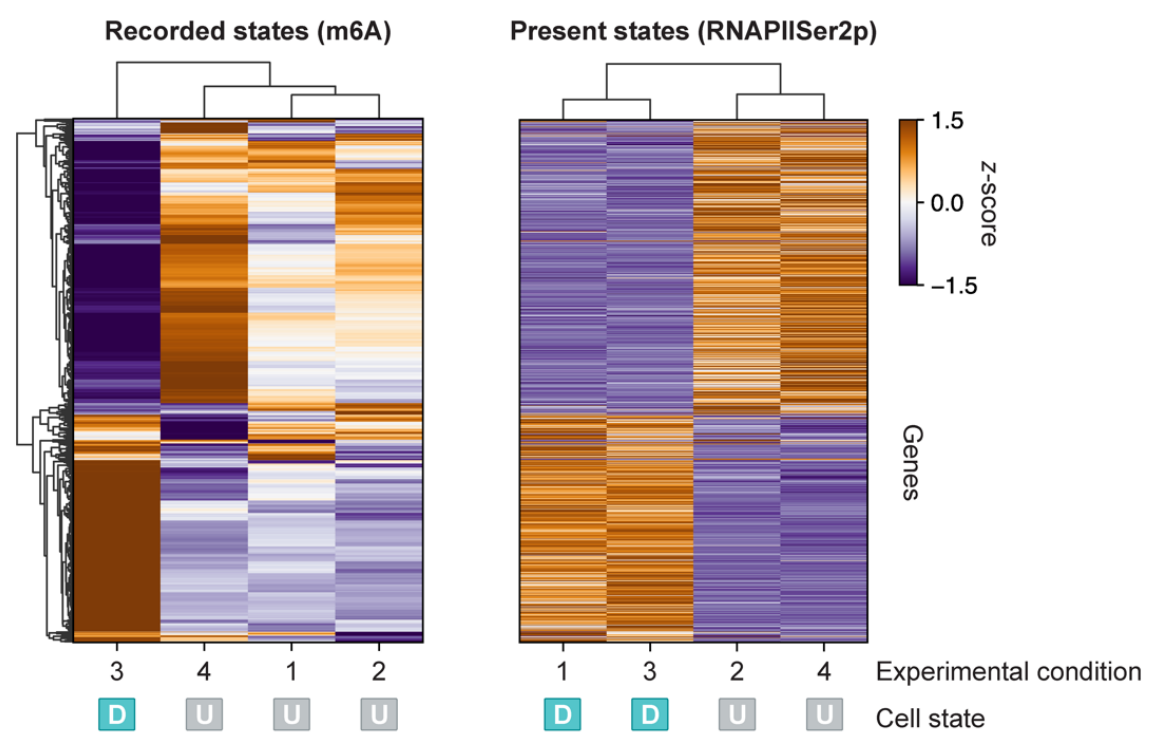

Figure 2F**, Cluster maps of genes and conditions for recorded (left) and present (right) chromatin states using the differentially enriched genes with RNAPIISer2p (n = 933 genes) between undifferentiated and differentiated cell states, defined by separate CUT&Tag experiments (n = 3).* ***Gene ordering in the right panel follows the clustering in the left panel.

To benchmark the fold change between differentiated and undifferentiated cells measured by LagTag against that measured by conventional CUT&Tag, we added scatter plots (**Figure S9G, H**). We observed a positive correlation between the two measurements, with a Pearson correlation coefficient of 0.65 for all coding genes (**Figure S9G**). We noticed that the correlation coefficient decreases to 0.58 when non-coding genes are included (**Figure S9H**). We found that this discrepancy is mainly driven by changes in short genes, such as miRNAs. Consistently, a similar loss of signal for short genes was also observed when comparing RNAPIISer2p performed together with m6A antibody against conventional RNAPIISer2p CUT&Tag, suggesting that this is not specific to LagTag. We speculate that these fold-change discrepancies for short genes arise from the NaOH denaturation step introduced in the assays to map m6A signals. We have incorporated this new analysis and a discussion of this potential limitation in the revised manuscript.

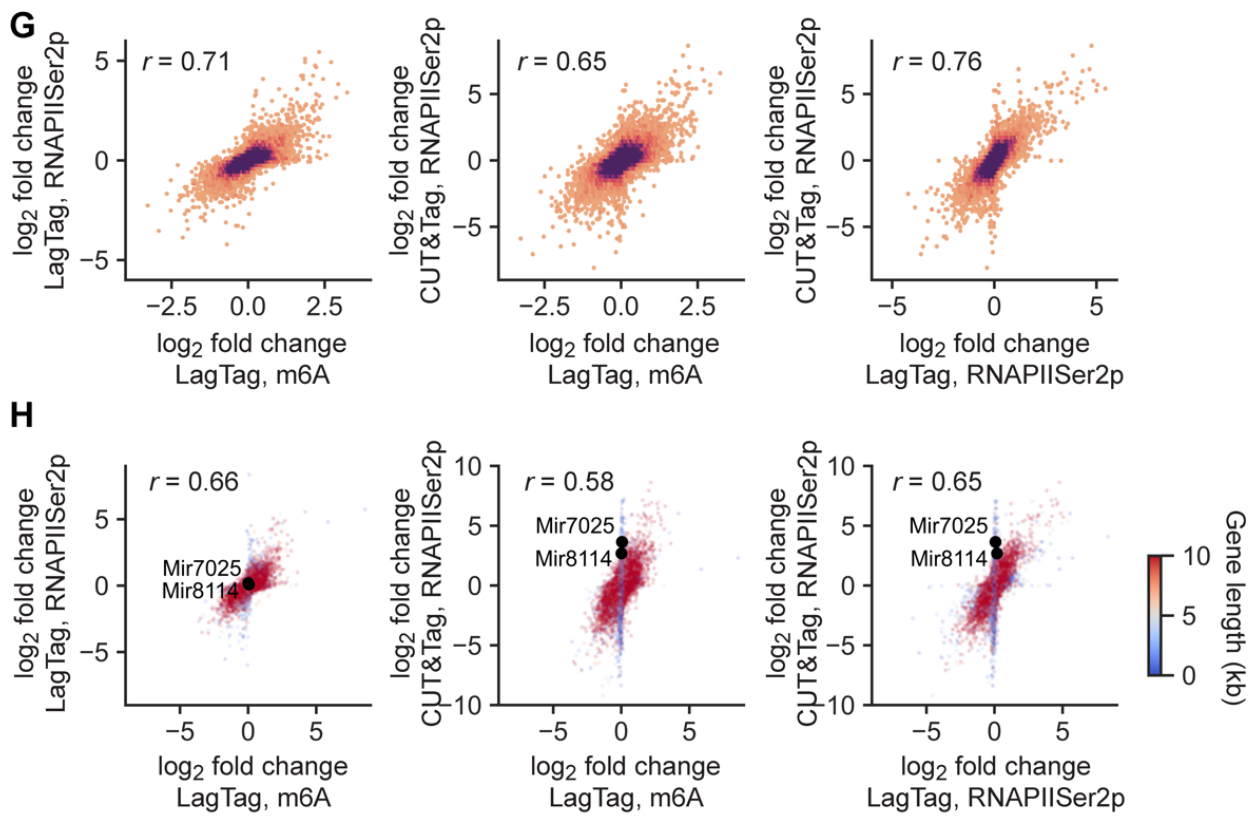

Figure S9G, H, Fold change in RNAPIISer2p signal enrichment over gene bodies during differentiation measured by LagTag (m6A or RNAPIISer2p antibody in condition 3 vs condition 4) and CUT&Tag (RNAPIISer2p only in condition 5 vs 6) in engineered mESC-Gata4 cells for coding genes (G) and all genes (H). Fold changes for some short genes were not captured by LagTag with NaOH denaturation compared with conventional CUT&Tag, potentially limiting the detection of a subset of genes by LagTag. Two representative short genes (Mir7025, Mir8114) are highlighted.

3. The data from the ESC->endoderm differentiation model are interpreted as demonstration that m6A-memory is retained over mitosis/S-phase. However, I could not find evidence that the cells continue to cycle when differentiation is trigged by GATA4 induction. Did the harvested cells really go through S-phase during the "chase" period?

We have added new experimental results and analyses to estimate the number of cell divisions occurring during the chase period by counting viable cells over time (**Figures 2G and S10B**). We showed that during differentiation, the recorded cells divided approximately twice (2.0 ± 0.1 and 1.9 ± 0.2 divisions, respectively with mean ± standard deviation, for undifferentiated and differentiated cells, respectively) within our 24-hour chase period by direct measurement (**Figure S10B**). In addition, we leveraged the distribution of read count ratio between m6A and RNAPIISer2p across experimental conditions to independently estimate the number of cell divisions (**Figure 2G**). Specifically, we assumed that the

RNAPIISer2p level remains constant while the m6A level varies depending on pulse conditions. Using a cell division dilution model, we recovered an estimated division count of approximately two (2.2 ± 0.1 and 1.7 ± 0.3 divisions, for undifferentiated and differentiated cells, respectively) within 24 hours. These estimates are consistent with reported cell cycle times of undifferentiated (13.25 hours) and early-differentiated (10.5 hours) cells by live-cell imaging (Waisman et al., 2019, PMID: 31142785). Taken together, multiple lines of evidence support the conclusion that cells underwent approximately two divisions under our experimental conditions.

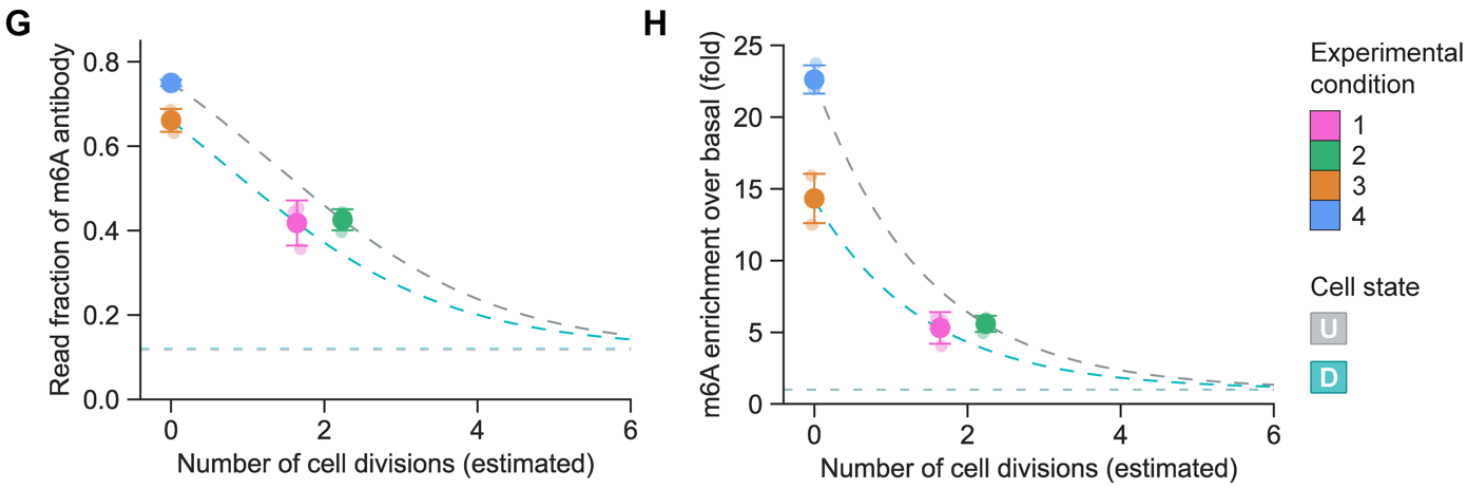

Figure 2G, H, Read fraction of the m6A antibody (G) or m6A fold enrichment over basal (H) as a function of estimated cell divisions, modeled using a cell division dilution framework (see STAR Methods). Error bars represent mean ± standard deviations with individual replicate points overlaid. Results are shown separately for undifferentiated and differentiated cell states. Basal levels (dashed asymptotes) were determined from uninduced controls (conditions 5 and 6). Dashed curves show the predicted decay over cell divisions, derived from the measured basal and induced conditions (see Figure S9B).

B

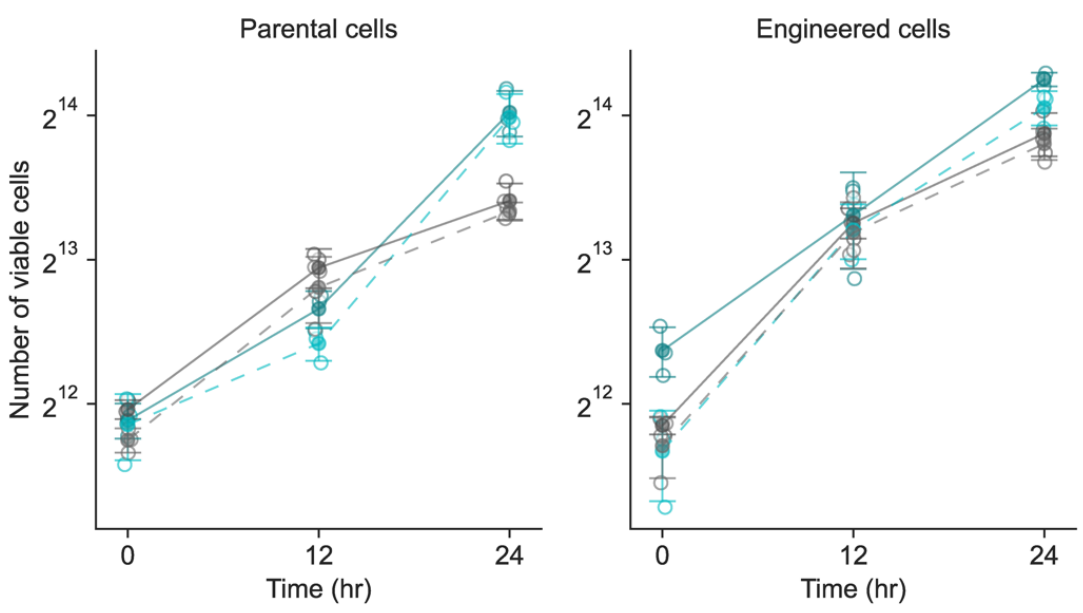

Figure S10B, Comparison of number of viable cells with or without prior Shield-1 induction for 16 hours in undifferentiated and differentiated cells (n = 3).

We have incorporated the new results, analyses, and discussion as follows.

"During this time, cells underwent approximately two cell divisions (2.1 ± 0.3 and 1.9 ± 0.2 divisions, mean ± standard deviation, for parental and engineered cells, respectively) (Figure S10A, B). This is consistent with cell cycle times of 13.25 hours (undifferentiated) and 10.5 hours (early-differentiated) reported by live-cell imaging"

4. Over how many cell cycles can a pattern be memorized and recovered? It should be fairly straightforward to test this in the cell differentiation model.

We appreciate this point. We have added a cell division dilution model describing the relationship between the sequencing read fraction of m6A and the number of cell divisions, based on the experimental data (**Figure 2G, H**), as described in the response above. Using this model, we found that the m6A fold enrichment over basal decays from 14.2, 4.3, 1.8, to 1.2 for 0, 2, 4, and 6 cell divisions in differentiated cells, respectively, where the values are extrapolated from the model except for the no-division starting point. These estimates indicate that LagTag can reliably recover recorded chromatin information after two cell divisions, as demonstrated in this manuscript. However, the signal becomes indistinguishable from background after 6 cell divisions. To clarify these points, we have revised the main text as follows.

"Finally, we estimated the m6A signal decay as a function of cell divisions (Figure 2G, H) by leveraging the read count ratio between m6A and RNAPIISer2p across conditions in combination with a cell division dilution model (see STAR Methods). Based on this analysis, we estimate approximately two cell divisions elapsed for undifferentiated and differentiated cells (2.2 ± 0.1 and 1.7 ± 0.3 with mean ± standard deviation, respectively) during the 24-hour chase period, consistent with the direct results from the cell division assay (2.0 ± 0.1 and 1.9 ± 0.2 divisions, respectively) (Figure S10B). This model further defined an effective window of approximately two or three cell divisions, within which m6A signal enrichment remains robust (e.g., 14.2 vs. 4.3 fold m6A signal enrichment over basal comparing no-chase and 2-division chase in differentiated cells), before progressively approaching background levels after approximately six cell divisions (Figure 2G, H). These results establish that LagTag can retrospectively profile chromatin states across at least two cell divisions in proliferating cells."

=============== Reviewed by Bas van Steensel. Review task accepted: 2 March 2026; completed: 12 March 2026. It is my standard policy to disclose my identity on *all* my review reports, regardless of my comments and recommendations. All correspondence should go via the editor. PLEASE DO NOT REMOVE THIS NOTE. =========

Reviewer #3: In this study, Takei et al. introduce a very elegant approach to obtain snapshots of molecular events that occur in cells as a function of time jointly with their associated chromatin state. By using an adenine DNA methyltransferase that is found in bacteria, the authors could achieve high efficiency labeling of DNA and at the same time perform CUT and Tag for any epitope of interest. This is a method that is very likely to have broad interest for the chromatin community and those interested in obtaining temporal information about cellular states. The optimizations of particularly challenging experimental steps to ensure that the authors obtain high quality data is especially commendable. The authors also pointed out parallel publications using a similar approach and it clearly is a testament to the strength of such recording approaches and highlights an important direction for the field moving forward.

We thank the reviewer for recognizing the importance of our work in the field and providing constructive comments. Based on the reviewer's comments, we have improved the readability and clarity of our manuscript as detailed below.

Major Comments:

1) The main issue I have with the paper is how dense the structure and text is. Clearly this is a paper written for a journal format that doesn't offer flexibility for page length which makes following the logic, the figures and the outcomes of the experiment very challenging. Personally, I felt that it does significantly undersells the very elegant and truly exceptional data that this manuscript presents. Splitting figures and using the text to lay out the arguments more clearly would substantially improve readability.

We appreciate this point and agree. In the revision, we have expanded the text to ensure that the key points come through more clearly. In particular, we have emphasized upfront that Figure 1 presents the establishment of the new technology foundation and Figure 2 presents the proof-of-principle experiment as described below. In addition, we have clarified the terminology used at the beginning in the revised manuscript (see more in the response to comment 5).

*"We first establish the technical foundation of LagTag (**Figure 1**) and then present proof-of-principle experiments demonstrating that temporal chromatin recording captures earlier chromatin signatures during mouse embryonic stem cell differentiation to endodermal fates (**Figure 2**)."*

2) There is one major conceptual point I do not fully understand: what is the value of marking the initial chromatin state with DNA methylation, given that this state can be fully characterized simply as whatever exists at t = 0? Since the methylation pattern does not evolve spatially over time, beyond a slow decay it tells us only how things began, while the chromatin state itself must still convey how things are changing. It is true that CUT&Tag does not record prior events, but in this implementation, methylation appears functionally equivalent to CUT&Tag performed at t = 0.
To be more specific: in Figure 1h, a plot is shown with "recorded" and "endpoint" measurements. Is the primary purpose of this figure to demonstrate joint profiling of DNA methylation and histone modifications? If so, it is not clear to me where the temporal component enters the scheme. I can only assume the main payoff will come when this method is adapted to a single-cell format, but I would appreciate clarification on this point.

The main purpose of LagTag is to extract two-timepoint chromatin information from the same sample. Figure 1H demonstrates joint profiling of m6A and RNAPIISer2p from the same cells. The temporal component is that the m6A signal reflects the integral of chromatin state over the preceding 16 hours of Shield-1 induction. However, because the cells were at steady state in this experiment, both m6A and RNAPIISer2p markers reflect the same undifferentiated mESC state. To clarify this point, we have changed the term "recorded" to "m6A-recorded" in this section. The differentiation experiments in Figure 2 demonstrate the recovery of distinct cell states from the same samples, capturing the m6A-recorded undifferentiated state and the RNAPIISer2p-captured differentiated state simultaneously.

As the reviewer points out, the full payoff of LagTag will be realized in the single-cell format and the possibility of single-cell version was discussed in the original manuscript. Nevertheless, we believe that

bulk recovery of past chromatin states itself has utility for discovery-driven purposes. We have now clarified these points in the discussion section of the revised manuscript as follows.

"The ability to recover earlier chromatin states, even from bulk populations, will help identify signatures that predispose cells to different outcomes or future trajectories in healthy and disease contexts. This is conceptually analogous to two-timepoint mRNA profiling, which has recently enabled transcriptional recording at the population level and identified molecular signatures of drug-naïve persister cells in a human cancer line (Chao et al. 2026)."

3) Have the authors generated data in which methylation exhibits a decay over time? If so, this would be valuable to highlight in the main figures, along with a characterization of the dilution rate, to provide an estimate of how long the signal lasts. Sorry if the data is in the manuscript and I missed it.

We appreciate this point. We leveraged the distribution of read count ratio between m6A and RNAPIISer2p across experimental conditions to estimate the decay of the recorded m6A sequencing read fraction as a function of cell divisions (**Figure 2G, H**) in the revised manuscript. Specifically, we assumed that the RNAPIISer2p level remains constant while the m6A level varies depending on pulse conditions. Using a cell division dilution model, we found that the m6A fold enrichment over basal decays from 14.2, 4.3, 1.8, to 1.2 for 0, 2, 4, and 6 cell divisions in differentiated cells, respectively, where the values are extrapolated from the model except for the no-division starting point. These estimates indicate that LagTag can reliably recover recorded chromatin information after two cell divisions, as demonstrated in this manuscript. However, the signal becomes indistinguishable from background after 6 cell divisions. To clarify these points, we have revised the main text as follows.

"Finally, we estimated the m6A signal decay as a function of cell divisions (Figure 2G, H) by leveraging the read count ratio between m6A and RNAPIISer2p across conditions in combination with a cell division dilution model (see STAR Methods). Based on this analysis, we estimate approximately two cell divisions elapsed for undifferentiated and differentiated cells (2.2 ± 0.1 and 1.7 ± 0.3 with mean ± standard deviation, respectively) during the 24-hour chase period, consistent with the direct results from the cell division assay (2.0 ± 0.1 and 1.9 ± 0.2 divisions, respectively) (Figure S10B). This model further defined an effective window of approximately two or three cell divisions, within which m6A signal enrichment remains robust (e.g., 14.2 vs. 4.3 fold m6A signal enrichment over basal comparing no-chase and 2-division chase in differentiated cells), before progressively approaching background levels after approximately six cell divisions (Figure 2G, H). These results establish that LagTag can retrospectively profile chromatin states across at least two cell divisions in proliferating cells."

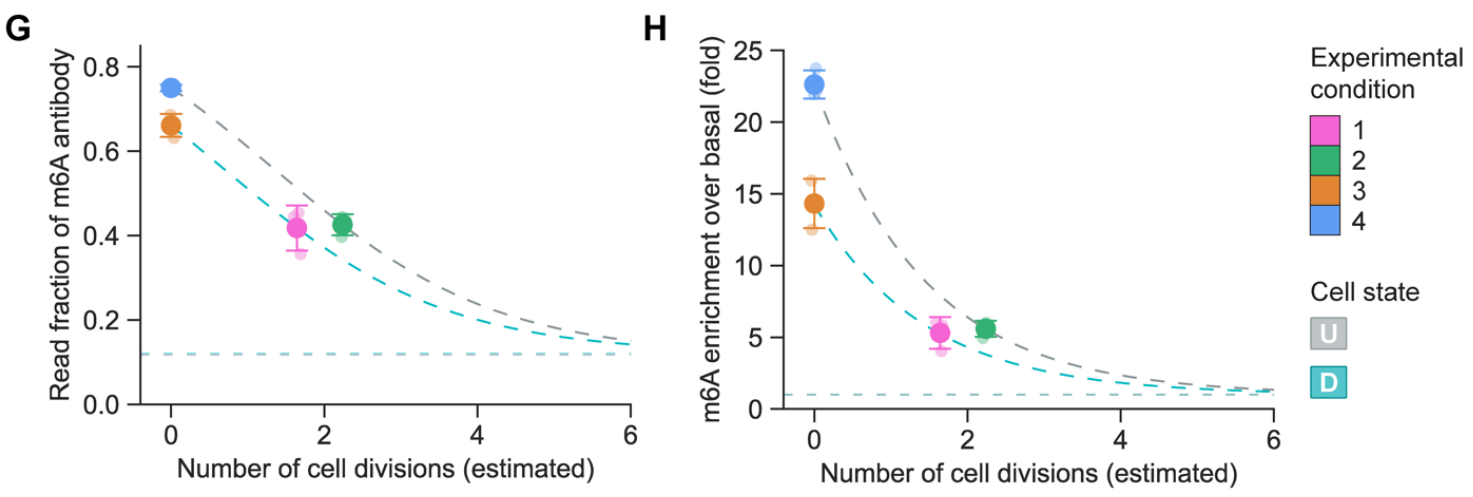

Figure 2G, H, Read fraction of the m6A antibody (G) or m6A fold enrichment over basal (H) as a function of estimated cell divisions, modeled using a cell division dilution framework (see STAR Methods). Error bars represent mean ± standard deviations with individual replicate points overlaid. Results are shown separately for undifferentiated and differentiated cell states. Basal levels (dashed asymptotes) were determined from uninduced controls (conditions 5 and 6). Dashed curves show the predicted decay over cell divisions, derived from the measured basal and induced conditions (see Figure S9B).

4) Relatedly, how short can the labeling pulse be? The labeling times reported here are quite long, likely reflecting limitations in enzyme activity or abundance, both of which presumably require careful titration to prevent non-specific labeling.

We have added new results comparing the effects of different labeling pulse durations on m6A signal enrichment (**Figure S3C**). We observed a substantial increase in m6A enrichment at 16 hours compared to 8 hours of pulse duration, indicating that LagTag requires a labeling window longer than 8 hours. We also showed that LagTag labeling time is similar to or shorter than the other temporal chromatin recording methods, which typically require labeling durations of days (**Table S3**).

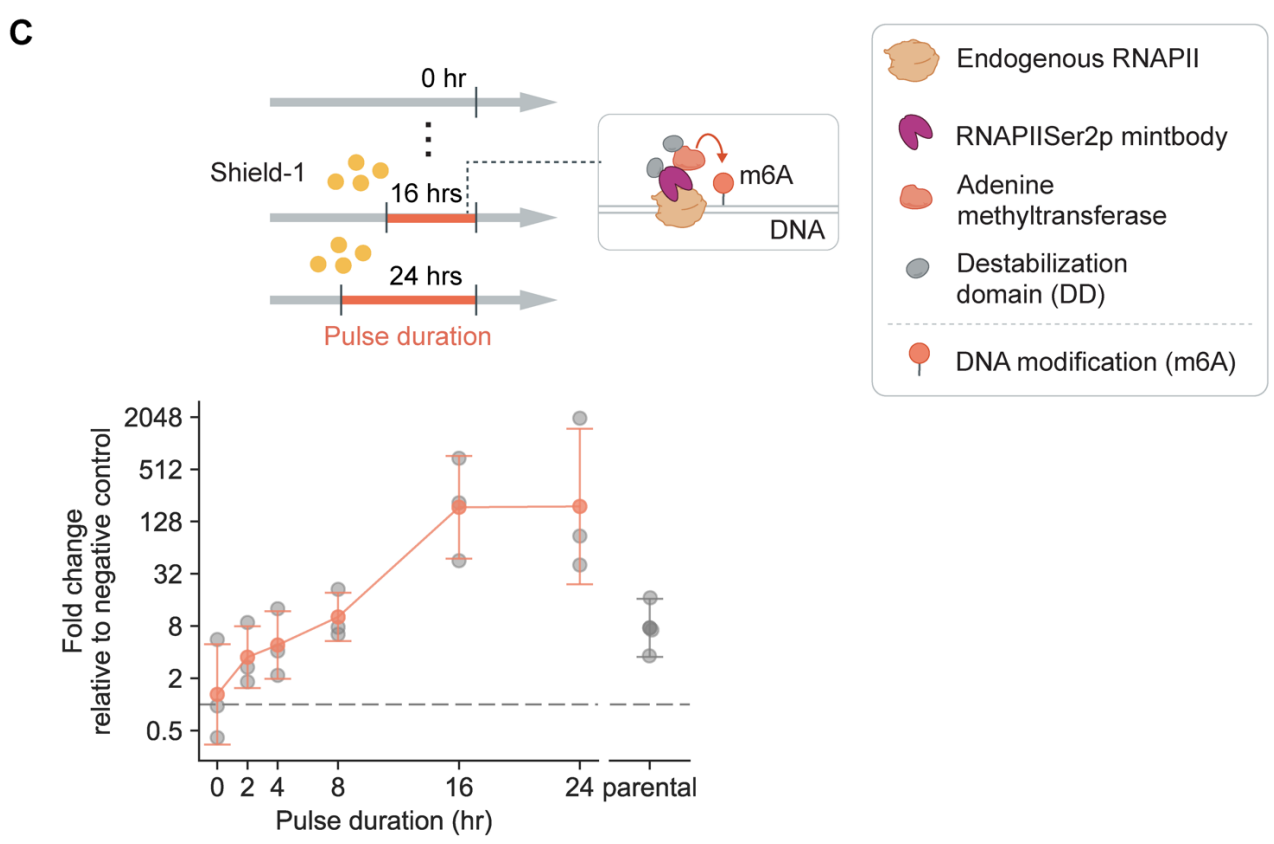

Figure S3C, Comparison of LagTag yield as a function of Shield-1 pulse duration. *Comparison of LagTag yield as a function of Shield-1 pulse duration. Fold change was calculated relative to primer-only controls by qPCR (see STAR Methods). We note that the m6A signal deposited by LagTag represents a temporal integral of chromatin states during the pulse duration, consistent with biophysical modeling of adenine methyltransferase kinetics (Redolfi et al. 2019). The LagTag pulse duration is similar to or shorter than the other temporal chromatin methods (**Table S3**). The DD-M.EcoGII-Ser2p-mintbody-DD-expressing mESCs*

and parental mESCs (control) were used for this assay. Error bars represent mean ± standard deviation with individual replicate data points overlaid (n = 3).

5) Since these experiments are performed on cell populations rather than individual cells, the claim that LagTag enables observation of the same cell at different time points appears to be incorrect.

We agree with the reviewer that this distinction is important to clarify. We have revised the manuscript to reflect that current LagTag experiments are performed on cell populations rather than individual cells. For example, we have replaced "same cells" with "same cell population" in the abstract, as shown below.

"LagTag was able to record and recover dynamic chromatin state transitions during mouse embryonic stem cell differentiation, capturing transcriptional signatures from pre- and post-differentiation timepoints within the ***same cell population****."*

Terminology

I found the terminology in the manuscript very hard to follow. Is recorded the same as pulse labeling with methylation? Given its tense, is recording not a continuous process? how is it different from using the well-established and widely used terminology of pulse and chase? It seems to me that pulse and chase are a lot more straightforward unless the recording has some special implication, but this was also not clear. Also, LagTag is interchangeably used with many of these words in the manuscript and I couldn't figure out which part of the protocol is recording, end point and LagTag. Then to make matters worse, there are two other terms past and present?

We thank the reviewer for the helpful comments. We have clarified the terminology in the revised manuscript by adding the description at the beginning of the LagTag introduction. In particular, "recording" is used here in a conceptual sense distinct from pulse-chase. While the experimental procedure follows a standard pulse-chase design, the key feature of LagTag is that the m6A mark deposited during the pulse is stably retained in chromatin and retrievable at a later endpoint, analogous to a recording that can be played back after the event. We agree that this distinction was not sufficiently clear. We have added clarifying sentences on the use of "recording" to explicitly connect it to the pulse-chase framework. The term "LagTag" refers specifically to the overall technology, while "recording" describes the conceptual operation it performs. We have also clarified the "past" and "present" terminology based on the timing of the recording.

"Here we present LagTag, a technology that records chromatin states by pulse-labeling with m6A and recovers them alongside additional chromatin features by simultaneous readout at a later endpoint. m6A recording performed immediately before the endpoint captures the present chromatin states, similarly to conventional DamID approaches, whereas a subsequent chase period after recording enables retrospective profiling of past chromatin states across cell divisions."

Responses to reviewers' comments:

We thank the reviewers for their comments and suggestions to improve our manuscript. Our point-by-point responses are provided below in blue text.

Reviewer comments:

Reviewer #1: Thank you for the revised manuscript. The additional controls are appreciated and address most of my concerns. However, one issue remains unresolved and I cannot recommend acceptance without it being addressed.

This is a methods paper. The core claim is that LagTag offers meaningful advantages over existing approaches. Table S3 lists parameters from published papers across different labs and systems: that is not benchmarking, that is literature summary. To support the central claim, the authors need to actually run LagTag and Dam&ChIC on the same biological system and show the comparison. I am not asking for an exhaustive analysis - one focused experiment would suffice.

The argument that the Methods in Brief format justifies skipping this is not convincing. The format may excuse the absence of new biological discovery, but it does not excuse the absence of evidence that the method offers advantages over what already exists.

I am recommending major revision. The work is technically solid and I want to see it published, but not before this gap is filled.

We thank the reviewer for this suggestion. We agree such comparisons would be informative; however, we have not been able to perform additional direct experimental comparisons at this time.

Reviewer #3: I appreciate the authors of the manuscript taking note of my comments and addressing them. The language in the paper is much improved, and I feel the ambiguities around how the experiment is conducted are better resolved. The dats is much clearer and easier to process (understand)

There are two key experimental pitfalls that the authors highlight in their response that I want to call attention to. I am not saying these are reasons to make decisions about the manuscript but it would be very valuable to be more explicit about them as limitations. The first concerns applying the method to single cells which is really the payoff for a method such as LagTag. This is a limitation of the current study and remains to be seen in future work as to whether this method will be applicable in the contexts they propose. The second concerns labeling time which is 16 hours. I appreciate the point that it is comparable to other methods but it is still long especially given that that some embryonic cell types divide faster than the labeling time which would dilute signal and potentially increase the labeling time window. My recommendation is still to include a section in the discussion that highlights these pitfalls more expcitly so that users of the approach know what is and isn't working.

We thank the reviewer for this comment. We have included an extended discussion addressing this point in the revised manuscript.
