# Supplementary material for: Genome-wide chromatin recording resolves dynamic cell state changes
Source: Cell Syst. Author manuscript; Available in PMC 2026 Sep 3. (PMC13540299; doi:10.1016/j.cels.2026.101717)
Supplement: Supplementary file 2 [file NIHMS2204262-supplement-2.pdf]

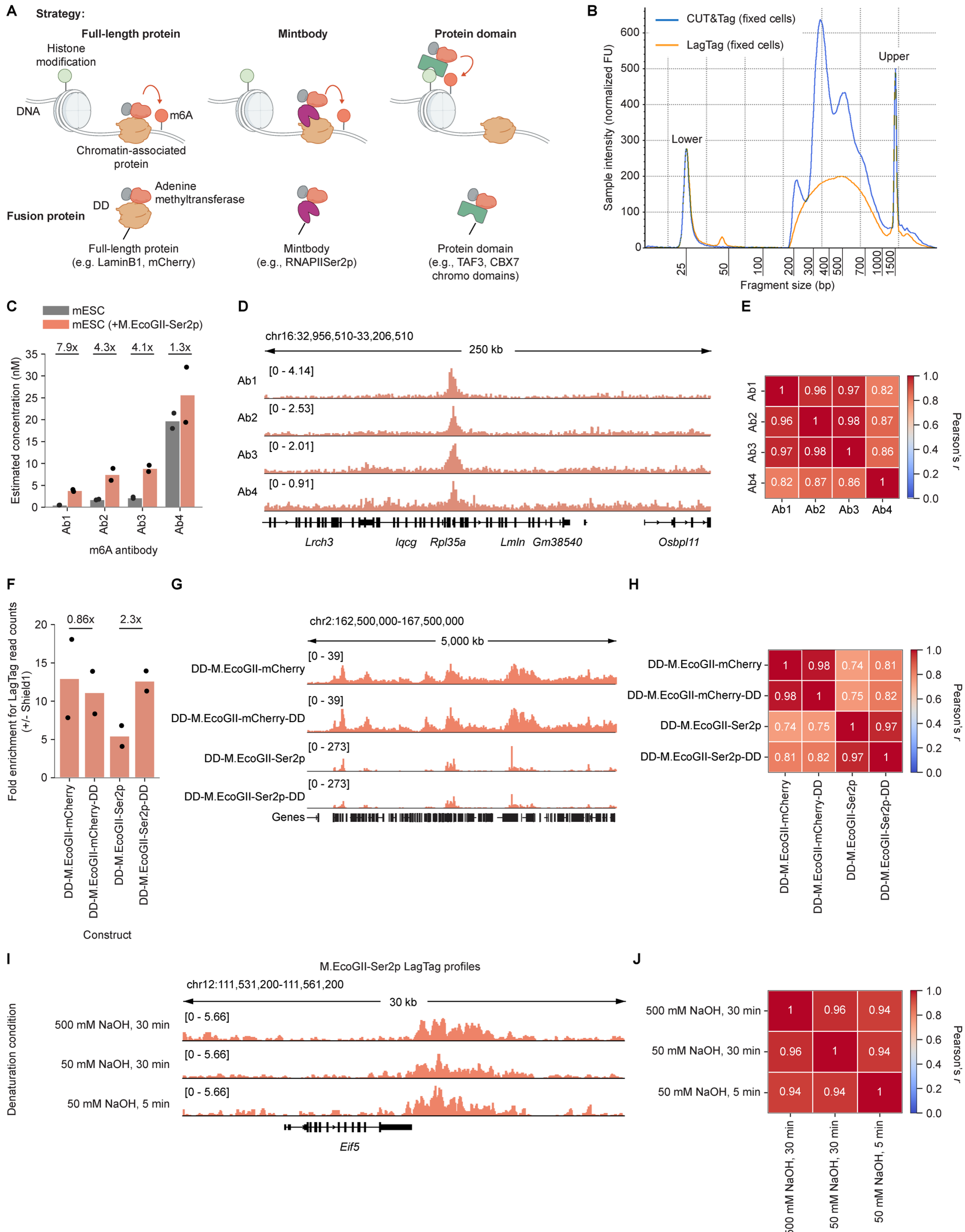

Figure S1: **Optimization of CUT&Tag and LagTag for fixed cells**

A, Schematic overview of different targeting strategies used for LagTag. **B**, Representative Tapestation profiles for LagTag and CUT&Tag in fixed cells. The profiles were obtained from RNAPIISer2p in mESCs. We note that the optimized condition of using proteinase K during the elution step, introduced by FFPE CUTAC[1] for heavily crosslinked samples, also improves the yield of CUT&Tag and LagTag products from fixed cells by 1% formaldehyde directly on 96-well plates (see **STAR Methods**). **C-E**, Comparison of different m6A antibodies (**Table S1**) with the LagTag assay in wild-type and M.EcoGII-Ser2p-mintbody-expressing mESCs. Ab1 was selected as a default m6A antibody based on its dynamic range. The yields of PCR products were estimated by the tapestation profiles within 200-1,000 bp in **C**. **F-H**, Comparison of different M.EcoGII fusion protein constructs, including different numbers of DD, with the LagTag assay in mESCs. The fold-enrichment with the addition of Shield-1 was calculated by comparing the read counts of samples mixed after PCR. **I**, **J**, Comparison of LagTag profiles across different denaturation conditions using M.EcoGII-Ser2p-mintbody-expressing mESCs. We note that the most harsh treatment condition of 500 mM NaOH for 30 minutes was used in the immunofluorescence assay in MadID[2]. Scale of LagTag profiles was normalized to coverage per million aligned reads in **D**, **G**, **I**. Correlation heatmaps of LagTag profiles were computed by using 10-kb bins across the genome in mESCs in **E**, **H**, **J**. n = 2 for experiments in **C**-**J**.

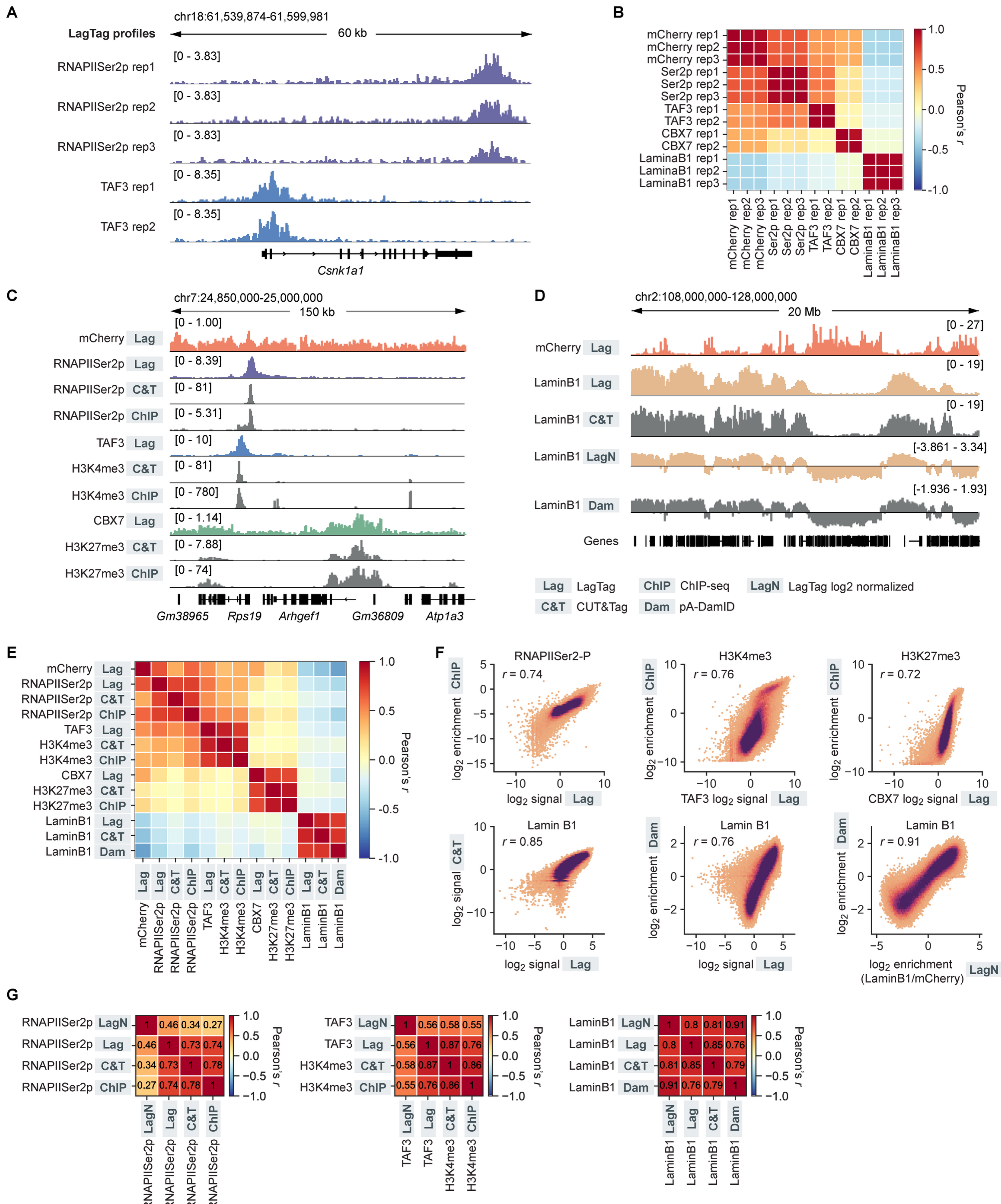

Figure S2: **Validation and characterization of LagTag profiles in mESCs**

A, **B**, Comparison of LagTag profiles between well replicates in mESCs. 10-kb bins were used for comparison in **B**. **C**, **D**, Genome browser tracks of LagTag, CUT&Tag, ChIP-seq[3,4], and pA-DamID[5]. Scale of LagTag and CUT&Tag profiles was normalized to coverage per million aligned reads or normalized to

log2 ratio between Lamin B1 and mCherry profiles (Lamin B1 LagTag normalized in **D**). The ChIP-seq profiles are input normalized and pA-DamID profiles are normalized by the Dam only control. **E**, Correlation heatmaps of chromatin profiles for different targets as well as different profiling methods using 10-kb bins across the genome in mESCs. **F**, Genome-wide comparison of chromatin profiles by different methods. r represents Pearson correlation coefficient shown in **E**. **G**, Correlation heatmap comparing LagTag profiles and log2(target/mCherry)-normalized LagTag profiles against orthogonal datasets. n = 2 for TAF3 and CBX7, and n = 3 for mCherry, RNAPIISer2p, and Lamin B1 in LagTag experiments; n = 2 for CUT&Tag experiments.

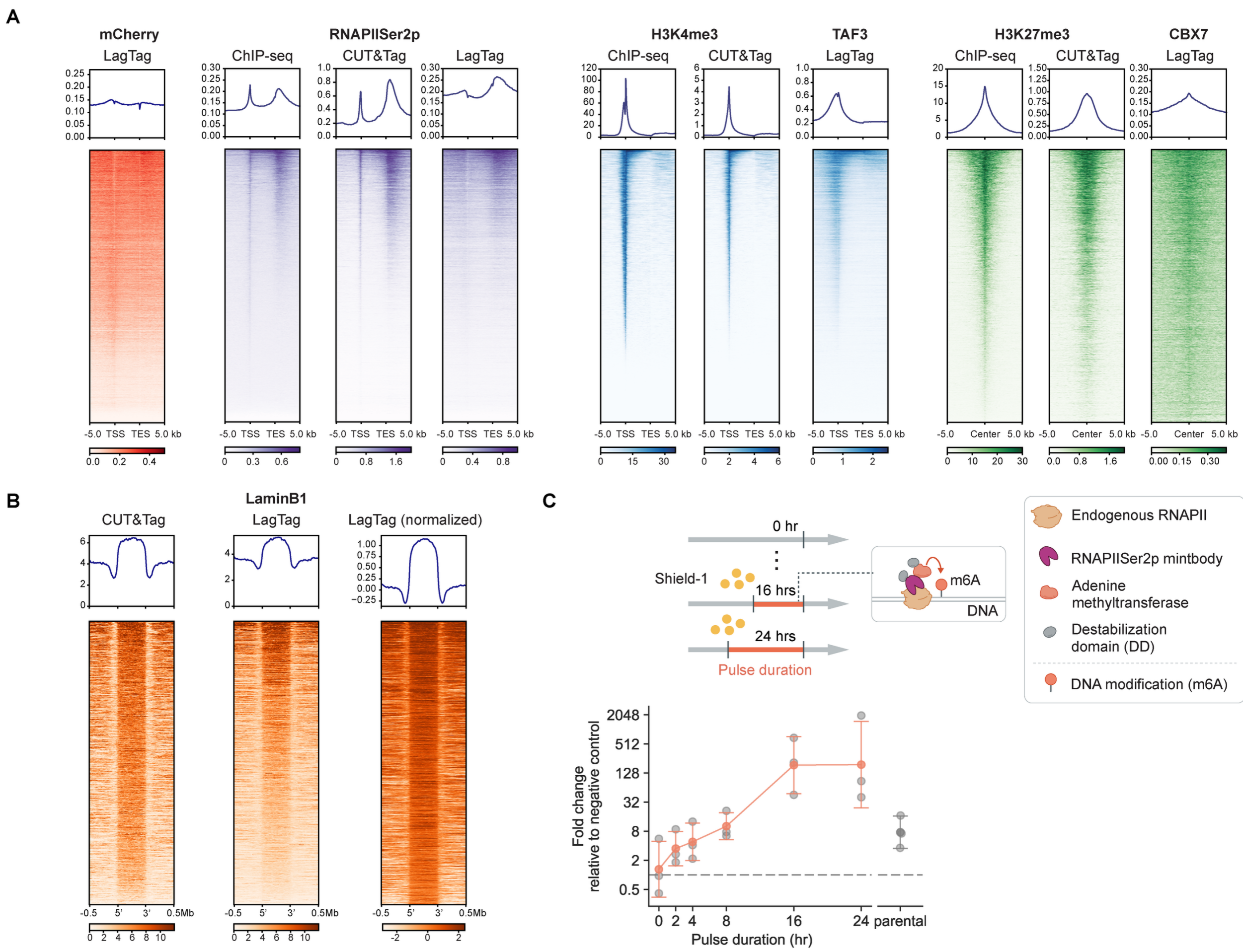

Figure S3: **Additional Validation and characterization of LagTag profiles in mESCs**

A, **B**, Signal enrichment plots for ChIP-seq[3,4], CUT&Tag, and LagTag around TSS and TES across all protein-coding genes for mCherry, RNAPIISer2p, H3K4me3, or around called peaks from ChIP-seq[6,7] for H3K27me3, or around LADs defined by DamID[8] for Lamin B1 in mESCs. Scale of LagTag and CUT&Tag profiles was normalized to coverage per million aligned reads or normalized to log2 ratio between Lamin B1 and mCherry profiles (Lamin B1 LagTag normalized in **B**). n = 2 for TAF3 and CBX7, and n = 3 for mCherry, RNAPIISer2p, and Lamin B1 in LagTag experiments; n = 2 for CUT&Tag experiments. **C**, Comparison of LagTag yield as a function of Shield-1 pulse duration. Fold change was calculated relative to primer-only controls by qPCR (see **STAR Methods**). We note that the m6A signal deposited by LagTag represents a temporal integral of chromatin states during the pulse duration, consistent with biophysical modeling of adenine methyltransferase kinetics[9]. The LagTag pulse duration is similar to or shorter than the other temporal chromatin methods (**Table S3**). The DD-M.EcoGII-Ser2p-mintbody-DD-expressing mESCs and parental mESCs (control) were used for this assay. Error bars represent mean ± standard deviation with individual replicate data points overlaid (n = 3).

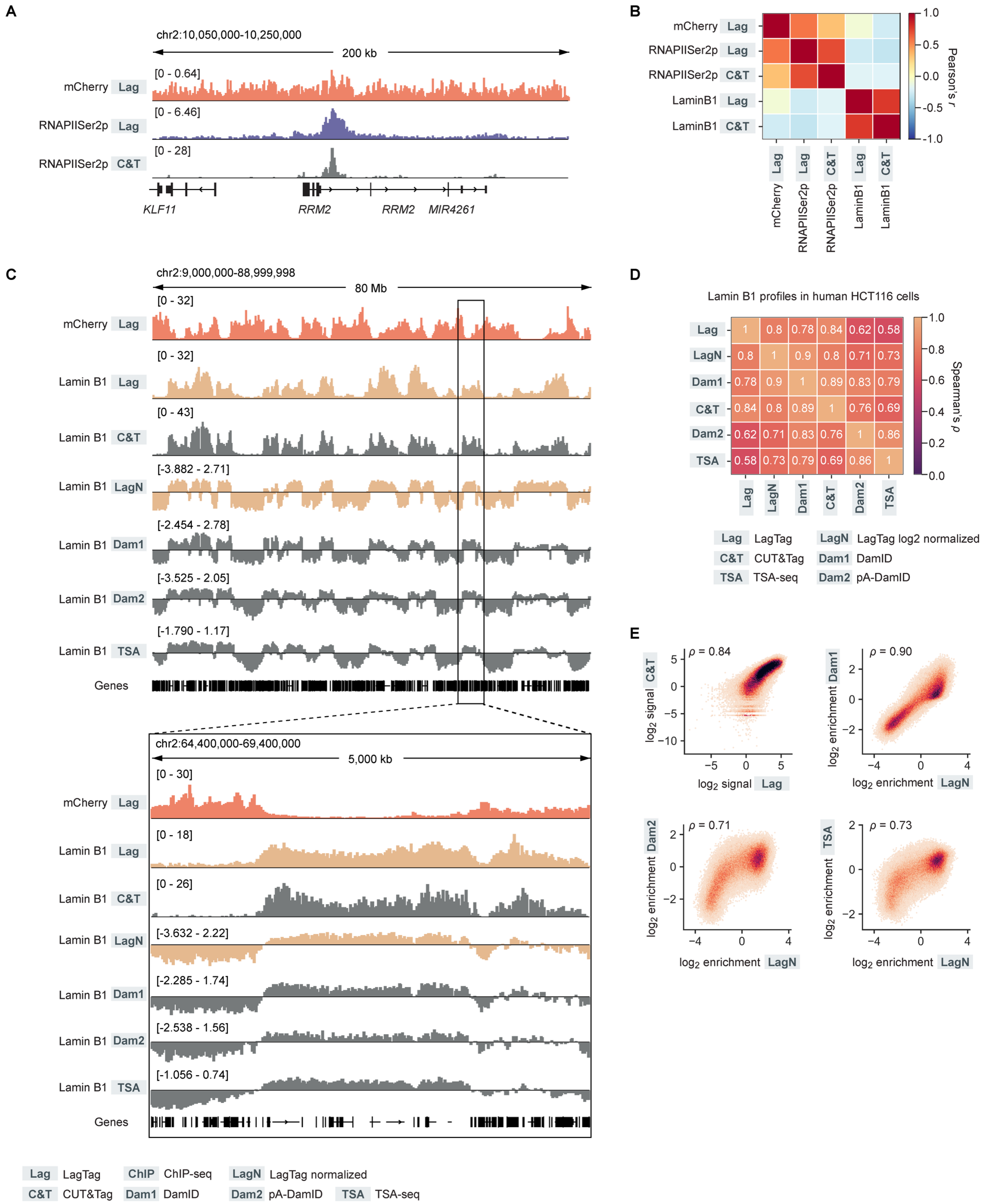

Figure S4: **Validation and characterization of LagTag profiles in human HCT116 cells**

A, Genome browser tracks of LagTag and CUT&Tag in human HCT116 cells. **B**, Correlation heatmaps of chromatin profiles for different targets as well as different profiling methods using 20-kb bins across the

genome in HCT116 cells. **C**, Genome browser tracks of LagTag, CUT&Tag, DamID[10,11], pA-DamID[12], and TSA-seq[13] in human HCT116 cells. Scale of LagTag and CUT&Tag profile was normalized to coverage per million aligned reads in **A**, **C,** or normalized to log2 ratio between Lamin B1 and mCherry profiles (LagTag normalized). The DamID and pA-DamID profiles are normalized by the Dam only control and the TSA-seq profiles are input normalized. **D**, **E**, Additional comparison of Lamin B1 profiles across different methods and normalization with commonly profiled 20-kb bins. n = 2 for CUT&Tag and LagTag experiments.

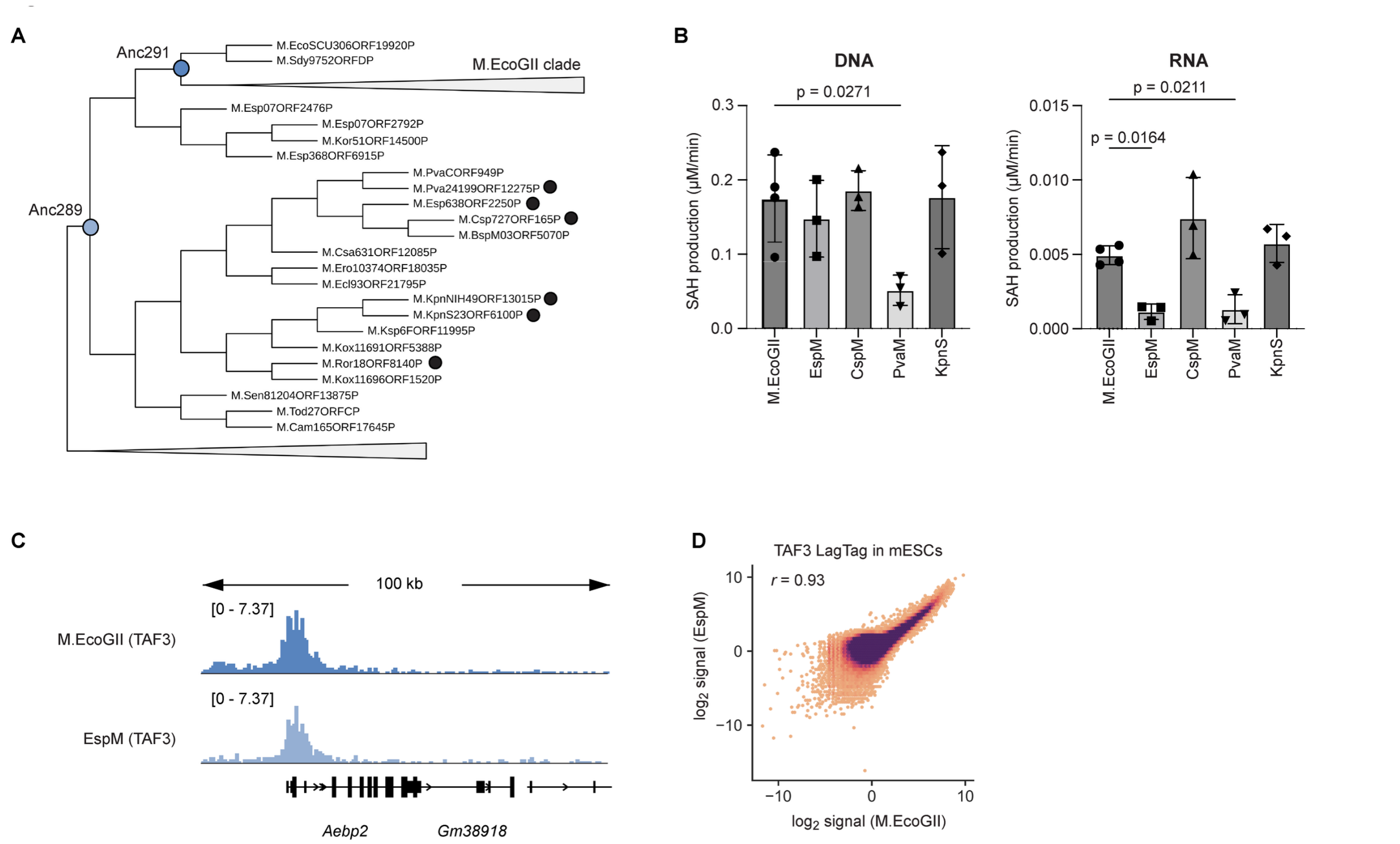

Figure S5: **Comparison of m6A methylation levels and LagTag profiles between M.EcoGII variants** **A**, Identification and selection of M.EcoGII variants. The variants with black circles were selected for protein purification (see **STAR Methods**). **B**, Comparison of in vitro methyltransferase activities on DNA (left) and RNA (right) across different M.EcoGII variants (see **STAR Methods**). EspM showed lower RNA methyltransferase activity relative to M.EcoGII in vitro. n = 4 for M.EcoGII and n = 3 for other variants. **C**, Genome browser tracks of LagTag for TAF3 in mESCs by using fusion proteins with M.EcoGII or its variant EspM. **D**, Genome-wide comparison of LagTag profiles with M.EcoGII variants expressed in mESCs using 10-kb bins. r represents Pearson correlation coefficient. n = 2 for M.EcoGII and n = 3 for EspM in **C** and **D**.

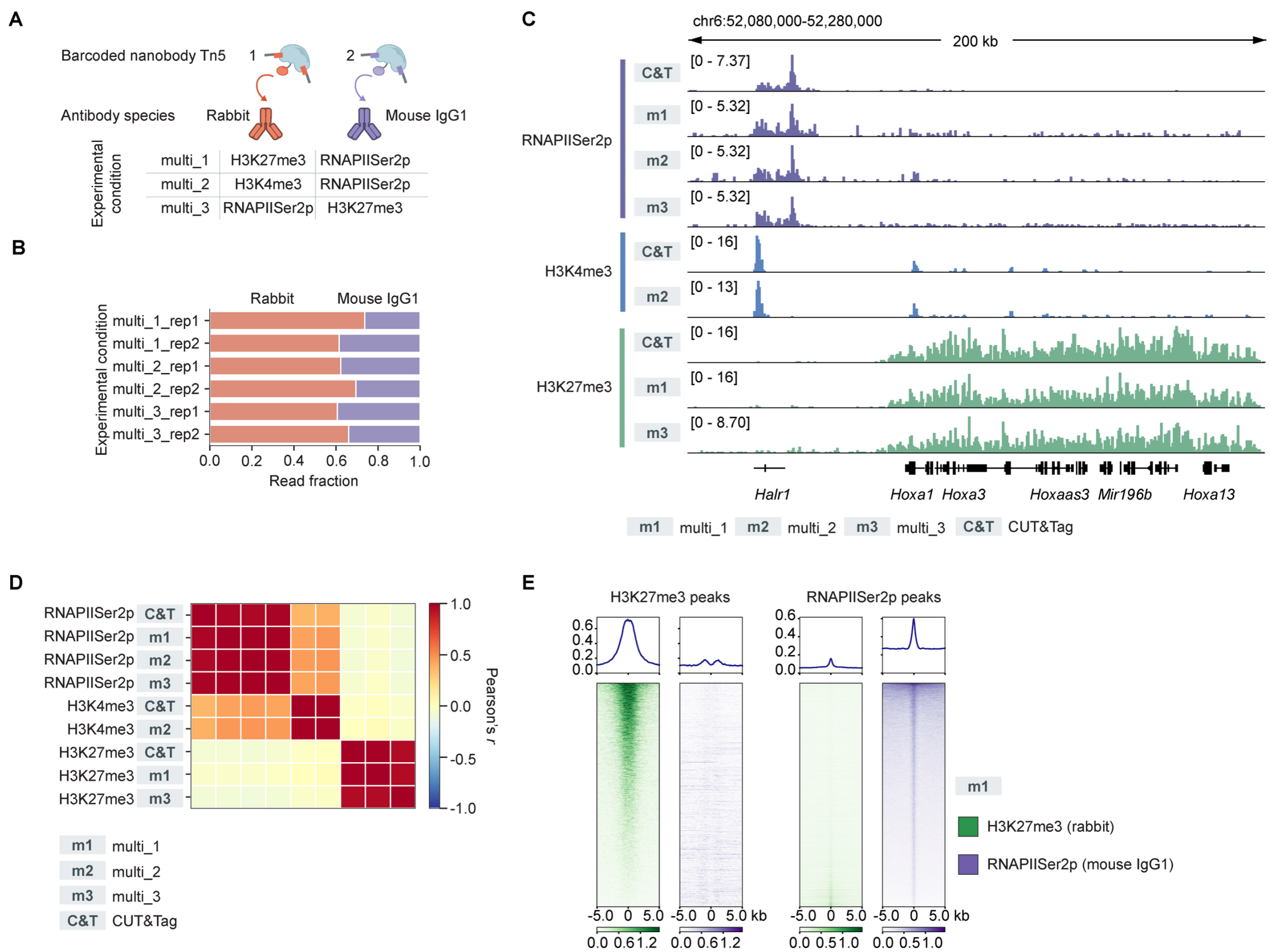

Figure S6: **Validation of multifactorial CUT&Tag in mESCs**

A, Representation of different antibody pairs used in multifactorial CUT&Tag in mESCs. The different antibodies were bound by antibody-species-specific nb-Tn5 with barcoded adapters. The assays were performed using fixed cells in 96-well plates. **B**, Comparison of read fraction from two target species after demultiplexing from each sample across conditions and replicates. The read fraction could be determined by the target abundance and antibody quality. The experimental condition and coloring of antibody species are shown in **A**. **C**, Representative genome browser tracks of CUT&Tag with non-barcoded adapters and multifactorial CUT&Tag with barcoded adapters. Scale of the profiles was normalized to coverage per million aligned reads. **D**, Correlation heatmaps of chromatin profiles for different targets using 10-kb bins across the genome. **E**, The comparison of enrichment of H3K27me3 (green) or RNAPIISer2p (purple) signal from multifactorial CUT&Tag at peaks provided by publicly available datasets (H3K27me3 ChIP-seq[6,7] and RNAPIISer2p CUT&Tag[7]). n = 2 for experiments in **B**-**E**.

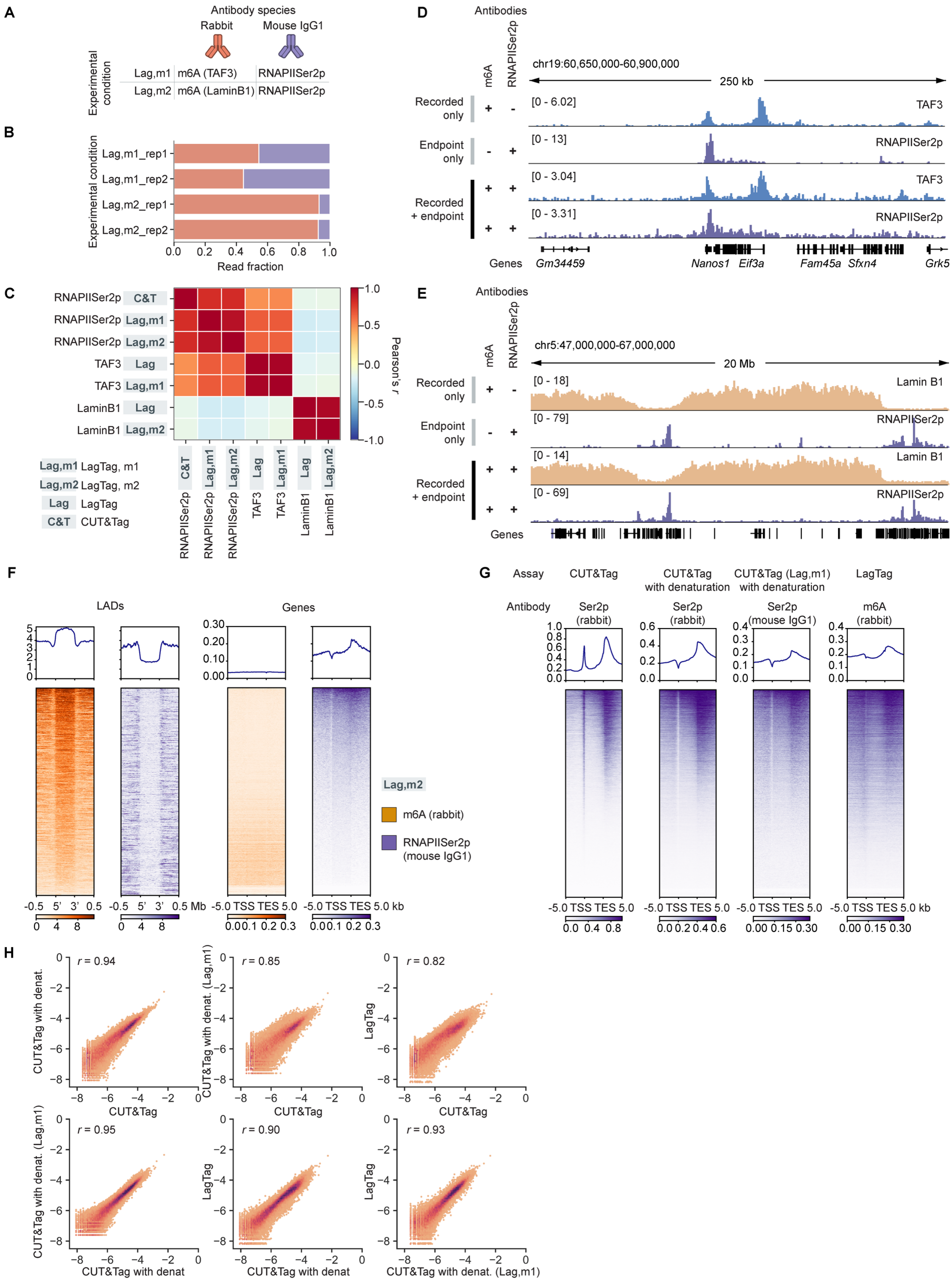

Figure S7: **Validation of recorded and endpoint LagTag profiles**

A, Representation of different antibody pairs used in LagTag assays in mESCs. Two different cell lines expressing M.EcoGII-TAF3 (reader domain) or M.EcoGII-Lamin B1 were used. **B**, Comparison of read fraction from two target species after demultiplexing from each sample across conditions and replicates. The experimental condition and coloring of antibody species are shown in **A**. **C**, Correlation heatmaps of chromatin profiles for different targets by LagTag with antibody pairs or individual assays using 10-kb bins across the genome. **D**, **E**, Representative genome browser tracks of LagTag profiles with barcoded adapters along with individual CUT&Tag and LagTag profiles with non-barcoded adapters in mESCs. Scale of the profiles was normalized to coverage per million aligned reads. 50-bp binning in **D** and 10-kb binning in **E** were used. **F**, Signal enrichment plots for LagTag condition 2 in **A** around LADs defined by DamID[8] (left panels) and around TSS and TES across all protein-coding genes (right panels) in mESCs. Scale of the profiles was normalized to coverage per million aligned reads. **G**, Signal enrichment plots of RNAPIISer2p with CUT&Tag, CUT&Tag with denaturation of 50 mM NaOH for 5 minutes, CUT&Tag with denaturation of 50 mM NaOH for 5 minutes from experimental condition 1 in **A**, and LagTag with denaturation of 500 mM NaOH for 30 minutes (from left to right) around TSS and TES across all protein-coding genes in mESCs. We note that we observed the loss of peaks at TSS upon treatments with NaOH for CUT&Tag. **H**, Scatter plots evaluating the similarity of RNAPIISer2p profiles from different assays at the gene bodies across the genome in mESCs. r represents Pearson correlation coefficient. n = 2 or n =3 (Lamin B1 LagTag, RNAPIISer2p LagTag) for CUT&Tag and LagTag experiments in **B**-**E**.

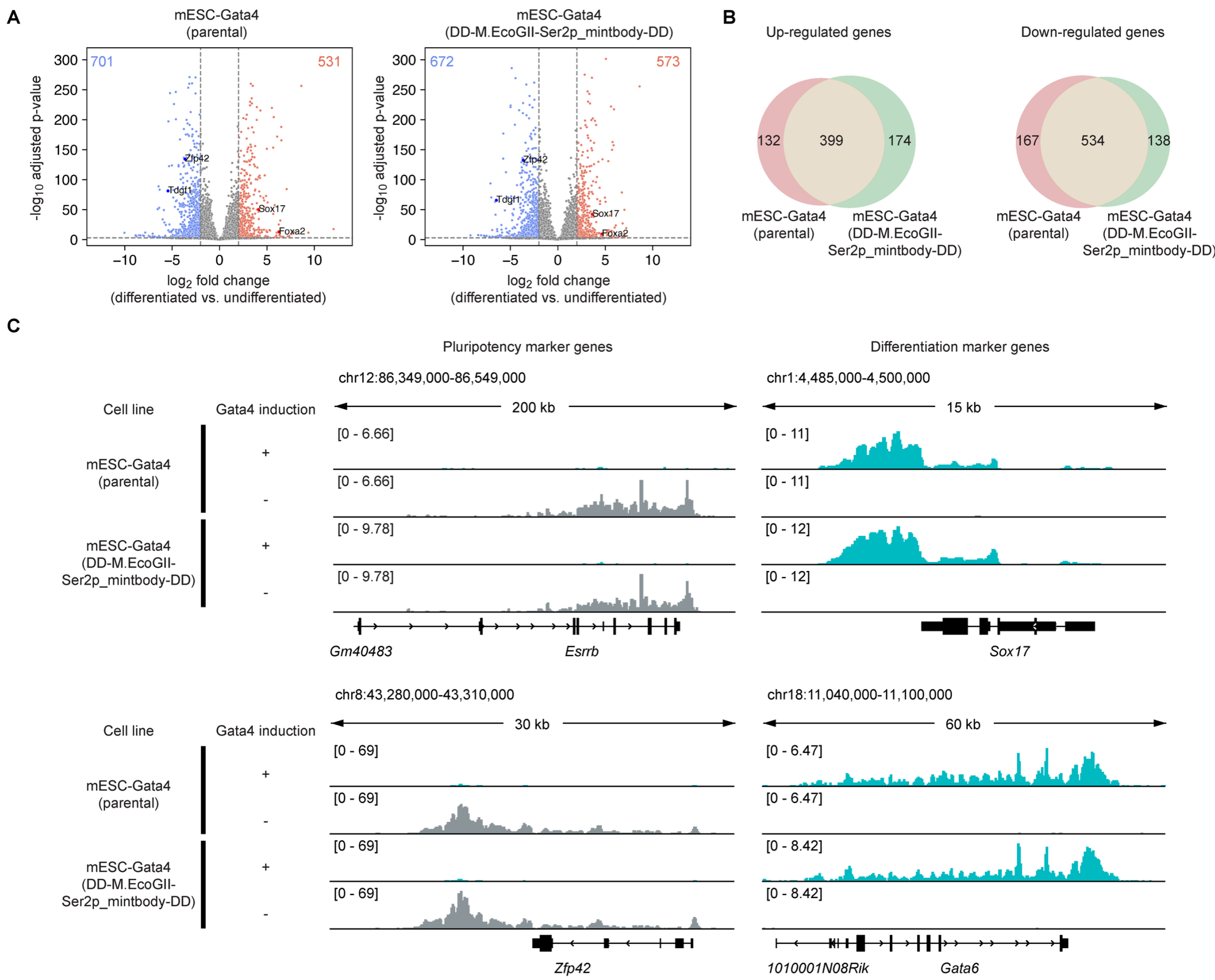

Figure S8: **Characterization of mESC differentiation with an inducible Gata4 system**

A, RNAPIISer2p CUT&Tag analysis comparing the signal enrichment over gene bodies before and after differentiation for 24 hours with the medium change in parental (left) or DD-M.EcoGII-Ser2p_mintbody-DD engineered (right) mESC-Gata4 cells. Single clones were used for these cell lines. Marker genes used in **Figure 2D, E** are highlighted with individual gene names. **B**, Up-regulated (left) and down-regulated (right) genes during the differentiation defined by RNAPIISer2p CUT&Tag in the mESC-Gata4 lines (see **STAR Methods**). **c**, Representative genome browser tracks of RNAPIISer2p CUT&Tag comparing the undifferentiated (gray) and differentiated (cyan) mESC-Gata4 cells with marker genes. n = 3 for CUT&Tag experiments.

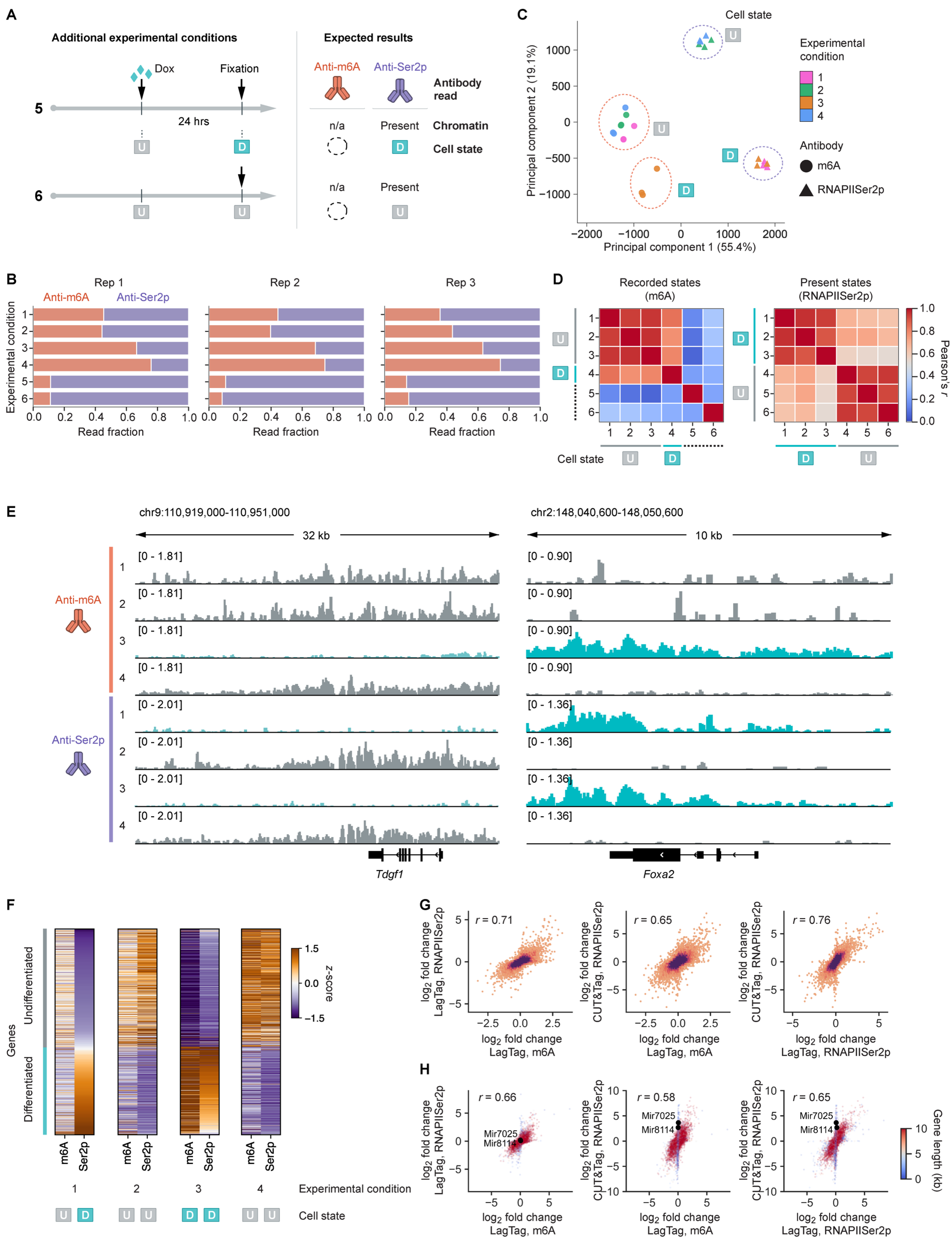

Figure S9: **Validation and characterization of LagTag profiles during mESC differentiation**

A, Additional schematic representation of LagTag experimental conditions with mESC differentiation along with expected results, corresponding to the endpoint chromatin states in each condition. The chromatin recording was not performed in these control conditions, while anti-m6A antibody was incubated with the samples. **B**, Comparison of read fraction from two target species after demultiplexing from each sample across conditions and replicates. **C**, Principal component analysis of CPM-normalized chromatin profiles (10-kb bins) colored by different experimental conditions along with the recorded and endpoint cell states. **D**, Correlation heatmaps of chromatin profiles from different experimental conditions with LagTag using 10-kb bins across the genome, along with the recorded (left) and endpoint (right) cell states. **E**, Genome browser tracks of LagTag samples across experimental conditions as well as two antibodies. The tracks are colored based on the recorded (m6A tracks) or endpoint (RNAPIISer2p tracks) cell states. **F**, Heat maps of z-score normalized LagTag profiles over genes differentially enriched with RNAPIISer2p (n = 933 genes) between undifferentiated and differentiated cell states, as determined by independent CUT&Tag experiments (n = 3). Z-score normalization was performed across conditions for each antibody (either m6A or RNAPIISer2p). Genes were sorted by RNAPIISer2p z-score enrichment in experimental condition 1. The same z-score normalized LagTag profiles, clustered by experimental conditions and genes, are shown in **Figure 2F. G**, **H**, Fold change in RNAPIISer2p signal enrichment over gene bodies during differentiation measured by LagTag (m6A or RNAPIISer2p antibody in condition 3 vs condition 4) or CUT&Tag (RNAPIISer2p only in condition 5 vs 6) in engineered mESC-Gata4 cells for coding genes (**G**) and all genes (**H**). Fold changes for some short genes were not captured by LagTag with NaOH denaturation compared with conventional CUT&Tag, potentially limiting the detection of a subset of genes by LagTag. Two representative short genes (Mir7025, Mir8114) are highlighted. n = 3 for LagTag and CUT&Tag experiments in **B**-**H**.

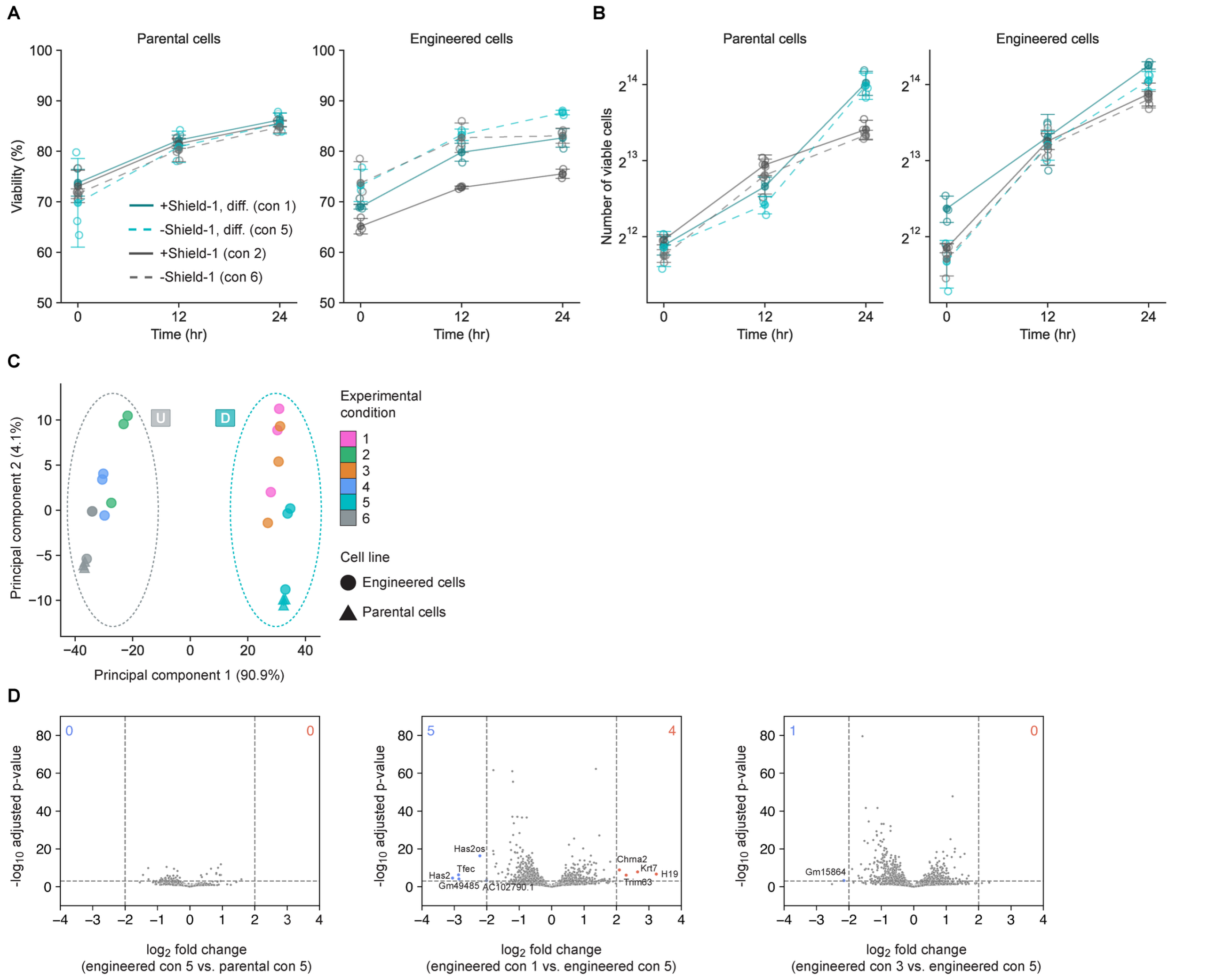

Figure S10: **Evaluation of LagTag recording effects on cell physiology during mESC differentiation** **A**, **B**, Comparison of viability (**A**) and number of viable cells (**B**) with or without prior Shield-1 induction for 16 hours in undifferentiated and differentiated cells (n = 3). **C**, Principal component analysis of RNAPIISer2p CUT&Tag profiles across different experimental conditions in engineered and parental mESC-Gata4 cells using the 500 most variable genes. **D**, RNAPIISer2p CUT&Tag analysis comparing signal enrichment over gene bodies between conditions in parental or engineered mESC-Gata4 cells. The DD-M.EcoGII-Ser2p_mintbody-DD engineered and parental mESC-Gata4 cells were used in **A**-**D**. n =3 for CUT&Tag experiments in **C** and **D**.

References:

1. Henikoff, S., Henikoff, J.G., Ahmad, K., Paranal, R.M., Janssens, D.H., Russell, Z.R., Szulzewsky, F., Kugel, S., and Holland, E.C. (2023). Epigenomic analysis of formalin-fixed paraffin-embedded samples by CUT&Tag. Nat. Commun. *14*, 5930.

2. Sobecki, M., Souaid, C., Boulay, J., Guerineau, V., Noordermeer, D., and Crabbe, L. (2018). MadID, a versatile approach to map protein-DNA interactions, highlights telomere-nuclear envelope contact sites in human cells. Cell Rep. *25*, 2891-2903.e5.

3. ENCODE Project Consortium (2012). An integrated encyclopedia of DNA elements in the human genome. Nature *489*, 57–74.

4. Abe, K., Schauer, T., and Torres-Padilla, M.-E. (2022). Distinct patterns of RNA polymerase II and transcriptional elongation characterize mammalian genome activation. Cell Rep. *41*, 111865.

5. van Schaik, T., Liu, N.Q., Manzo, S.G., Peric-Hupkes, D., de Wit, E., and van Steensel, B. (2022). CTCF and cohesin promote focal detachment of DNA from the nuclear lamina. Genome Biol. *23*, 185.

6. Mu, W., Starmer, J., Yee, D., and Magnuson, T. (2018). EZH2 variants differentially regulate polycomb repressive complex 2 in histone methylation and cell differentiation. Epigenetics Chromatin *11*, 71.

7. Gopalan, S., Wang, Y., Harper, N.W., Garber, M., and Fazzio, T.G. (2021). Simultaneous profiling of multiple chromatin proteins in the same cells. Mol. Cell *81*, 4736-4746.e5.

8. Peric-Hupkes, D., Meuleman, W., Pagie, L., Bruggeman, S.W.M., Solovei, I., Brugman, W., Gräf, S., Flicek, P., Kerkhoven, R.M., van Lohuizen, M., et al. (2010). Molecular maps of the reorganization of genome-nuclear lamina interactions during differentiation. Mol. Cell *38*, 603–613.

9. Redolfi, J., Zhan, Y., Valdes-Quezada, C., Kryzhanovska, M., Guerreiro, I., Iesmantavicius, V., Pollex, T., Grand, R.S., Mulugeta, E., Kind, J., et al. (2019). DamC reveals principles of chromatin folding in vivo without crosslinking and ligation. Nat. Struct. Mol. Biol. *26*, 471–480.

10. Dekker, J., Belmont, A.S., Guttman, M., Leshyk, V.O., Lis, J.T., Lomvardas, S., Mirny, L.A., O'Shea, C.C., Park, P.J., Ren, B., et al. (2017). The 4D nucleome project. Nature *549*, 219–226.

11. Reiff, S.B., Schroeder, A.J., Kırlı, K., Cosolo, A., Bakker, C., Mercado, L., Lee, S., Veit, A.D., Balashov, A.K., Vitzthum, C., et al. (2022). The 4D Nucleome Data Portal as a resource for searching and visualizing curated nucleomics data. Nat. Commun. *13*, 2365.

12. van Schaik, T., Vos, M., Peric-Hupkes, D., Hn Celie, P., and van Steensel, B. (2020). Cell cycle dynamics of lamina-associated DNA. EMBO Rep. *21*, e50636.

13. Kumar, P., Gholamalamdari, O., Zhang, Y., Zhang, L., Vertii, A., van Schaik, T., Peric-Hupkes, D., Sasaki, T., Gilbert, D.M., van Steensel, B., et al. (2024). Nucleolus and centromere Tyramide Signal Amplification-Seq reveals variable localization of heterochromatin in different cell types. Commun. Biol. *7*, 1135.
